# Disorder-induced nonlinear Hall effect with time-reversal symmetry

**DOI:** 10.1038/s41467-019-10941-3

**Published:** 2019-07-11

**Authors:** Z. Z. Du, C. M. Wang, Shuai Li, Hai-Zhou Lu, X. C. Xie

**Affiliations:** 1 Shenzhen Institute for Quantum Science and Engineering and Department of Physics, Southern University of Science and Technology, Shenzhen, 518055 China; 2 Shenzhen Key Laboratory of Quantum Science and Engineering, Shenzhen, 518055 China; 3 Peng Cheng Laboratory, Shenzhen, 518055 China; 4 Department of Physics, Shanghai Normal University, Shanghai, 200234 China; 5 Tsung-Dao Lee Institute, Shanghai Jiao Tong University, Shanghai, 200240 China; 6 International Center for Quantum Materials, School of Physics, Peking University, Beijing, 100871 China; 7 Beijing Academy of Quantum Information Sciences, Beijing, 100193 China; 8 CAS Center for Excellence in Topological Quantum Computation, University of Chinese Academy of Sciences, Beijing, 100190 China

**Keywords:** Electronic properties and materials, Two-dimensional materials, Topological insulators

## Abstract

The nonlinear Hall effect has opened the door towards deeper understanding of topological states of matter. Disorder plays indispensable roles in various linear Hall effects, such as the localization in the quantized Hall effects and the extrinsic mechanisms of the anomalous, spin, and valley Hall effects. Unlike in the linear Hall effects, disorder enters the nonlinear Hall effect even in the leading order. Here, we derive the formulas of the nonlinear Hall conductivity in the presence of disorder scattering. We apply the formulas to calculate the nonlinear Hall response of the tilted 2D Dirac model, which is the symmetry-allowed minimal model for the nonlinear Hall effect and can serve as a building block in realistic band structures. More importantly, we construct the general scaling law of the nonlinear Hall effect, which may help in experiments to distinguish disorder-induced contributions to the nonlinear Hall effect in the future.

## Abstract (web summary)

Disorder may play a dominant role in determining the nonlinear Hall effect in a topological material. Here, Du et al. derive formulas of the nonlinear Hall conductivity and construct the general scaling law of the nonlinear Hall effect in a tilted two dimensional Dirac model.

## Introduction

**Fig. 1:**
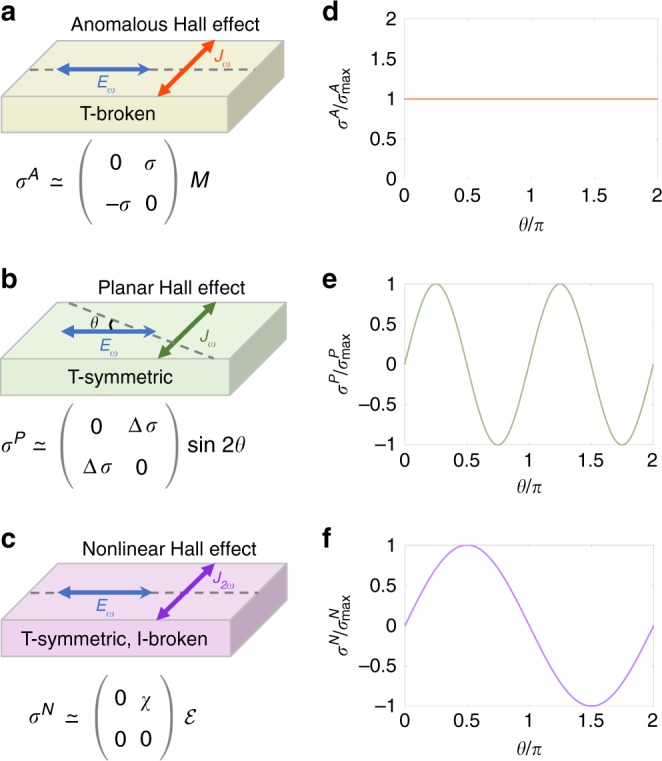
Comparison of the linear and nonlinear Hall effects in the absence of the magnetic field. Experimental setups and time-reversal symmetry of the anomalous (**a**), planar (**b**), and nonlinear Hall effects (**c**). *σ*^*A*^ is the anomalous Hall conductivity, which is always anti-symmetric^3^. *M* represents the magnetization. *σ*^*P*^ is the planar Hall conductivity. ${\mathrm{\Delta }}\sigma \equiv \sigma _\parallel - \sigma _ \bot$, where $\sigma _\parallel$ and $\sigma _ \bot$ refer to the longitudinal conductivities along the two principal axes. *θ* is the angle between the driving current and the $\parallel$ principal axis (the dashed lines). *σ*^*N*^ is the nonlinear Hall conductivity, which is proportional to the magnitude of the driving electric field ${\cal{E}}$. The element of the nonlinear Hall response tensor $\chi$ is due to inversion symmetry breaking along the dashed line. **d**–**f** Angular dependence can be used to distinguish the anomalous, planar, and nonlinear Hall effects

The Hall effects refer to a transverse voltage in response to a current applied in a sample of metal or semiconductor. The family of the classical and quantized Hall effects is one of the mainstreams of modern condensed matter physics, leading to the full spectrum of the search on the topological states of matter and many practical applications^1,2^. All previous Hall effects are in the linear-response regime, that is, the transverse voltage is linearly proportional to the driving current, and a measurable Hall voltage requires that time-reversal symmetry is broken by magnetic fields or magnetism^1–4^. The recently discovered nonlinear Hall effect^5–13^ does not need time-reversal symmetry breaking but inversion symmetry breaking, significantly different from the known linear Hall effects (Fig. 1). The linear Hall effects can be understood in terms of the Berry curvature^14^, which describes bending of a parameter space (real space, momentum space, any vector fields)^15^. This geometric description is of the same significance as the curved spacetime in the general theory of relativity. The nonlinear Hall effect depends on the higher-order properties of the Berry curvature, thus not only can bring our knowledge to the next level but also may help device applications. More importantly, by adjusting the measurements to the nonlinear regime, unconventional transport phenomena can be explored in a great number of emergent materials in which discrete and crystal symmetries are broken.

The disorder effects have been a large part of the research on the linear Hall effects, such as the localization in the quantized Hall effects^16,17^, the extrinsic mechanisms of the anomalous^3^, spin^18^, and valley^19^ Hall effect, etc. The debate on the origin of the anomalous Hall effect lasted for one century, until recently the mechanisms are summarized in terms of intrinsic (disorder-free) and extrinsic (disorder-induced) contributions^3^. The quantitative agreement between theories and experiments shows that the disorder-induced contribution is comparably important^20,21^. In the nonlinear Hall effect, disorder is more important, because the effect always requires that the Fermi energy crosses an energy band, while on the Fermi surface the disorder scattering is inevitable and enters the nonlinear Hall effect even in the leading order. This is quite different from the disorder-free leading order in the linear Hall effects. How disorder contributes to the nonlinear Hall signal in a specific form remains unknown and is the focus of the investigations at this stage.

In this work, we use the Boltzmann formalism to derive the formulas of the nonlinear Hall conductivity in the presence of disorder scattering. The formulas can be applied to different models to calculate the nonlinear Hall responses. We apply the formula to the 2D tilted massive Dirac model. The model is a symmetry-allowed minimal model for the nonlinear Hall effect and can be used to understand the nonlinear Hall signals in realistic band structures^12^. Depending on roles of disorder scattering, we follow the convention to classify the nonlinear Hall conductivity into the intrinsic, side-jump, and skew-scattering contributions. The latter two are new findings to the framework of the nonlinear Hall effect and comparably important. The competition between the three contributions can induce a sign change in the nonlinear Hall signal. More importantly, we present the scaling laws of the nonlinear Hall effect, which help to identify distinct contributions and explain the temperature and thickness dependence in the experiments in the future.

## Results

### General formulas

The nonlinear Hall effect is measured as zero- and double-frequency transverse electric currents driven by a low-frequency *ac* longitudinal electric field ${\mathbf{J}}({\mathbf{E}}) = - e\mathop {\sum}\nolimits_l {\dot{\mathbf{r}}}_lf_l$ in the absence of a magnetic field, where the *ac* electric field ${\mathbf{E}}(t) = {\mathrm{Re}}\{ {\cal{E}}e^{i\omega t}\}$ with the amplitude vector ${\cal{E}}$ and frequency $\omega$. Here −*e* is the electron charge, $l = (\eta ,{\mathbf{k}})$ labels a state in band $\eta$ with wave vector **k** and *f*_*l*_ is the corresponding distribution function. The current up to the second-order of the *ac* electric field can be found as $J_a = {\mathrm{Re}}\{ J_a^{(0)} + J_a^{(1)}e^{i\omega t} + J_a^{(2)}e^{i2\omega t}\}$, with

1 $$J_a^{(0)} = \xi _{abc}{\cal{E}}_b{\cal{E}}_c^ \ast ,\,J_a^{(1)} = \sigma _{ab}{\cal{E}}_b,\,J_a^{(2)} = \chi _{abc}{\cal{E}}_b{\cal{E}}_c,$$

respectively, where $\{ a,b,c\} \in \{ x,y,z\}$. Table 1 summarizes our main results for the anomalous Hall response tensor *σ*_*ab*_ and the double-frequency nonlinear Hall response tensor $\chi _{abc}$ (see Methods). We have assumed that $\omega \tau \ll 1$, because $\omega$ is about tens of Hertz and the relaxation time $\tau$ is about picoseconds in experiments. This low-frequency limit is one of the differences from the nonlinear optics. The disorder-induced zero-frequency response $\xi _{abc}$ is identical with the double-frequency response $\chi _{abc}$ in the $\omega \tau \ll 1$ limit. Away from the $\omega \tau \ll 1$ limit, the double- and zero-frequency nonlinear Hall conductivities have different frequency dependence, thus are different in general. For a complete description, we list the $\omega$-dependent full expressions with and without time-reversal symmetry (Supplementary Note 3), which would be helpful for understanding the recently proposed high-frequency rectification^22^ and gyrotropic Hall effects^23^. According to how disorder works, the formulas are classified in terms of intrinsic (*in*), side-jump (*sj*), skew-scattering (*sk*) contributions. The formulas in Table 1 can be applied to different models to calculate the nonlinear Hall responses.

**Table 1:** Formulas of the anomalous and nonlinear Hall responses in the $\omega \tau \ll 1$ limit

	Anomalous Hall response (*e*^2^/*ħ*)	Nonlinear Hall response (*e*^3^/2*ħ*^2^)
Time-reversal symmetry	Broken	Preserved
Intrinsic	$\sigma _{ab}^{in} = - \mathop {\sum}\nolimits_l \varepsilon ^{abc}{\mathrm{\Omega }}_l^cf_l^{(0)}$	$\chi _{abc}^{in} = - \mathop {\sum}\nolimits_l \varepsilon ^{acd}{\mathrm{\Omega }}_l^dg_l^b$
Side-jump (velocity)	$\sigma _{ab}^{sj,1} = - \mathop {\sum}\nolimits_l v_a^{sj}g_l^b$	$\chi _{abc}^{sj,1} = - \mathop {\sum}\nolimits_l \tau _lv_a^{sj}\partial _cg_l^b$
Side-jump (distribution)	$\sigma _{ab}^{sj,2} = \mathop {\sum}\nolimits_l v_b^{sj}g_l^a$	$\chi _{abc}^{sj,2} = - \hbar \mathop {\sum}\nolimits_l \tau _l\{ [\partial _a(\tau _lv_c^{sj}) + \tilde {\cal{M}}_l^{ac}]v_l^b + \partial _c(\tau _lv_l^a)v_b^{sj}\} \frac{{\partial f_l^{(0)}}}{{\partial \varepsilon _l}}$
Intrinsic skew-scattering	$\sigma _{ab}^{sk,1} = - \mathop {\sum}\nolimits_{ll\prime } \varpi _{ll\prime }^g{\cal{U}}_{ll\prime }^ag_l^b$	$\chi _{abc}^{sk,1} = \mathop {\sum}\nolimits_{ll\prime } \varpi _{ll\prime }^g(\tilde {\cal{U}}_{ll\prime }^{ca} - \tau _l{\cal{U}}_{ll\prime }^a\partial _c)g_l^b$
Extrinsic skew-scattering	$\sigma _{ab}^{sk,2} = - \mathop {\sum}\nolimits_{ll\prime } \varpi _{ll\prime }^{ng}{\cal{U}}_{ll\prime }^ag_l^b$	$\chi _{abc}^{sk,2} = \mathop {\sum}\nolimits_{ll\prime } \varpi _{ll\prime }^{ng}(\tilde {\cal{U}}_{ll\prime }^{ca} - \tau _l{\cal{U}}_{ll\prime }^a\partial _c)g_l^b$

We refer to the leading-order of the nonlinear Hall conductivity as the intrinsic contribution, but it depends on the disorder scattering, quite different from the disorder-free intrinsic Hall conductivity. The side-jump and skew-scattering contributions are due to the coordinates shift and antisymmetric scattering, respectively. Here $\varepsilon ^{abc}$ is the anti-symmetric tensor, we define $\partial _a \equiv \partial /\partial k_a$, $\partial _a^\prime \equiv \partial /\partial k_a^\prime$, and $g_l^a \equiv \tau _l\partial _af_l^{(0)}$. $\tau _l$ is the general relaxation time and $f_l^{(0)}$ is the Fermi distribution. The Berry curvature^3, 15^ ${\mathrm{\Omega }}_l^a$ = −$2\varepsilon ^{abc}\mathop {\sum}\nolimits_{l\prime \ne l} {\mathrm{Im}}\langle l|\partial _b\hat {\cal{H}}|l\prime \rangle$ $\langle l\prime |\partial _c\hat {\cal{H}}|l\rangle$/$(\varepsilon _l - \varepsilon _{l\prime })^2$, where $|l\rangle$ is the eigenvector. The side-jump velocity $v_a^{sj} = \mathop {\sum}\nolimits_{l\prime } \varpi _{ll\prime }^{sy}\delta r_{l\prime l}^a$ and $\tilde {\cal{M}}_l^{ab} \equiv \mathop {\sum}\nolimits_{l\prime }$ $(\tilde M_{ll\prime }^{ac} - \tilde M_{l\prime l}^{ac})\delta (\varepsilon _l - \varepsilon _{l\prime })$, where $\varpi _{ll\prime }^{sy}$ is the symmetric scattering rate, the coordinates shift^28^, $\delta r_{ll\prime }^a$ = $i\langle l|\partial _a|l\rangle$ − $i\langle l\prime |\partial _a^\prime |l\prime \rangle$ − $(\partial _a + \partial _a^\prime )$ ${\mathrm{arg}}(V_{ll\prime })$ with $V_{ll\prime } \equiv \langle l|\hat V_{imp}|l\prime \rangle$ and $\tilde M_{ll\prime }^{ab} \equiv (2\pi /\hbar )\partial _a(\tau _l|T_{ll\prime }|^2\delta r_{ll\prime }^b)$. $\varpi _{ll\prime }^g$ and $\varpi _{ll\prime }^{ng}$ refer to the Gaussian and non-Gaussian antisymmetric scattering rate, ${\cal{U}}_{ll\prime }^a \equiv \tau _lv_l^a - \tau _{l\prime }v_{l\prime }^a$ and $\tilde {\cal{U}}_{ll{\prime}}^{ab} \equiv \tau _l\partial _a(\tau _lv_l^b) - \tau _{l\prime }\partial _a^\prime (\tau _{l\prime }v_{l\prime }^b)$

### Tilted 2D massive Dirac model

Now we apply Table 1 to calculate the nonlinear Hall conductivity in the presence of disorder scattering, for the tilted 2D massive Dirac model (see Methods). The model gives the symmetry-allowed minimal description of the nonlinear Hall effect and can serve as a building block in realistic band structures^12^

2 $$\hat {\cal{H}} = tk_x + v(k_x\sigma _x + k_y\sigma _y) + m\sigma _z,$$

where $(k_x,k_y)$ are the wave vectors, $(\sigma _x,\sigma _y,\sigma _z)$ are the Pauli matrices, *t*, *v*, and *m* are model parameters. *t* tilts the Dirac cone along the *x* direction. The time reversal of the model contributes equally to the Berry dipole, so it is enough to study this model only. For the disorder part, we assume a *δ*-correlated spin independent random potential $\hat V_{imp}({\mathbf{r}}) = \mathop {\sum}\nolimits_i V_i\delta ({\mathbf{r}} - {\mathbf{R}}_i)$ with both Gaussian $\left\langle {V_i^2} \right\rangle _{dis} = V_0^2$ and non-Gaussian correlations $\left\langle {V_i^3} \right\rangle _{dis} = V_1^3$.

**Fig. 2:**
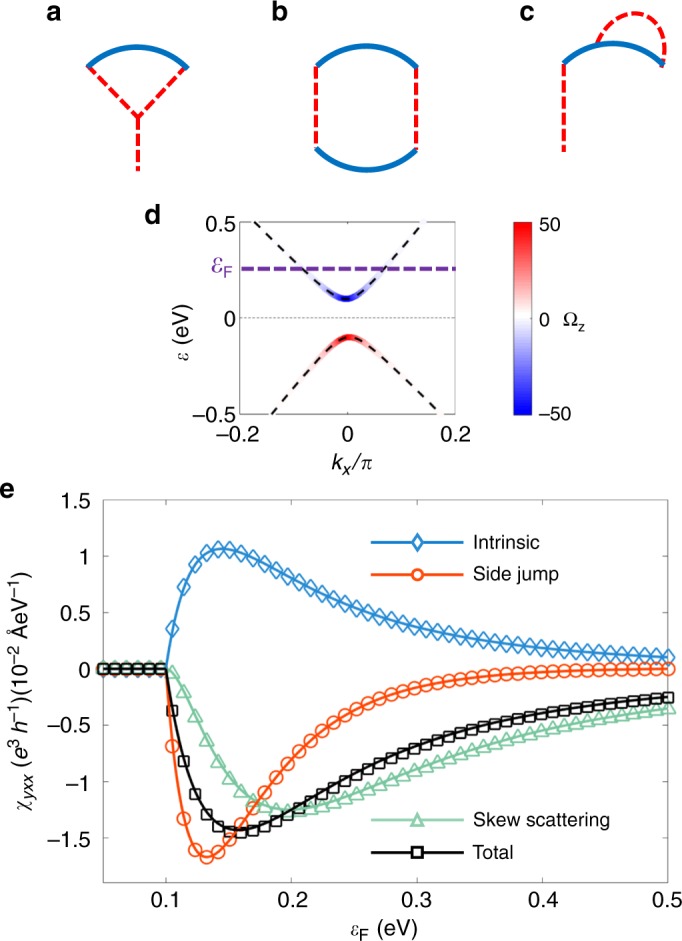
Nonlinear Hall response of 2D tilted massive Dirac model. Terms contributing to the antisymmetric part of the scattering rate $\varpi _{ll\prime }^{(3)}$ (**a**) and $\varpi _{ll\prime }^{(4)}$ (**b**, **c**). **d** The band structure with the intensity plot of the Berry curvature ${\mathrm{\Omega }}_z$. **e** The intrinsic, side-jump, skew-scattering, and total contributions to nonlinear Hall conductivity $\chi _{yxx}$ of the 2D tilted massive Dirac model at zero temperature with a constant relaxation time $\tau$. The markers are the numerical results and the solid lin es are analytic results up to leading $t$. Parameters are chosen as $t = 0.1\,{\mathrm{eV}} \cdot {\mathrm{{\AA}}}$, $v = 1\,{\mathrm{eV}} \cdot {\mathrm{{\AA}}}$, $m = 0.1\,{\mathrm{eV}}$, $n_iV_0^2 = 10^2\,{\mathrm{eV}}^2 \cdot {\mathrm{{\AA}}}^2$ and $n_iV_1^3 = 10^4\,{\mathrm{eV}}^3 \cdot {\mathrm{{\AA}}}^4$

To have analytic expressions with intuitive insight, we assume that $t \ll v$ and the relaxation time $\tau$ is *k*-independent, i.e., $1/\tau = n_iV_0^2(\varepsilon _{\mathrm{F}}^2 + 3m^2)/(4\hbar v^2\varepsilon _{\mathrm{F}})$, where *n*_*i*_ is the impurity density (Supplementary Note 4). As functions of the Fermi energy $\varepsilon _{\mathrm{F}}$, we obtain the analytic expressions for the intrinsic

3 $$\chi _{yxx}^{in} = \frac{{e^3}}{h}\frac{{tm}}{{n_iV_0^2}}\frac{{3v^2(\varepsilon _{\mathrm{F}}^2 - m^2)}}{{2\varepsilon _{\mathrm{F}}^3(\varepsilon _{\mathrm{F}}^2 + 3m^2)}},$$

side-jump

4 $$\chi _{yxx}^{sj} = \frac{{e^3}}{h}\frac{{tm}}{{n_iV_0^2}}\frac{{v^2(\varepsilon _F^2 - m^2)(\varepsilon _{\mathrm{F}}^2 - 25m^2)}}{{2\varepsilon _{\mathrm{F}}^3(\varepsilon _{\mathrm{F}}^2 + 3m^2)^2}},$$

and skew-scattering response functions

5 $$\begin{array}{*{20}{l}} {\chi _{yxx}^{sk,1}} \hfill & = \hfill & { - \frac{{e^3}}{h}\frac{{tm}}{{n_iV_0^2}}\frac{{v^2(\varepsilon _F^2 - m^2)^2(13\varepsilon _{\mathrm{F}}^2 + 77m^2)}}{{4\varepsilon _{\mathrm{F}}^3(\varepsilon _{\mathrm{F}}^2 + 3m^2)^3}},} \hfill \\ {\chi _{yxx}^{sk,2}} \hfill & = \hfill & { - \frac{{e^3}}{h}\frac{{tm}}{{n_i^2V_0^6/V_1^3}}\frac{{v^2(\varepsilon _F^2 - m^2)^2(5\varepsilon _{\mathrm{F}}^2 + 9m^2)}}{{\varepsilon _{\mathrm{F}}^2(\varepsilon _{\mathrm{F}}^2 + 3m^2)^3}}} \hfill \end{array}$$

up to the linear order in *t*. $\chi _{xyy} = 0$ for each contribution, as required by mirror reflection symmetry $k_y \leftrightarrow - k_y$. According to the above analytic expressions, the side-jump ($\chi ^{sj}$) and intrinsic skew-scattering ($\chi ^{sk,1}$) contributions are of the same order with the intrinsic one ($\chi ^{in}$). The extrinsic skew-scattering ($\chi ^{sk,2}$) contribution is controlled by the relative scattering strength of the non-Gaussian scattering $V_1^3$. The factor $\varepsilon _{\mathrm{F}}^2 - m^2$ in all the contributions secures that the nonlinear Hall conductivity vanishes at the band edge. It is interesting to note that the side-jump contribution dominates near the bottom of the band, which is consistent with the result of a recent work^24^. At higher $\varepsilon _F$, the skew-scattering becomes the strongest contribution, which is similar to the behaviors in the anomalous Hall effect. All contributions vanish as $\varepsilon _F \to \infty$. These behaviors can be seen in Fig. 2e.

The zero-frequency nonlinear Hall response was not addressed experimentally. In the $\omega \tau \ll 1$ limit, $\xi _{yxx} = \chi _{yxx}$. According to symmetry, $\xi _{xyy} = 0$. In the *dc* limit ($\omega = 0$), the electric field becomes time independent $E_a(t) = {\cal{E}}_a$, and the nonlinear Hall response becomes a *dc* current $J_a = (\xi _{abc} + \chi _{abc}){\cal{E}}_b{\cal{E}}_c = 2\chi _{abc}{\cal{E}}_b{\cal{E}}_c$, which means that for the Dirac model tilted along the *x* direction [Eq. (2)], an *x*-direction electric field can generate a *dc* nonlinear Hall current along the *y* direction. As a result, the measured Hall conductivity will be proportional to the electric field

6 $$\sigma _{yx}^N = 2\chi _{yxx}{\cal{E}}_x.$$

In contrast, if the electric field is along the *y* direction, there is no such a Hall signal because $\chi _{xyy} = 0$, as required by the *y*-direction mirror reflection symmetry. This indicates that the *dc* nonlinear Hall signal $\sigma _{xy}^N$ has one-fold angular dependence^11,12^. This *dc* Hall signal can exist in the presence of time-reversal symmetry, which has been observed in the nonmagnetic Weyl–Kondo semimetal Ce_3_Bi_4_Pd_3_^25^.

### Scaling law of the nonlinear Hall effect

It is of fundamental importance to distinguish the different contributions to the nonlinear Hall signal in experiments. For the anomalous Hall effect, distinguishing different contributions is based on the scaling law of the transverse Hall signal to the longitudinal signal^3,20,21,26^. For the nonlinear Hall effect, a scaling law can be constructed as well. We adopt the quantity $V_y^N/(V_x^L)^2 = \xi _{yxx}\rho _{xx}$ or $\chi _{yxx}\rho _{xx}$ as the experimental scaling variable^11,12^, where $V_y^N$ and $V_x^L$ refer to the nonlinear Hall (zero- or double-frequency) and linear longitudinal voltage, respectively. To measure the nonzero $\chi _{yxx}$, the driving electric current is applied along the *x* direction and the nonlinear Hall voltage is measured along the *y* direction. An advantage of this variable is that the intrinsic and side-jump parts become disorder independent.

To account for multiple sources of scattering^21,26^, we consider the scaling law of nonlinear Hall effect in a general manner. For simplicity, we assume no correlation between different scattering sources, thus each source contributes to the total resistivity independently, as dictated by Matthiessen's rule $\rho _{xx} = \mathop {\sum}\nolimits_i \rho _i$^27^, where $\rho _i$ is the contribution of the *i*th type of disorder scattering to the longitudinal resistivity. According to Table 1, the general scaling law of the nonlinear Hall effect can be obtained as (Supplementary Note 5)

7 $$\frac{{V_y^N}}{{(V_x^L)^2}} = {\cal{C}}^{in} + \mathop {\sum}\limits_i {\cal{C}}_i^{sj}\frac{{\rho _i}}{{\rho _{xx}}} + \mathop {\sum}\limits_{ij} {\cal{C}}_{ij}^{sk,1}\frac{{\rho _i\rho _j}}{{\rho _{xx}^2}} + \mathop {\sum}\limits_{i \in S} {\cal{C}}_i^{sk,2}\frac{{\rho _i}}{{\rho _{xx}^2}}.$$

Here the disorder-independent coefficients are for the intrinsic (${\cal{C}}^{in}$), side-jump (${\cal{C}}_i^{sj}$), intrinsic skew-scattering (${\cal{C}}_i^{sk,1}$), and extrinsic skew-scattering (${\cal{C}}_{ij}^{sk,2}$) contributions, respectively. *S* stands for static disorder scattering sources^21^. To use Eq. (7), one needs to specify scattering sources. As an example, we consider two major scattering sources^21^, one static ($i = 0$) and one dynamic ($i = 1$), then the scaling law becomes

8 $$\frac{{V_y^N}}{{(V_x^L)^2}} = \frac{1}{{\rho _{xx}^2}}({\cal{C}}_1\rho _{xx0} + {\cal{C}}_2\rho _{xx0}^2 + {\cal{C}}_3\rho _{xx0}\rho _{xxT} + {\cal{C}}_4\rho _{xxT}^2),$$

with four scaling parameters

9 $$\begin{array}{*{20}{l}} {{\cal{C}}_1 = {\cal{C}}^{sk,2},\,{\cal{C}}_2 = {\cal{C}}^{in} + {\cal{C}}_0^{sj} + {\cal{C}}_{00}^{sk,1},} \hfill \\ {{\cal{C}}_3 = 2{\cal{C}}^{in} + {\cal{C}}_0^{sj} + {\cal{C}}_1^{sj} + {\cal{C}}_{01}^{sk,1},} \hfill \\ {{\cal{C}}_4 = {\cal{C}}^{in} + {\cal{C}}_1^{sj} + {\cal{C}}_{11}^{sk,1}.} \hfill \end{array}$$

${\cal{C}}_{1,2,3,4}$ can be extracted from experiments^20,21,26^. Here $\rho _{xx0}$ is the residual resistivity due to static impurities at zero temperature and $\rho _{xxT} \equiv \rho _{xx} - \rho _{xx0}$ is due to dynamic disorders (e.g., phonons) at finite temperature.

**Fig. 3:**
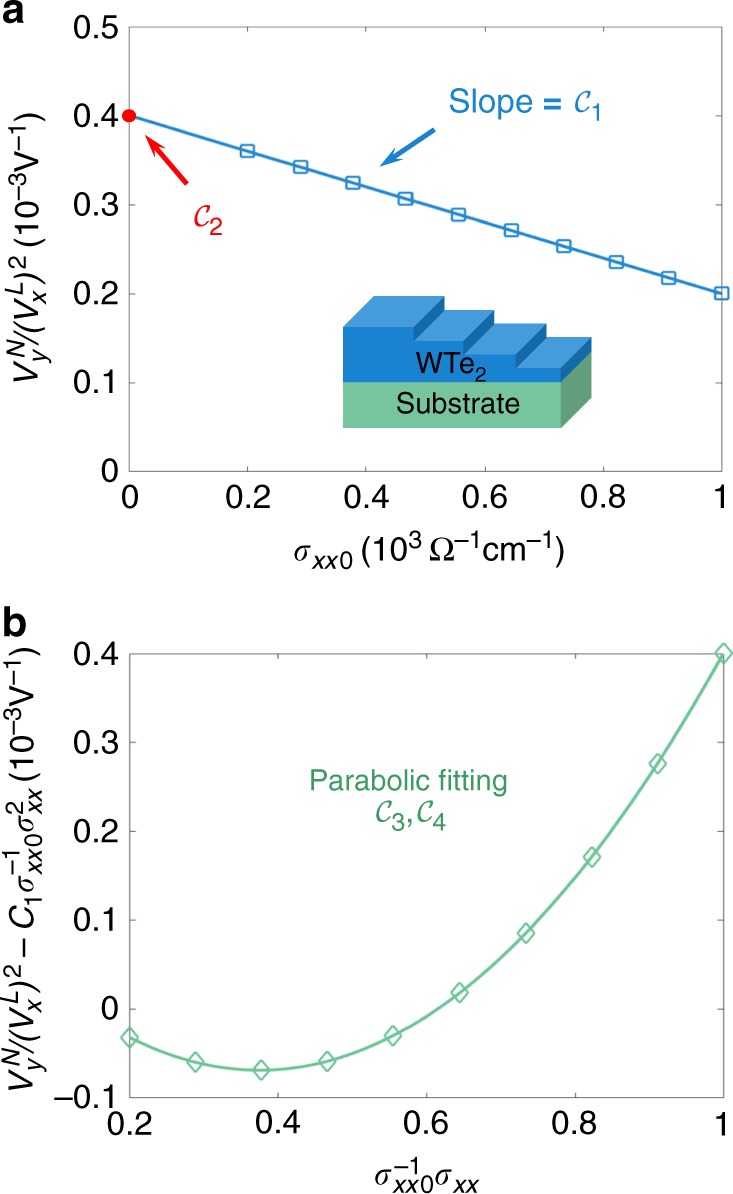
Scaling law of the nonlinear Hall effect. **a** Step 1. At zero temperature, fitting ${\cal{C}}_1$ and ${\cal{C}}_2$ with the data of $V_y^N/(V_x^L)^2$ and *σ*_*xx*0_ for samples of different disorder strength (e.g., by changing the thickness^20,21^). Insert is the schematic of the WTe_2_ multi-step sample. **b** Step 2. At finite temperatures, for a given sample of known *σ*_*xx*0_, fitting ${\cal{C}}_3$ and ${\cal{C}}_4$ with the data of *σ*_*xx*_ at different temperatures. ${\cal{C}}_{1,2,3,4}$ can give most coefficients of physical meanings in Eq. (7)

In the zero-temperature limit ($T \to 0$), we can approximate that $\rho _{xxT} \simeq 0$ and $\rho _{xx} \simeq \rho _{xx0} = \sigma _{xx0}^{ - 1}$, then the scaling law becomes $V_y^N/(V_x^L)^2 \simeq {\cal{C}}_1\sigma _{xx0} + {\cal{C}}_2$, which indicates a linear scaling behavior as shown in Fig. 3a. Fitting the experimental data using this relation, the extrinsic skew-scattering coefficient ${\cal{C}}^{sk,2}$ can be experimentally extracted from the total nonlinear Hall conductivity (e.g., by using multi-step samples^20,21,26^). Furthermore, at finite temperatures, it is more convenient to rewrite the scaling law into

10 $$\begin{array}{*{20}{l}} {\frac{{V_y^N}}{{(V_x^L)^2}} - {\cal{C}}_1\sigma _{xx0}^{ - 1}\sigma _{xx}^2} \hfill & \simeq \hfill & {({\cal{C}}_2 + {\cal{C}}_4 - {\cal{C}}_3)\sigma _{xx0}^{ - 2}\sigma _{xx}^2} \hfill \\ {} \hfill & {} \hfill & { + ({\cal{C}}_3 - 2{\cal{C}}_4)\sigma _{xx0}^{ - 1}\sigma _{xx} + {\cal{C}}_4.} \hfill \end{array}$$

In this case, the proper scaling variable becomes $V_y^N/(V_x^L)^2 - {\cal{C}}_1\sigma _{xx0}^{ - 1}\sigma _{xx}^2$, which is a parabolic function of $\sigma _{xx0}^{ - 1}\sigma _{xx}$ thus indicates a scaling behavior shown in Fig. 3b. By fitting the experimental data with the parabolic function, one can in principle extract the information of the rest scaling parameters, as shown in Fig. 3. Equation (10) can be reorganized as $V_y^N/(V_x^L)^2$ − ${\cal{C}}_1\sigma _{xx0}^{ - 1}\sigma _{xx}^2 \simeq ({\cal{C}}_2 - {\cal{C}}_4)\sigma _{xx0}^{ - 2}\sigma _{xx}^2$ + $({\cal{C}}_3 - 2{\cal{C}}_4)(\sigma _{xx0}^{ - 1}\sigma _{xx} - \sigma _{xx0}^{ - 2}\sigma _{xx}^2) + {\cal{C}}_4$. In the anomalous Hall effect, the second term on the right has been argued to be negligible in both the high-temperature limit ($\sigma _{xx0} \gg \sigma _{xx}$) and the low-temperature limit ($\sigma _{xx0} \simeq \sigma _{xx}$)^20^. This linear scaling behavior has been observed in thin films of WTe_2_^11^. Nevertheless, Eq. (10) shows that the linear scaling behavior with $\sigma _{xx}^2$ may become invalid in the high-conductivity regime^21,26^. In the nonmagnetic Weyl–Kondo semimetal Ce_3_Bi_4_Pd_3_, a linear scaling behavior of $\sigma _{xy}^N$ is observed as a function of *σ*_*xx*_^25^. The scaling law of voltages in Eq. (10) can be written into the scaling law of the nonlinear Hall conductivity

11 $$\begin{array}{*{20}{l}} {\sigma _{xy}^N} \hfill & \simeq \hfill & {{\cal{C}}_1\sigma _{xx0}^{ - 1}\sigma _{xx}^3 + ({\cal{C}}_2 - {\cal{C}}_3 + {\cal{C}}_4)\sigma _{xx0}^{ - 2}\sigma _{xx}^3} \hfill \\ {} \hfill & {} \hfill & { + ({\cal{C}}_3 - 2{\cal{C}}_4)\sigma _{xx0}^{ - 1}\sigma _{xx}^2 + {\cal{C}}_4\sigma _{xx},} \hfill \end{array}$$

for a fixed electric field. According to the conductivity scaling law, the observed linear behavior^25^ indicates the dominance of the scaling parameter ${\cal{C}}_4$. According to Eq. (9), ${\cal{C}}_4$ is contributed mainly by the intrinsic mechanism and the dynamical scattering processes (e.g., Fig. 2b, c).

## Methods

### Boltzmann formalism in the nonlinear regime

In the Boltzmann formalism, the distribution function *f*_*l*_ can be found from the standard Boltzmann equation^27^, which reads

12 $$\frac{{\partial f_l}}{{\partial t}} + {\dot{\mathbf{k}}} \cdot \frac{{\partial f_l}}{{\partial {\mathbf{k}}}} = {\cal{I}}_{el}\{ f_l\}$$

in the spatially uniform case. Here ${\cal{I}}_{el}\{ f_l\}$ represents the elastic disorder scattering by static defects or impurities. The elastic disorder scattering can be decomposed as the intrinsic, side-jump, and skew-scattering parts (Supplementary Note 1)

13 $${\cal{I}}_{el}\{ f_l\} = {\cal{I}}_{el}^{in}\{ f_l\} + {\cal{I}}_{el}^{sj}\{ f_l\} + {\cal{I}}_{el}^{sk}\{ f_l\} .$$

The intrinsic part is contributed by symmetric scatterings, in which incoming and outgoing states are reversible in a scattering event. The side-jump part is resulting from the coordinates shift during scattering processes. The skew-scattering part is contributed by anti-symmetric scatterings, in which exchanging the incoming and outgoing states yields a minus sign. Specifically, ${\cal{I}}_{el}^{in}\{ f_l\} = - \mathop {\sum}\nolimits_{l\prime } \varpi _{ll\prime }^{sy}(f_l - f_{l\prime })$, ${\cal{I}}_{el}^{sj}\{ f_l\} = - e{\mathbf{E}} \cdot \mathop {\sum}\nolimits_{l\prime } {\mathbf{O}}_{ll\prime }(f_l - f_{l\prime })$, ${\cal{I}}_{el}^{sk}\{ f_l\} = - \mathop {\sum}\nolimits_{l\prime } \varpi _{l\prime l}^{as}(f_l + f_{l\prime })$, where $\varpi _{ll\prime }^{sy}$ and $\varpi _{ll\prime }^{as}$ represent the symmetric and antisymmetric parts of the scattering rate $\varpi _{ll\prime } = (2\pi /\hbar )|T_{ll\prime }|^2\delta (\varepsilon _l - \varepsilon _{l\prime })$ with $T_{ll\prime }$ representing the T-matrix^27^. ${\mathbf{O}}_{ll\prime } \equiv (2\pi /\hbar )|T_{ll\prime }|^2\delta {\mathbf{r}}_{ll\prime }\frac{\partial }{{\partial \varepsilon _l}}\delta (\varepsilon _l - \varepsilon _{l\prime })$, where the coordinates shift $\delta {\mathbf{r}}_{ll\prime }$ is defined in Table 1. The expression of ${\dot{\mathbf{r}}}_l$ and ${\dot{\mathbf{k}}}$ can be found from the semiclassical equations of motion^15,28^

14 $${\dot{\mathbf{r}}}_l = {\mathbf{v}}_l - {\dot{\mathbf{k}}} \times {\mathrm{\Omega }}_l + {\mathbf{v}}_l^{sj},\quad {\dot{\mathbf{k}}} = - \frac{e}{\hbar }{\mathbf{E}},$$

where ${\mathbf{v}}_l = \partial \varepsilon _l/\hbar \partial k$ is the group velocity, ${\mathbf{\Omega }}_l$ is the Berry curvature^15^, and ${\mathbf{v}}_l^{sj}$ is the side-jump velocity^28^ (see Table 1). To solve the Boltzmann equations up to the second order of **E**, we adopt the relaxation time approximation^27^ for the intrinsic scattering parts ${\cal{I}}_{el}^{in}\{ f_l\} = (f_l^{(0)} - f_l)/\tau _l$, where $f_l^{(0)}$ is the Fermi distribution function and $\tau _l$ represents the relaxation time. Usually, in good metal regime, $\tau _l$ is treated as a constant that can be determined by experiments. For systems with large anisotropy, $\tau _l$ can have a significant angular dependence^29,30^. With the above equations, the current up to the second-order responses to the *ac* electric field can be obtained.

### Tilted 2D massive Dirac model with disorder

We use the tilted 2D massive Dirac model in Eq. (2) to calculate the nonlinear Hall conductivity in Fig. 2. The model describes two energy bands (denoted as ±) with the band dispersions $\varepsilon _{\mathbf{k}}^ \pm = tk_x \pm [v^2k^2 + m^2]^{1/2}$, where $k^2 \equiv k_x^2 + k_y^2$. In the *x*–*y* plane, the Berry curvature behaves like a pseudoscalar, with only the *z* component ${\mathrm{\Omega }}_{ \pm {\mathbf{k}}}^z = \mp mv^2/[2(v^2k^2 + m^2)^{3/2}]$.

To consider the disorder effect, we expanded the scattering rate up to the fourth order in the disorder strength as $\varpi _{ll\prime } = \varpi _{ll\prime }^{(2)} + \varpi _{ll\prime }^{(3)} + \varpi _{ll\prime }^{(4)}$. Here $\varpi _{ll\prime }^{(2)}$ is pure symmetric and of order $n_iV_0^2$ with *n*_*i*_ refers to the concentration of disorder. Figure 2a corresponds to the contribution to $\varpi _{ll\prime }^{(3)}$, which is non-Gaussian and of order $n_iV_1^3$. Figure 2b, c corresponds to $\varpi _{ll\prime }^{(4)}$ within non-crossing approximation, which is Gaussian and of order $n_i^2V_0^4$. Thus, $\varpi _{ll\prime }^{(2)}$ is the leading symmetric contribution, $\varpi _{ll\prime }^{(3)}$ and $\varpi _{ll\prime }^{(4)}$ contain the leading non-Gaussian and Gaussian antisymmetric contribution to the scattering rate. Considering all the leading contributions, we identify that $\varpi _{ll\prime }^{sy} = \varpi _{ll\prime }^{(2)}$ and $\varpi _{ll\prime }^{as} = \varpi _{ll\prime }^{(3a)} + \varpi _{ll\prime }^{(4a)}$, where $\varpi _{ll\prime }^{(3a)}$ and $\varpi _{ll\prime }^{(4a)}$ represent the antisymmetric parts of the third and fourth order scattering rate, respectively (Supplementary Note 4).

## Supplementary Information

Supplementary Information

## Data Availability

The data that support the plots within this paper and other findings of this study are available from the corresponding author upon reasonable request.

## Code availability

The code that is deemed central to the conclusions is available from the corresponding author upon reasonable request.
