# Supplementary material for: Disorder-induced nonlinear Hall effect with time-reversal symmetry
Source: Nat Commun. 2019 Jul 11;10:3047. doi: 10.1038/s41467-019-10941-3 (PMC6624286; doi:10.1038/s41467-019-10941-3)
Supplement: Supplementary file 1 — Supplementary Information [file 41467_2019_10941_MOESM1_ESM.pdf]

Supplementary Information for
"Disorder-induced nonlinear Hall effect with time-reversal symmetry"

Z. Z. Du,[1,2,3] C. M. Wang,[4,1,2] Shuai Li,[1,2] Hai-Zhou Lu,[1,2,3,*] and X. C. Xie[5,6,7]

[1]*Shenzhen Institute for Quantum Science and Engineering and Department of Physics, Southern University of Science and Technology, Shenzhen 518055, China*
[2]*Shenzhen Key Laboratory of Quantum Science and Engineering, Shenzhen 518055, China*
[3]*Peng Cheng Laboratory, Shenzhen 518055, China*
[4]*Department of Physics, Shanghai Normal University, Shanghai 200234, China*
[5]*International Center for Quantum Materials, School of Physics, Peking University, Beijing 100871, China*
[6]*Beijing Academy of Quantum Information Sciences, Haidian District, Beijing 100193, China*
[7]*CAS Center for Excellence in Topological Quantum Computation, University of Chinese Academy of Sciences, Beijing 100190, China*

(Dated: June 10, 2019)

CONTENTS

Supplementary Note 1 — 3

Supplementary Note 2 — 4
- Intrinsic contribution — 5
- Side-jump contribution — 6
- Skew-scattering contribution — 8

Supplementary Note 3 — 11
- Anomalous Hall effect — 11
  - Frequency-dependence in the isotropic limit — 11
  - Anomalous Hall conductivity in the low-frequency limit — 12
- Nonlinear Hall effect in $\mathcal{T}$-broken systems — 12
  - Frequency-dependence in the isotropic limit — 13
  - Nonlinear Hall conductivity in the low-frequency limit — 14
- Nonlinear Hall effect in $\mathcal{T}$-symmetric systems — 14
  - Frequency-dependence in the isotropic limit — 15
  - Nonlinear Hall conductivity in the low-frequency limit — 16
- Disorder-dependence and mixed contributions — 16

Supplementary Note 4 — 17
- Scattering rate — 17
  - Second-order scattering rate — 18
  - Third-order scattering rate — 19
  - Fourth-order scattering rate — 19
- Coordinate shift — 22
- Anomalous Hall conductivity — 23
  - Intrinsic contribution — 24
  - Side-jump contribution — 24
  - Skew-scattering contribution — 25
- Nonlinear Hall conductivity — 26
  - Intrinsic contribution — 27
  - Side-jump contribution — 27
  - Skew-scattering contribution — 28

Supplementary Note 5 — 28
- Scaling law of anomalous Hall effect — 28
- Scaling law of nonlinear Hall effect — 29

Supplementary Methods 30
Calculation of the side-jump related quantities 30
Calculation of the skew-scattering related quantities 32

Supplementary References 34

SUPPLEMENTARY NOTE 1

Boltzmann formalism: In the space uniform case, the standard Boltzmann equation reads [1]

$$\frac{\partial f_l}{\partial t} + \dot{\mathbf{k}} \cdot \frac{\partial f_l}{\partial \mathbf{k}} = \mathcal{I}_{el}\{f_l\}. \tag{1}$$

Here $\mathcal{I}_{el}\{f_l\}$ represents the elastic scattering by static defects or disorders with a general distribution function f_l and $l = (\eta, \mathbf{k})$ is a combined index with $\mathbf{k}$ the momentum index and η the label of band. The collision term can be formally written as [2, 3]

$$\mathcal{I}_{el}\{f_l\} = -\sum_{l'} (\varpi_{l'l} f_l - \varpi_{ll'} f_{l'}), \tag{2}$$

where $\sum_{l'} \equiv \sum_{\eta'} \int [dk']$ with $[dk'] \equiv d^d k'/(2\pi)^d$. The scattering rate $\varpi_{ll'}$ between l and l' states is related to the T-matrix elements according to the so called golden rule

$$\varpi_{ll'} \equiv \frac{2\pi}{\hbar} |T_{ll'}|^2 \delta(\varepsilon_l - \varepsilon_{l'}). \tag{3}$$

In the general case, the scattering rate is not symmetric with respect to the interchange of the initial and final states, $\varpi_{ll'} \neq \varpi_{l'l}$. Therefore, $\varpi_{ll'}$ can be formally decomposed into the symmetric and antisymmetric parts as

$$\varpi_{ll'} \equiv \varpi^{sy}_{ll'} + \varpi^{as}_{ll'}, \qquad \varpi^{sy}_{ll'} = \varpi^{sy}_{l'l}, \qquad \varpi^{as}_{ll'} = -\varpi^{as}_{l'l}. \tag{4}$$

Accordingly, the collision term can also be decomposed as

$$\mathcal{I}_{el}\{f_l\} = \mathcal{I}^{sy}_{el}\{f_l\} + \mathcal{I}^{sk}_{el}\{f_l\}, \tag{5}$$

where

$$\mathcal{I}^{sy}_{el}\{f_l\} = -\sum_{l'} \varpi^{sy}_{ll'} (f_l - f_{l'}), \tag{6}$$

$$\mathcal{I}^{sk}_{el}\{f_l\} = -\sum_{l'} \varpi^{as}_{l'l} (f_l + f_{l'}). \tag{7}$$

The antisymmetric part $\mathcal{I}^{sk}_{el}\{f_l\}$ is responsible for the skew-scattering contribution to the anomalous and nonlinear Hall effects.

If we take into account the work done by the electric field as an electron gets displaced within the unit cell during the collision, the scattering rate is modified as

$$\varpi^{sy}_{ll'} \;\Rightarrow\; \tilde{\varpi}^{sy}_{ll'} \equiv \frac{2\pi}{\hbar} |T_{ll'}|^2 \delta(\varepsilon_l - \varepsilon_{l'} + e\mathbf{E} \cdot \delta\mathbf{r}_{ll'}). \tag{8}$$

Here we only consider the modification to the symmetric part, that is, we've neglected the mixed contribution from the skew-scattering and the coordinate shift, which can be neglected in the weak disorder limit (see Sec.). And the coordinate shift, usually referred to as side-jump, is given for a weak impurity potential $\hat{V}_{imp}$ by [3]

$$\delta\mathbf{r}_{ll'} = \left\langle u_l \middle| i\frac{\partial u_l}{\partial \mathbf{k}} \right\rangle - \left\langle u_{l'} \middle| i\frac{\partial u_{l'}}{\partial \mathbf{k}'} \right\rangle - \hat{\mathbf{D}}_{\mathbf{k}\mathbf{k}'} \arg(V_{ll'}) \tag{9}$$

with

$$\hat{\mathbf{D}}_{\mathbf{k}\mathbf{k}'} \equiv \frac{\partial}{\partial \mathbf{k}} + \frac{\partial}{\partial \mathbf{k}'}, \qquad V_{ll'} \equiv \langle u_l | \hat{V}_{imp} | u_{l'} \rangle. \tag{10}$$

Below we take into account contributions up to the first order in $\delta\mathbf{r}_{ll'}$, which allows one to approximate the δ-function as

$$\begin{aligned}\delta[\varepsilon_l - \varepsilon_{l'} + e\mathbf{E} \cdot \delta\mathbf{r}_{ll'}] &\simeq \delta(\varepsilon_l - \varepsilon_{l'}) + e\mathbf{E} \cdot \delta\mathbf{r}_{ll'} \frac{\partial}{\partial \varepsilon_l} \delta(\varepsilon_l - \varepsilon_{l'}) \\ &= \delta(\varepsilon_l - \varepsilon_{l'}) - e\mathbf{E} \cdot \delta\mathbf{r}_{ll'} \frac{\partial}{\partial \varepsilon_{l'}} \delta(\varepsilon_l - \varepsilon_{l'}).\end{aligned} \tag{11}$$

Thus the symmetric collision term can be written as

$$\begin{aligned}\mathcal{I}_{el}^{sy}\{f_l\} &= -\sum_{l'}\tilde{\varpi}_{ll'}^{sy}(f_l - f_{l'}) \\ &\simeq -\sum_{l'}(\varpi_{ll'}^{sy} + e\mathbf{E}\cdot\mathbf{O}_{ll'})(f_l - f_{l'}) \\ &\equiv \mathcal{I}_{el}^{in}\{f_l\} + \mathcal{I}_{el}^{sj}\{f_l\}\end{aligned} \tag{12}$$

with

$$\mathcal{I}_{el}^{in}\{f_l\} = -\sum_{l'}\varpi_{ll'}^{sy}(f_l - f_{l'}), \tag{13}$$

$$\mathcal{I}_{el}^{sj}\{f_l\} = -e\mathbf{E}\cdot\sum_{l'}\mathbf{O}_{ll'}(f_l - f_{l'}), \tag{14}$$

where we have defined

$$\mathbf{O}_{ll'} \equiv \frac{2\pi}{\hbar}|T_{ll'}|^2\delta\mathbf{r}_{ll'}\frac{\partial}{\partial\varepsilon_l}\delta(\varepsilon_l - \varepsilon_{l'}). \tag{15}$$

In summary, the elastic collision term has been approximately decomposed into the intrinsic, side-jump and skew-scattering parts as

$$\mathcal{I}_{el}\{f_l\} = \mathcal{I}_{el}^{in}\{f_l\} + \mathcal{I}_{el}^{sj}\{f_l\} + \mathcal{I}_{el}^{sk}\{f_l\}. \tag{16}$$

To solve the Boltzmann equation, we formally decompose the distribution function accordingly as

$$f_l = f_l^{in} + \delta f_l^{sj} + \delta f_l^{sk}. \tag{17}$$

And the standard Boltzmann equation then becomes

$$(\partial_t + \dot{\mathbf{k}}\cdot\vec{\partial}_{\mathbf{k}})\cdot(f_l^{in} + \delta f_l^{sj} + \delta f_l^{sk}) = \mathcal{I}_{el}^{in}\{f_l\} + \mathcal{I}_{el}^{sj}\{f_l\} + \mathcal{I}_{el}^{sk}\{f_l\}. \tag{18}$$

Approximately, we can decomposed the standard Boltzmann equation into three equations as

$$(\partial_t + \dot{\mathbf{k}}\cdot\vec{\partial}_{\mathbf{k}})f_l^{in} = \mathcal{I}_{el}^{in}\{f_l^{in}\}, \tag{19}$$

$$(\partial_t + \dot{\mathbf{k}}\cdot\vec{\partial}_{\mathbf{k}})\delta f_l^{sj} = \mathcal{I}_{el}^{in}\{\delta f_l^{sj}\} + \mathcal{I}_{el}^{sj}\{f_l^{in}\}, \tag{20}$$

$$(\partial_t + \dot{\mathbf{k}}\cdot\vec{\partial}_{\mathbf{k}})\delta f_l^{sk} = \mathcal{I}_{el}^{in}\{\delta f_l^{sk}\} + \mathcal{I}_{el}^{sk}\{f_l^{in}\}, \tag{21}$$

where we've neglected the terms with mixed side-jump and skew-scattering contributions.

SUPPLEMENTARY NOTE 2

Response currents in absence of magnetic field: In the absence of the magnetic field, the semiclassical equations of motion are [3, 4]

$$\dot{\mathbf{r}}_l = \mathbf{v}_l - \dot{\mathbf{k}}\times\mathbf{\Omega}_l + \mathbf{v}_l^{sj}, \tag{22}$$

$$\dot{\mathbf{k}} = -\frac{e}{\hbar}\mathbf{E}, \tag{23}$$

where $\mathbf{v}_l$ and $\mathbf{v}_l^{sj}$ are the intrinsic and side-jump velocities

$$\mathbf{v}_l = \frac{1}{\hbar}\frac{\partial\varepsilon_l}{\partial\mathbf{k}}, \qquad \mathbf{v}_l^{sj} = \sum_{l'}\varpi_{ll'}^{sy}\delta\mathbf{r}_{l'l}. \tag{24}$$

Combining these two equations, we have

$$\dot{\mathbf{r}} = \mathbf{v}_l + \frac{e}{\hbar}\mathbf{E}\times\mathbf{\Omega}_l + \mathbf{v}_l^{sj}, \tag{25}$$

and the electric current reads

$$\mathbf{J}(\mathbf{E}) = -e\sum_l\dot{\mathbf{r}}_l f_l = -e\sum_l\Big(\mathbf{v}_l + \frac{e}{\hbar}\mathbf{E}\times\mathbf{\Omega}_l + \mathbf{v}_l^{sj}\Big)f_l. \tag{26}$$

Assume an ac electric field $\mathbf{E}(t) = \mathrm{Re}\{\mathcal{E}e^{i\omega t}\}$ with the amplitude vector $\mathcal{E}$ and frequency ω, the decomposed Boltzmann equations become

$$\big[\partial_t - \frac{e}{2\hbar}\big(\mathcal{E}_a e^{i\omega t} + \mathcal{E}_a^* e^{-i\omega t}\big)\partial_{\mathbf{k}}^a\big] f_l^{in} = \mathcal{I}_{el}^{in}\{f_l^{in}\}, \tag{27}$$

$$\big[\partial_t - \frac{e}{2\hbar}\big(\mathcal{E}_a e^{i\omega t} + \mathcal{E}_a^* e^{-i\omega t}\big)\partial_{\mathbf{k}}^a\big] \delta f_l^{sj} = \mathcal{I}_{el}^{in}\{\delta f_l^{sj}\} + \mathcal{I}_{el}^{sj}\{f_l^{in}\}, \tag{28}$$

$$\big[\partial_t - \frac{e}{2\hbar}\big(\mathcal{E}_a e^{i\omega t} + \mathcal{E}_a^* e^{-i\omega t}\big)\partial_{\mathbf{k}}^a\big] \delta f_l^{sk} = \mathcal{I}_{el}^{in}\{\delta f_l^{sk}\} + \mathcal{I}_{el}^{sk}\{f_l^{in}\}. \tag{29}$$

Intrinsic contribution

Note that the first equation only contains the symmetric scattering, thus can be solved within the relaxation time approximation

$$\frac{\partial f_l^{in}}{\partial t} - \frac{e}{2\hbar}\big(\mathcal{E}e^{i\omega t} + \mathcal{E}^* e^{-i\omega t}\big)\frac{\partial f_l^{in}}{\partial \mathbf{k}} = \frac{f_l^{(0)} - f_l^{in}}{\tau_l}$$
$$\Rightarrow \quad f_l^{in} = \frac{f_l^{(0)}}{1 + \tau_l \partial_t - (e\tau_l/2\hbar)\big(\mathcal{E}_a e^{i\omega t} + \mathcal{E}_a^* e^{-i\omega t}\big)\partial_{\mathbf{k}}^a}, \tag{30}$$

where $f_l^{(0)}$ refers to the Fermi-Dirac distribution function and τ_l refers to a general relaxation time. We are interested in computing the response up to second-order in the electric field, hence, we only expand the distribution function up to second-order as

$$f_l^{in} = \sum_{n=0}^{\infty}\Big[-\tau_l\partial_t + \frac{e\tau_l}{2\hbar}\big(\mathcal{E}_a e^{i\omega t} + \mathcal{E}_a^* e^{-i\omega t}\big)\partial_{\mathbf{k}}^a\Big]^n f_l^{(0)} \equiv f_l^{(0)} + \delta^1 f_l^{in} + \delta^2 f_l^{in} + \cdots, \tag{31}$$

where $\delta^1 f_l^{in}$ refers to the linear dependent part

$$\begin{aligned}\delta^1 f_l^{in} &= \frac{e\tau_l}{2\hbar}\sum_{n=0}^{\infty}\big(-\tau_l\partial_t\big)^n\big(\mathcal{E}_a e^{i\omega t} + \mathcal{E}_a^* e^{-i\omega t}\big)\partial_{\mathbf{k}}^a f_l^{(0)} \\ &= \frac{e\tau_l}{2\hbar}\Big(\frac{\mathcal{E}_a e^{i\omega t}}{1+i\omega\tau_l} + \frac{\mathcal{E}_a^* e^{-i\omega t}}{1-i\omega\tau_l}\Big)\partial_{\mathbf{k}}^a f_l^{(0)},\end{aligned} \tag{32}$$

and $\delta^2 f_l^{in}$ represents the second-order part

$$\begin{aligned}\delta^2 f_l^{in} &= \frac{e\tau_l}{2\hbar}\sum_{m,n=0}^{\infty}\big(-\tau_l\partial_t\big)^m\Big[\big(\mathcal{E}_a e^{i\omega t} + \mathcal{E}_a^* e^{-i\omega t}\big)\partial_{\mathbf{k}}^a\Big]\big(-\tau_l\partial_t\big)^n\frac{e\tau_l}{2\hbar}\big(\mathcal{E}_b e^{i\omega t} + \mathcal{E}_b^* e^{-i\omega t}\big)\partial_{\mathbf{k}}^b f_l^{(0)} \\ &= \frac{e^2\tau_l}{4\hbar^2}\Big\{\tau_l\Big(\frac{\mathcal{E}_a^*\mathcal{E}_b}{1+i\omega\tau_l} + \frac{\mathcal{E}_b^*\mathcal{E}_a}{1-i\omega\tau_l}\Big)\partial_{\mathbf{k}}^a\partial_{\mathbf{k}}^b f_l^{(0)} + \Big[\frac{\mathcal{E}_a^*\mathcal{E}_b}{(1+i\omega\tau_l)^2} + \frac{\mathcal{E}_b^*\mathcal{E}_a}{(1-i\omega\tau_l)^2}\Big]\big(\partial_{\mathbf{k}}^a\tau_l\big)\partial_{\mathbf{k}}^b f_l^{(0)} \\ &+\tau_l\Big[\frac{\mathcal{E}_a\mathcal{E}_b e^{i2\omega t}}{(1+i\omega\tau_l)(1+i2\omega\tau_l)} + \frac{\mathcal{E}_a^*\mathcal{E}_b^* e^{-i2\omega t}}{(1-i\omega\tau_l)(1-i2\omega\tau_l)}\Big]\partial_{\mathbf{k}}^a\partial_{\mathbf{k}}^b f_l^{(0)} \\ &+\Big[\frac{\mathcal{E}_a\mathcal{E}_b e^{i2\omega t}}{(1+i\omega\tau_l)^2(1+i2\omega\tau_l)} + \frac{\mathcal{E}_a^*\mathcal{E}_b^* e^{-i2\omega t}}{(1-i\omega\tau_l)^2(1-i2\omega\tau_l)}\Big]\big(\partial_{\mathbf{k}}^a\tau_l\big)\partial_{\mathbf{k}}^b f_l^{(0)}\Big\}.\end{aligned} \tag{33}$$

And the corresponding electric current can be obtained as

$$\begin{aligned}
J_a^{in}(\mathbf{E}) &= -e\sum_l \Big[v_l^a + \frac{e}{2\hbar}\varepsilon^{ade}\big(\mathcal{E}_d e^{i\omega t} + \mathcal{E}_d^* e^{-i\omega t}\big)\Omega_l^e\Big]\Big(f_l^{(0)} + \delta^1 f_l^{in} + \delta^2 f_l^{in}\Big) \\
&= -e\sum_l \Big[v_l^a + \frac{e}{2\hbar}\varepsilon^{ade}\big(\mathcal{E}_d e^{i\omega t} + \mathcal{E}_d^* e^{-i\omega t}\big)\Omega_l^e\Big]\Bigg\{f_l^{(0)} + \frac{e\tau_l}{2\hbar}\Big(\frac{\mathcal{E}_b e^{i\omega t}}{1+i\omega\tau_l} + \frac{\mathcal{E}_b^* e^{-i\omega t}}{1-i\omega\tau_l}\Big)\partial_{\mathbf{k}}^b f_l^{(0)} \\
&+ \frac{e^2\tau_l}{4\hbar^2}\Big\{\tau_l\Big(\frac{\mathcal{E}_b^*\mathcal{E}_c}{1+i\omega\tau_l} + \frac{\mathcal{E}_c^*\mathcal{E}_b}{1-i\omega\tau_l}\Big)\partial_{\mathbf{k}}^b\partial_{\mathbf{k}}^c f_l^{(0)} + \Big[\frac{\mathcal{E}_b^*\mathcal{E}_c}{(1+i\omega\tau_l)^2} + \frac{\mathcal{E}_c^*\mathcal{E}_b}{(1-i\omega\tau_l)^2}\Big]\big(\partial_{\mathbf{k}}^b\tau_l\big)\partial_{\mathbf{k}}^c f_l^{(0)} \\
&+ \tau_l\Big[\frac{\mathcal{E}_b\mathcal{E}_c e^{i2\omega t}}{(1+i\omega\tau_l)(1+i2\omega\tau_l)} + \frac{\mathcal{E}_b^*\mathcal{E}_c^* e^{-i2\omega t}}{(1-i\omega\tau_l)(1-i2\omega\tau_l)}\Big]\partial_{\mathbf{k}}^b\partial_{\mathbf{k}}^c f_l^{(0)} \\
&+ \Big[\frac{\mathcal{E}_b\mathcal{E}_c e^{i2\omega t}}{(1+i\omega\tau_l)^2(1+i2\omega\tau_l)} + \frac{\mathcal{E}_b^*\mathcal{E}_c^* e^{-i2\omega t}}{(1-i\omega\tau_l)^2(1-i2\omega\tau_l)}\Big]\big(\partial_{\mathbf{k}}^b\tau_l\big)\partial_{\mathbf{k}}^c f_l^{(0)}\Big\}\Bigg\} \\
&\equiv \mathrm{Re}\big\{J_a^{in}(0) + J_a^{in}(\omega)e^{i\omega t} + J_a^{in}(2\omega)e^{i2\omega t}\big\}
\end{aligned} \tag{34}$$

with

$$J_a^{in}(0) = -\frac{e^3\mathcal{E}_b\mathcal{E}_c^*}{2\hbar^2}\sum_l \frac{\tau_l}{1+i\omega\tau_l}\Big(\varepsilon^{acd}\Omega_l^d\partial_{\mathbf{k}}^b + \tau_l v_l^a\partial_{\mathbf{k}}^c\partial_{\mathbf{k}}^b + \frac{v_l^a\partial_{\mathbf{k}}^c\tau_l}{1+i\omega\tau_l}\partial_{\mathbf{k}}^b\Big)f_l^{(0)}, \tag{35}$$

$$J_a^{in}(\omega) = -\frac{e^2\mathcal{E}_b}{\hbar}\sum_l \Big(\varepsilon^{abc}\Omega_l^c + \frac{\tau_l v_l^a}{1+i\omega\tau_l}\partial_{\mathbf{k}}^b\Big)f_l^{(0)}, \tag{36}$$

$$J_a^{in}(2\omega) = -\frac{e^3\mathcal{E}_b\mathcal{E}_c}{2\hbar^2}\sum_l \frac{\tau_l}{1+i\omega\tau_l}\Big[\varepsilon^{acd}\Omega_l^d\partial_{\mathbf{k}}^b + \frac{\tau_l v_l^a}{1+i2\omega\tau_l}\partial_{\mathbf{k}}^c\partial_{\mathbf{k}}^b + \frac{v_l^a\partial_{\mathbf{k}}^c\tau_l}{(1+i\omega\tau_l)(1+i2\omega\tau_l)}\partial_{\mathbf{k}}^b\Big]f_l^{(0)}. \tag{37}$$

Side-jump contribution

The side-jump induced modification to the distribution function can be obtained by putting the expression of f_l^{in} into Eq.(28) as

$$\big[\partial_t - \frac{e}{2\hbar}\big(\mathcal{E}_a e^{i\omega t} + \mathcal{E}_a^* e^{-i\omega t}\big)\partial_{\mathbf{k}}^a\big]\delta f_l^{sj} = -\frac{\delta f_l^{sj}}{\tau_l} - \frac{e}{2}\big(\mathcal{E}_b e^{i\omega t} + \mathcal{E}_b^* e^{-i\omega t}\big)\sum_{l'} O_{ll'}^b(f_l^{in} - f_{l'}^{in}), \tag{38}$$

where we also used relaxation time approximation for the intrinsic collision part. The above equation can be formally solved as

$$\begin{aligned}
\delta f_l^{sj} &= -\frac{(e\tau_l/2)\big(\mathcal{E}_b e^{i\omega t} + \mathcal{E}_b^* e^{-i\omega t}\big)\sum_{l'} O_{ll'}^b(f_l^{in} - f_{l'}^{in})}{1 + \tau_l\partial_t - (e\tau_l/2\hbar)\big(\mathcal{E}_a e^{i\omega t} + \mathcal{E}_a^* e^{-i\omega t}\big)\partial_{\mathbf{k}}^a} \\
&= -\frac{e}{2}\sum_{n=0}^{\infty}\Big[-\tau_l\partial_t + \frac{e\tau_l}{2\hbar}\big(\mathcal{E}_a e^{i\omega t} + \mathcal{E}_a^* e^{-i\omega t}\big)\partial_{\mathbf{k}}^a\Big]^n \tau_l\big(\mathcal{E}_b e^{i\omega t} + \mathcal{E}_b^* e^{-i\omega t}\big)\sum_{l'} O_{ll'}^b(f_l^{in} - f_{l'}^{in}) \\
&= -\frac{e}{2}\sum_{n=0}^{\infty}\Big[-\tau_l\partial_t + \frac{e\tau_l}{2\hbar}\big(\mathcal{E}_a e^{i\omega t} + \mathcal{E}_a^* e^{-i\omega t}\big)\partial_{\mathbf{k}}^a\Big]^n \tau_l\big(\mathcal{E}_b e^{i\omega t} + \mathcal{E}_b^* e^{-i\omega t}\big)\sum_{l'} O_{ll'}^b\Big[f_l^{(0)} - f_{l'}^{(0)} \\
&+ \frac{e\tau_l}{2\hbar}\Big(\frac{\mathcal{E}_c e^{i\omega t}}{1+i\omega\tau_l} + \frac{\mathcal{E}_c^* e^{-i\omega t}}{1-i\omega\tau_l}\Big)\partial_{\mathbf{k}}^c f_l^{(0)} - \frac{e\tau_{l'}}{2\hbar}\Big(\frac{\mathcal{E}_c e^{i\omega t}}{1+i\omega\tau_{l'}} + \frac{\mathcal{E}_c^* e^{-i\omega t}}{1-i\omega\tau_{l'}}\Big)\partial_{\mathbf{k}'}^c f_{l'}^{(0)}\Big] \\
&\equiv \delta^1 f_l^{sj} + \delta^2 f_l^{sj}.
\end{aligned} \tag{39}$$

The linear dependent part reads

$$\begin{aligned}
\delta^1 f_l^{sj} &= -\frac{e}{2}\sum_{n=0}^{\infty}(-\tau_l\partial_t)^n\tau_l\big(\mathcal{E}_a e^{i\omega t} + \mathcal{E}_a^* e^{-i\omega t}\big)Q_l^a \\
&= -\frac{e\tau_l}{2}\Big(\frac{\mathcal{E}_a e^{i\omega t}}{1+i\omega\tau_l} + \frac{\mathcal{E}_a^* e^{-i\omega t}}{1-i\omega\tau_l}\Big)Q_l^a
\end{aligned} \tag{40}$$

with

$$Q_l^a = \sum_{l'} O_{ll'}^a [f_l^{(0)} - f_{l'}^{(0)}], \tag{41}$$

and the second-order part is

$$\begin{aligned}
\delta^2 f_l^{sj} &= -\frac{e^2\tau_l}{4\hbar}\sum_{m,n=0}^{\infty}\big(-\tau_l\partial_t\big)^m\Big[\big(\mathcal{E}_a e^{i\omega t}+\mathcal{E}_a^* e^{-i\omega t}\big)\partial_{\mathbf{k}}^a\Big]\big(-\tau_l\partial_t\big)^n\tau_l\big(\mathcal{E}_b e^{i\omega t}+\mathcal{E}_b^* e^{-i\omega t}\big)Q_l^b \\
&\quad -\frac{e}{2}\sum_{n=0}^{\infty}(-\tau_l\partial_t)^n\tau_l\big(\mathcal{E}_a e^{i\omega t}+\mathcal{E}_a^* e^{-i\omega t}\big)\frac{e}{2\hbar}\Big[\mathcal{E}_b e^{i\omega t}P_l^{ab}+\mathcal{E}_b^* e^{-i\omega t}(P_l^{ab})^* - \mathcal{E}_b e^{i\omega t}\bar{P}_l^{ab} - \mathcal{E}_b^* e^{-i\omega t}(\bar{P}_l^{ab})^*\Big] \\
&= -\frac{e^2\tau_l}{4\hbar}\Big\{\tau_l\Big(\frac{\mathcal{E}_a^*\mathcal{E}_b}{1+i\omega\tau_l}+\frac{\mathcal{E}_b^*\mathcal{E}_a}{1-i\omega\tau_l}\Big)\partial_{\mathbf{k}}^a Q_l^b + \Big[\frac{\mathcal{E}_a^*\mathcal{E}_b}{(1+i\omega\tau_l)^2}+\frac{\mathcal{E}_b^*\mathcal{E}_a}{(1-i\omega\tau_l)^2}\Big](\partial_{\mathbf{k}}^a\tau_l)Q_l^b \\
&\quad +\mathcal{E}_a^*\mathcal{E}_b\big(P_l^{ab}-\bar{P}_l^{ab}\big)+\mathcal{E}_b^*\mathcal{E}_a\big(P_l^{ab}-\bar{P}_l^{ab}\big)^* + \tau_l\Big[\frac{\mathcal{E}_a\mathcal{E}_b e^{i2\omega t}}{(1+i\omega\tau_l)(1+i2\omega\tau_l)}+\frac{\mathcal{E}_a^*\mathcal{E}_b^* e^{-i2\omega t}}{(1-i\omega\tau_l)(1-i2\omega\tau_l)}\Big]\partial_{\mathbf{k}}^a Q_l^b \\
&\quad +\Big[\frac{\mathcal{E}_a\mathcal{E}_b e^{i2\omega t}}{(1+i\omega\tau_l)^2(1+i2\omega\tau_l)}+\frac{\mathcal{E}_a^*\mathcal{E}_b^* e^{-i2\omega t}}{(1-i\omega\tau_l)^2(1-i2\omega\tau_l)}\Big](\partial_{\mathbf{k}}^a\tau_l)Q_l^b \\
&\quad +\frac{\mathcal{E}_a\mathcal{E}_b e^{i2\omega t}}{1+i2\omega\tau_l}\big(P_l^{ab}-\bar{P}_l^{ab}\big)+\frac{\mathcal{E}_a^*\mathcal{E}_b^* e^{-i2\omega t}}{1-i2\omega\tau_l}\big(P_l^{ab}-\bar{P}_l^{ab}\big)^*\Big\}
\end{aligned} \tag{42}$$

with

$$P_l^{ab} = \sum_{l'} O_{ll'}^a \frac{\tau_l\partial_{\mathbf{k}}^b f_l^{(0)}}{1+i\omega\tau_l}, \qquad \bar{P}_l^{ab} = \sum_{l'} O_{ll'}^a \frac{\tau_{l'}\partial_{\mathbf{k}'}^b f_{l'}^{(0)}}{1+i\omega\tau_{l'}}. \tag{43}$$

There are two contributions to the side-jump contribution. The first comes from the side-jump velocity, which can be obtained as

$$\begin{aligned}
J_a^{sj,1}(\mathbf{E}) &= -e\sum_l v_a^{sj}\Big(\delta^1 f_l^{in}+\delta^2 f_l^{in}\Big) \\
&= -e\sum_l v_a^{sj}\Bigg\{\frac{e\tau_l}{2\hbar}\Big(\frac{\mathcal{E}_b e^{i\omega t}}{1+i\omega\tau_l}+\frac{\mathcal{E}_b^* e^{-i\omega t}}{1-i\omega\tau_l}\Big)\partial_{\mathbf{k}}^b f_l^{(0)} \\
&\quad +\frac{e^2\tau_l}{4\hbar^2}\Big\{\tau_l\Big(\frac{\mathcal{E}_b^*\mathcal{E}_c}{1+i\omega\tau_l}+\frac{\mathcal{E}_c^*\mathcal{E}_b}{1-i\omega\tau_l}\Big)\partial_{\mathbf{k}}^b\partial_{\mathbf{k}}^c f_l^{(0)}+\Big[\frac{\mathcal{E}_b^*\mathcal{E}_c}{(1+i\omega\tau_l)^2}+\frac{\mathcal{E}_c^*\mathcal{E}_b}{(1-i\omega\tau_l)^2}\Big]\big(\partial_{\mathbf{k}}^b\tau_l\big)\partial_{\mathbf{k}}^c f_l^{(0)} \\
&\quad +\tau_l\Big[\frac{\mathcal{E}_b\mathcal{E}_c e^{i2\omega t}}{(1+i\omega\tau_l)(1+i2\omega\tau_l)}+\frac{\mathcal{E}_b^*\mathcal{E}_c^* e^{-i2\omega t}}{(1-i\omega\tau_l)(1-i2\omega\tau_l)}\Big]\partial_{\mathbf{k}}^b\partial_{\mathbf{k}}^c f_l^{(0)} \\
&\quad +\Big[\frac{\mathcal{E}_b\mathcal{E}_c e^{i2\omega t}}{(1+i\omega\tau_l)^2(1+i2\omega\tau_l)}+\frac{\mathcal{E}_b^*\mathcal{E}_c^* e^{-i2\omega t}}{(1-i\omega\tau_l)^2(1-i2\omega\tau_l)}\Big]\big(\partial_{\mathbf{k}}^b\tau_l\big)\partial_{\mathbf{k}}^c f_l^{(0)}\Big\}\Bigg\} \\
&\equiv \mathrm{Re}\big\{J_a^{sj,1}(0)+J_a^{sj,1}(\omega)e^{i\omega t}+J_a^{sj,1}(2\omega)e^{i2\omega t}\big\}
\end{aligned} \tag{44}$$

with

$$J_a^{sj,1}(0) = -\frac{e^3\mathcal{E}_b\mathcal{E}_c^*}{2\hbar^2}\sum_l \frac{\tau_l v_a^{sj}}{1+i\omega\tau_l}\Big(\tau_l\partial_{\mathbf{k}}^c\partial_{\mathbf{k}}^b+\frac{\partial_{\mathbf{k}}^c\tau_l}{1+i\omega\tau_l}\partial_{\mathbf{k}}^b\Big)f_l^{(0)}, \tag{45}$$

$$J_a^{sj,1}(\omega) = -\frac{e^2\mathcal{E}_b}{\hbar}\sum_l \frac{\tau_l v_a^{sj}}{1+i\omega\tau_l}\partial_{\mathbf{k}}^b f_l^{(0)}, \tag{46}$$

$$J_a^{sj,1}(2\omega) = -\frac{e^3\mathcal{E}_b\mathcal{E}_c}{2\hbar^2}\sum_l \frac{\tau_l v_a^{sj}}{(1+i\omega\tau_l)(1+i2\omega\tau_l)}\Big(\tau_l\partial_{\mathbf{k}}^c\partial_{\mathbf{k}}^b+\frac{\partial_{\mathbf{k}}^c\tau_l}{1+i\omega\tau_l}\partial_{\mathbf{k}}^b\Big)f_l^{(0)}. \tag{47}$$

And the second contribution comes from the side-jump induced modification to the distribution function, which can

be obtained as

$$
\begin{aligned}
J_a^{sj,2}(\mathbf{E}) &= -e\sum_l \Big[v_l^a + \frac{e}{2\hbar}\varepsilon^{ade}\big(\mathcal{E}_d e^{i\omega t} + \mathcal{E}_d^* e^{-i\omega t}\big)\Omega_l^e\Big]\Big(\delta^1 f_l^{sj} + \delta^2 f_l^{sj}\Big) \\
&= e\sum_l \Big[v_l^a + \frac{e}{2\hbar}\varepsilon^{ade}\big(\mathcal{E}_d e^{i\omega t} + \mathcal{E}_d^* e^{-i\omega t}\big)\Omega_l^e\Big]\Bigg\{\frac{e\tau_l}{2}\Big(\frac{\mathcal{E}_b e^{i\omega t}}{1+i\omega\tau_l} + \frac{\mathcal{E}_b^* e^{-i\omega t}}{1-i\omega\tau_l}\Big)Q_l^b \\
&\quad + \frac{e^2\tau_l}{4\hbar}\Big\{\tau_l\Big(\frac{\mathcal{E}_b^*\mathcal{E}_c}{1+i\omega\tau_l} + \frac{\mathcal{E}_c^*\mathcal{E}_b}{1-i\omega\tau_l}\Big)\partial_{\mathbf{k}}^b Q_l^c + \Big[\frac{\mathcal{E}_b^*\mathcal{E}_c}{(1+i\omega\tau_l)^2} + \frac{\mathcal{E}_c^*\mathcal{E}_b}{(1-i\omega\tau_l)^2}\Big](\partial_{\mathbf{k}}^b\tau_l)Q_l^c \\
&\quad + \mathcal{E}_b^*\mathcal{E}_c\big(P_l^{bc} - \bar{P}_l^{bc}\big) + \mathcal{E}_c^*\mathcal{E}_b\big(P_l^{bc} - \bar{P}_l^{bc}\big)^* + \tau_l\Big[\frac{\mathcal{E}_b\mathcal{E}_c e^{i2\omega t}}{(1+i\omega\tau_l)(1+i2\omega\tau_l)} + \frac{\mathcal{E}_b^*\mathcal{E}_c^* e^{-i2\omega t}}{(1-i\omega\tau_l)(1-i2\omega\tau_l)}\Big]\partial_{\mathbf{k}}^b Q_l^c \\
&\quad + \Big[\frac{\mathcal{E}_b\mathcal{E}_c e^{i2\omega t}}{(1+i\omega\tau_l)^2(1+i2\omega\tau_l)} + \frac{\mathcal{E}_b^*\mathcal{E}_c^* e^{-i2\omega t}}{(1-i\omega\tau_l)^2(1-i2\omega\tau_l)}\Big](\partial_{\mathbf{k}}^b\tau_l)Q_l^c \\
&\quad + \frac{\mathcal{E}_a\mathcal{E}_b e^{i2\omega t}}{1+i2\omega\tau_l}\big(P_l^{ab} - \bar{P}_l^{ab}\big) + \frac{\mathcal{E}_a^*\mathcal{E}_b^* e^{-i2\omega t}}{1-i2\omega\tau_l}\big(P_l^{ab} - \bar{P}_l^{ab}\big)^*\Big\}\Bigg\} \\
&\equiv \mathrm{Re}\big\{J_a^{sj,2}(0) + J_a^{sj,2}(\omega)e^{i\omega t} + J_a^{sj,2}(2\omega)e^{i2\omega t}\big\} \qquad (48)
\end{aligned}
$$

with

$$
J_a^{sj,2}(0) = \frac{e^3\mathcal{E}_b\mathcal{E}_c^*}{2\hbar}\sum_l \Big\{\frac{\tau_l}{1+i\omega\tau_l}\Big[\varepsilon^{acd}\Omega_l^d Q_l^b + \tau_l v_l^a \partial_{\mathbf{k}}^c Q_l^b + \frac{v_l^a\partial_{\mathbf{k}}^c\tau_l}{1+i\omega\tau_l}Q_l^b\Big] + \tau_l v_l^a\big(P_l^{cb} - \bar{P}_l^{cb}\big)\Big\}, \qquad (49)
$$

$$
J_a^{sj,2}(\omega) = e^2\mathcal{E}_b\sum_l \frac{\tau_l v_l^a}{1+i\omega\tau_l}Q_l^b, \qquad (50)
$$

$$
\begin{aligned}
J_a^{sj,2}(2\omega) &= \frac{e^3\mathcal{E}_b\mathcal{E}_c}{2\hbar}\sum_l \Big\{\frac{\tau_l}{1+i\omega\tau_l}\Big[\varepsilon^{acd}\Omega_l^d Q_l^b + \frac{\tau_l v_l^a}{1+i2\omega\tau_l}\partial_{\mathbf{k}}^c Q_l^b + \frac{v_l^a\partial_{\mathbf{k}}^c\tau_l}{(1+i\omega\tau_l)(1+i2\omega\tau_l)}Q_l^b\Big] \\
&\quad + \frac{\tau_l v_l^a}{1+i2\omega\tau_l}\big(P_l^{cb} - \bar{P}_l^{cb}\big)\Big\}. \qquad (51)
\end{aligned}
$$

Combine these two contributions, the total side-jump contribution can be obtained as

$$
\begin{aligned}
J_a^{sj}(0) &= \frac{e^3\mathcal{E}_b\mathcal{E}_c^*}{2\hbar}\sum_l \Big\{\frac{\tau_l}{1+i\omega\tau_l}\Big[\varepsilon^{acd}\Omega_l^d Q_l^b + \tau_l v_l^a\partial_{\mathbf{k}}^c Q_l^b + \frac{v_l^a\partial_{\mathbf{k}}^c\tau_l}{1+i\omega\tau_l}Q_l^b \\
&\quad - \frac{v_a^{sj}}{\hbar}\Big(\tau_l\partial_{\mathbf{k}}^c\partial_{\mathbf{k}}^b + \frac{\partial_{\mathbf{k}}^c\tau_l}{1+i\omega\tau_l}\partial_{\mathbf{k}}^b\Big)f_l^{(0)}\Big] + \tau_l v_l^a\big(P_l^{cb} - \bar{P}_l^{cb}\big)\Big\}, \qquad (52)
\end{aligned}
$$

$$
J_a^{sj}(\omega) = e^2\mathcal{E}_b\sum_l \frac{\tau_l}{1+i\omega\tau_l}\Big(v_l^a Q_l^b - \frac{v_a^{sj}}{\hbar}\partial_{\mathbf{k}}^b f_l^{(0)}\Big), \qquad (53)
$$

$$
\begin{aligned}
J_a^{sj}(2\omega) &= \frac{e^3\mathcal{E}_b\mathcal{E}_c}{2\hbar}\sum_l \Big\{\frac{\tau_l}{1+i\omega\tau_l}\Big[\varepsilon^{acd}\Omega_l^d Q_l^b + \frac{\tau_l v_l^a}{1+i2\omega\tau_l}\partial_{\mathbf{k}}^c Q_l^b + \frac{v_l^a\partial_{\mathbf{k}}^c\tau_l}{(1+i\omega\tau_l)(1+i2\omega\tau_l)}Q_l^b \\
&\quad - \frac{v_a^{sj}}{\hbar(1+i2\omega\tau_l)}\Big(\tau_l\partial_{\mathbf{k}}^c\partial_{\mathbf{k}}^b + \frac{\partial_{\mathbf{k}}^c\tau_l}{1+i\omega\tau_l}\partial_{\mathbf{k}}^b\Big)f_l^{(0)}\Big] + \frac{\tau_l v_l^a}{1+i2\omega\tau_l}\big(P_l^{cb} - \bar{P}_l^{cb}\big)\Big\}. \qquad (54)
\end{aligned}
$$

Skew-scattering contribution

Put the expression of f_l^{in} into the third equation, we obtain that

$$
\big[\partial_t - \frac{e}{2\hbar}\big(\mathcal{E}_a e^{i\omega t} + \mathcal{E}_a^* e^{-i\omega t}\big)\partial_{\mathbf{k}}^a\big]\delta f_l^{sk} = -\frac{\delta f_l^{sk}}{\tau_l} - \sum_{l'}\varpi_{l'l}^{as}(f_l^{in} + f_{l'}^{in}), \qquad (55)
$$

which can be formally solved as

$$
\begin{aligned}
\delta f_l^{sk} &= \frac{-\tau_l \sum_{l'} \varpi_{l'l}^{as}(f_l^{in}+f_{l'}^{in})}{1+\tau_l\partial_t-(e\tau_l/2\hbar)\big(\mathcal{E}_c e^{i\omega t}+\mathcal{E}_c^* e^{-i\omega t}\big)\partial_{\mathbf{k}}^c} \\
&= -\sum_{n=0}^{\infty}\Big[-\tau_l\partial_t+\frac{e\tau_l}{2\hbar}\big(\mathcal{E}_c e^{i\omega t}+\mathcal{E}_c^* e^{-i\omega t}\big)\partial_{\mathbf{k}}^c\Big]^n \tau_l \sum_{l'} \varpi_{l'l}^{as}(f_l^{in}+f_{l'}^{in}) \\
&= -\sum_{n=0}^{\infty}\Big[-\tau_l\partial_t+\frac{e\tau_l}{2\hbar}\big(\mathcal{E}_c e^{i\omega t}+\mathcal{E}_c^* e^{-i\omega t}\big)\partial_{\mathbf{k}}^c\Big]^n \tau_l \sum_{l'} \varpi_{l'l}^{as}\Bigg\{ f_l^{(0)}+f_{l'}^{(0)}+\frac{e\tau_l}{2\hbar}\Big(\frac{\mathcal{E}_a e^{i\omega t}}{1+i\omega\tau_l} \\
&\quad +\frac{\mathcal{E}_a^* e^{-i\omega t}}{1-i\omega\tau_l}\Big)\partial_{\mathbf{k}}^a f_l^{(0)}+\frac{e\tau_{l'}}{2\hbar}\Big(\frac{\mathcal{E}_a e^{i\omega t}}{1+i\omega\tau_{l'}}+\frac{\mathcal{E}_a^* e^{-i\omega t}}{1-i\omega\tau_{l'}}\Big)\partial_{\mathbf{k}'}^a f_{l'}^{(0)}+\frac{e^2\tau_l}{4\hbar^2}\Big\{\tau_l\Big(\frac{\mathcal{E}_a^*\mathcal{E}_b}{1+i\omega\tau_l}+\frac{\mathcal{E}_b^*\mathcal{E}_a}{1-i\omega\tau_l}\Big)\partial_{\mathbf{k}}^a\partial_{\mathbf{k}}^b f_l^{(0)} \\
&\quad +\Big[\frac{\mathcal{E}_a^*\mathcal{E}_b}{(1+i\omega\tau_l)^2}+\frac{\mathcal{E}_b^*\mathcal{E}_a}{(1-i\omega\tau_l)^2}\Big]\big(\partial_{\mathbf{k}}^a\tau_l\big)\partial_{\mathbf{k}}^b f_l^{(0)}+\tau_l\Big[\frac{\mathcal{E}_a\mathcal{E}_b e^{i2\omega t}}{(1+i\omega\tau_l)(1+i2\omega\tau_l)}+\frac{\mathcal{E}_a^*\mathcal{E}_b^* e^{-i2\omega t}}{(1-i\omega\tau_l)(1-i2\omega\tau_l)}\Big]\partial_{\mathbf{k}}^a\partial_{\mathbf{k}}^b f_l^{(0)} \\
&\quad +\Big[\frac{\mathcal{E}_a\mathcal{E}_b e^{i2\omega t}}{(1+i\omega\tau_l)^2(1+i2\omega\tau_l)}+\frac{\mathcal{E}_a^*\mathcal{E}_b^* e^{-i2\omega t}}{(1-i\omega\tau_l)^2(1-i2\omega\tau_l)}\Big]\big(\partial_{\mathbf{k}}^a\tau_l\big)\partial_{\mathbf{k}}^b f_l^{(0)}\Big\} \\
&\quad +\frac{e^2\tau_{l'}}{4\hbar^2}\Big\{\tau_{l'}\Big(\frac{\mathcal{E}_a^*\mathcal{E}_b}{1+i\omega\tau_{l'}}+\frac{\mathcal{E}_b^*\mathcal{E}_a}{1-i\omega\tau_{l'}}\Big)\partial_{\mathbf{k}'}^a\partial_{\mathbf{k}'}^b f_{l'}^{(0)}+\Big[\frac{\mathcal{E}_a^*\mathcal{E}_b}{(1+i\omega\tau_{l'})^2}+\frac{\mathcal{E}_b^*\mathcal{E}_a}{(1-i\omega\tau_{l'})^2}\Big]\big(\partial_{\mathbf{k}'}^a\tau_{l'}\big)\partial_{\mathbf{k}'}^b f_{l'}^{(0)} \\
&\quad +\tau_{l'}\Big[\frac{\mathcal{E}_a\mathcal{E}_b e^{i2\omega t}}{(1+i\omega\tau_{l'})(1+i2\omega\tau_{l'})}+\frac{\mathcal{E}_a^*\mathcal{E}_b^* e^{-i2\omega t}}{(1-i\omega\tau_{l'})(1-i2\omega\tau_{l'})}\Big]\partial_{\mathbf{k}'}^a\partial_{\mathbf{k}'}^b f_{l'}^{(0)} \\
&\quad +\Big[\frac{\mathcal{E}_a\mathcal{E}_b e^{i2\omega t}}{(1+i\omega\tau_{l'})^2(1+i2\omega\tau_{l'})}+\frac{\mathcal{E}_a^*\mathcal{E}_b^* e^{-i2\omega t}}{(1-i\omega\tau_{l'})^2(1-i2\omega\tau_{l'})}\Big]\big(\partial_{\mathbf{k}'}^a\tau_{l'}\big)\partial_{\mathbf{k}'}^b f_{l'}^{(0)}\Big\}\Bigg\} \\
&\equiv \delta^1 f_l^{sk}+\delta^2 f_l^{sk}.
\end{aligned}
\tag{56}
$$

Note that the equilibrium distribution function does not contribute to the scattering

$$
\sum_{l'} \varpi_{l'l}^{as}(f_l^{(0)}+f_{l'}^{(0)}) = 0. \tag{57}
$$

Thus, the linear dependent part reads

$$
\begin{aligned}
\delta^1 f_l^{sk} &= -\sum_{n=0}^{\infty}(-\tau_l\partial_t)^n\tau_l\sum_{l'}\varpi_{l'l}^{as}\Big[\frac{e\tau_l}{2\hbar}\Big(\frac{\mathcal{E}_a e^{i\omega t}}{1+i\omega\tau_l}+\frac{\mathcal{E}_a^* e^{-i\omega t}}{1-i\omega\tau_l}\Big)\partial_{\mathbf{k}}^a f_l^{(0)} \\
&\quad +\frac{e\tau_{l'}}{2\hbar}\Big(\frac{\mathcal{E}_a e^{i\omega t}}{1+i\omega\tau_{l'}}+\frac{\mathcal{E}_a^* e^{-i\omega t}}{1-i\omega\tau_{l'}}\Big)\partial_{\mathbf{k}'}^a f_{l'}^{(0)}\Big] \\
&= -\frac{e}{2\hbar}\Big[\mathcal{E}_a e^{i\omega t}\big(I_l^a+\bar{I}_l^a\big)+\mathcal{E}_a^* e^{-i\omega t}\big(I_l^a+\bar{I}_l^a\big)^*\Big]
\end{aligned}
\tag{58}
$$

with

$$
I_l^a=\sum_{l'}\varpi_{l'l}^{as}\frac{\tau_l^2\partial_{\mathbf{k}}^a f_l^{(0)}}{(1+i\omega\tau_l)^2}, \qquad \bar{I}_l^a=\sum_{l'}\varpi_{l'l}^{as}\frac{\tau_l\tau_{l'}\partial_{\mathbf{k}'}^a f_{l'}^{(0)}}{(1+i\omega\tau_l)(1+i\omega\tau_{l'})}, \tag{59}
$$

and the second-order part is

$$\begin{aligned}
\delta^2 f_l^{sk} =& -\sum_{n=0}^{\infty}(-\tau_l\partial_t)^n \frac{e\tau_l}{2\hbar}\big(\mathcal{E}_b e^{i\omega t}+\mathcal{E}_b^* e^{-i\omega t}\big)\partial_{\mathbf{k}}^b \frac{e}{2\hbar}\Big[\mathcal{E}_a e^{i\omega t} I_l^a + \mathcal{E}_a^* e^{-i\omega t}(I_l^a)^* + \mathcal{E}_a e^{i\omega t}\bar{I}_l^a + \mathcal{E}_a^* e^{-i\omega t}(\bar{I}_l^a)^*\Big] \\
& -\sum_{n=0}^{\infty}(-\tau_l\partial_t)^n \tau_l \frac{e^2}{4\hbar^2}\Big[\mathcal{E}_a\mathcal{E}_b^*\big(J_1^{ba}+\bar{J}_1^{ba}\big)+\mathcal{E}_a^*\mathcal{E}_b\big(J_1^{ba}+\bar{J}_1^{ba}\big)^*+\mathcal{E}_a\mathcal{E}_b^*\big(K_1^{ba}+\bar{K}_1^{ba}\big)+\mathcal{E}_a^*\mathcal{E}_b\big(K_1^{ba}+\bar{K}_1^{ba}\big)^* \\
& +\mathcal{E}_a\mathcal{E}_b e^{i2\omega t}\big(J_2^{ba}+\bar{J}_2^{ba}\big)+\mathcal{E}_a^*\mathcal{E}_b^* e^{-i2\omega t}\big(J_2^{ba}+\bar{J}_2^{ba}\big)^*+\mathcal{E}_a\mathcal{E}_b e^{i2\omega t}\big(K_2^{ba}+\bar{K}_2^{ba}\big)+\mathcal{E}_a^*\mathcal{E}_b^* e^{-i2\omega t}\big(K_2^{ba}+\bar{K}_2^{ba}\big)^*\Big] \\
=& -\frac{e^2\tau_l}{4\hbar^2}\Big[\mathcal{E}_a\mathcal{E}_b^*\partial_{\mathbf{k}}^b\big(I_l^a+\bar{I}_l^a\big)+\mathcal{E}_b\mathcal{E}_a^*\partial_{\mathbf{k}}^b\big(I_l^a+\bar{I}_l^a\big)^*+\mathcal{E}_a\mathcal{E}_b^*\big(J_1^{ba}+\bar{J}_1^{ba}\big)+\mathcal{E}_a^*\mathcal{E}_b\big(J_1^{ba}+\bar{J}_1^{ba}\big)^* \\
& +\mathcal{E}_a\mathcal{E}_b^*\big(K_1^{ba}+\bar{K}_1^{ba}\big)+\mathcal{E}_a^*\mathcal{E}_b\big(K_1^{ba}+\bar{K}_1^{ba}\big)^*+\frac{\mathcal{E}_a\mathcal{E}_b e^{i2\omega t}}{1+i2\omega\tau_l}\partial_{\mathbf{k}}^b\big(I_l^a+\bar{I}_l^a\big)+\frac{\mathcal{E}_a^*\mathcal{E}_b^* e^{-i2\omega t}}{1-i2\omega\tau_l}\partial_{\mathbf{k}}^b\big(I_l^a+\bar{I}_l^a\big)^* \\
& +\frac{\mathcal{E}_a\mathcal{E}_b e^{i2\omega t}}{1+i2\omega\tau_l}\big(J_2^{ba}+\bar{J}_2^{ba}\big)+\frac{\mathcal{E}_a^*\mathcal{E}_b^* e^{-i2\omega t}}{1-i2\omega\tau_l}\big(J_2^{ba}+\bar{J}_2^{ba}\big)^* \\
& +\frac{\mathcal{E}_a\mathcal{E}_b e^{i2\omega t}}{1+i2\omega\tau_l}\big(K_2^{ba}+\bar{K}_2^{ba}\big)+\frac{\mathcal{E}_a^*\mathcal{E}_b^* e^{-i2\omega t}}{1-i2\omega\tau_l}\big(K_2^{ba}+\bar{K}_2^{ba}\big)^*\Big] \qquad (60)
\end{aligned}$$

with

$$J_1^{ab}=\sum_{l'}\varpi_{l'l}^{as}\frac{\tau_l^2\partial_{\mathbf{k}}^a\partial_{\mathbf{k}}^b f_l^{(0)}}{1+i\omega\tau_l}, \qquad \bar{J}_1^{ab}=\sum_{l'}\varpi_{l'l}^{as}\frac{\tau_{l'}^2\partial_{\mathbf{k}'}^a\partial_{\mathbf{k}'}^b f_{l'}^{(0)}}{1+i\omega\tau_{l'}}, \qquad (61)$$

$$K_1^{ab}=\sum_{l'}\varpi_{l'l}^{as}\frac{\tau_l(\partial_{\mathbf{k}}^a\tau_l)\partial_{\mathbf{k}}^b f_l^{(0)}}{(1+i\omega\tau_l)^2}, \qquad \bar{K}_1^{ab}=\sum_{l'}\varpi_{l'l}^{as}\frac{\tau_{l'}(\partial_{\mathbf{k}'}^a\tau_{l'})\partial_{\mathbf{k}'}^b f_{l'}^{(0)}}{(1+i\omega\tau_{l'})^2}. \qquad (62)$$

$$J_2^{ab}=\sum_{l'}\varpi_{l'l}^{as}\frac{\tau_l^2\partial_{\mathbf{k}}^a\partial_{\mathbf{k}}^b f_l^{(0)}}{(1+i\omega\tau_l)(1+i2\omega\tau_l)}, \qquad \bar{J}_2^{ab}=\sum_{l'}\varpi_{l'l}^{as}\frac{\tau_{l'}^2\partial_{\mathbf{k}'}^a\partial_{\mathbf{k}'}^b f_{l'}^{(0)}}{(1+i\omega\tau_{l'})(1+i2\omega\tau_{l'})}, \qquad (63)$$

$$K_2^{ab}=\sum_{l'}\varpi_{l'l}^{as}\frac{\tau_l(\partial_{\mathbf{k}}^a\tau_l)\partial_{\mathbf{k}}^b f_l^{(0)}}{(1+i\omega\tau_l)^2(1+i2\omega\tau_l)}, \qquad \bar{K}_2^{ab}=\sum_{l'}\varpi_{l'l}^{as}\frac{\tau_{l'}(\partial_{\mathbf{k}'}^a\tau_{l'})\partial_{\mathbf{k}'}^b f_{l'}^{(0)}}{(1+i\omega\tau_{l'})^2(1+i2\omega\tau_{l'})}. \qquad (64)$$

Then the corresponding electric current contribution can be written as

$$\begin{aligned}
J_a^{sk}(\mathbf{E}) =& -e\sum_l\Big[v_l^a+\frac{e}{2\hbar}\varepsilon^{ade}\big(\mathcal{E}_d e^{i\omega t}+\mathcal{E}_d^* e^{-i\omega t}\big)\Omega_l^e\Big]\Big(\delta^1 f_l^{sk}+\delta^2 f_l^{sk}\Big) \\
=& \; e\sum_l\Big[v_l^a+\frac{e}{2\hbar}\varepsilon^{ade}\big(\mathcal{E}_d e^{i\omega t}+\mathcal{E}_d^* e^{-i\omega t}\big)\Omega_l^e\Big]\Big\{\frac{e}{2\hbar}\Big[\mathcal{E}_b e^{i\omega t}\big(I_l^b+\bar{I}_l^b\big)+\mathcal{E}_b^* e^{-i\omega t}\big(I_l^b+\bar{I}_l^b\big)^*\Big] \\
& +\frac{e^2\tau_l}{4\hbar^2}\Big[\mathcal{E}_b\mathcal{E}_c^*\partial_{\mathbf{k}}^c\big(I_l^b+\bar{I}_l^b\big)+\mathcal{E}_c\mathcal{E}_b^*\partial_{\mathbf{k}}^c\big(I_l^b+\bar{I}_l^b\big)^*+\mathcal{E}_b\mathcal{E}_c^*\big(J_1^{cb}+\bar{J}_1^{cb}\big)+\mathcal{E}_b^*\mathcal{E}_c\big(J_1^{cb}+\bar{J}_1^{cb}\big)^* \\
& +\mathcal{E}_b\mathcal{E}_c^*\big(K_1^{cb}+\bar{K}_1^{cb}\big)+\mathcal{E}_b^*\mathcal{E}_c\big(K_1^{cb}+\bar{K}_1^{cb}\big)^*+\frac{\mathcal{E}_a\mathcal{E}_b e^{i2\omega t}}{1+i2\omega\tau_l}\partial_{\mathbf{k}}^b\big(I_l^a+\bar{I}_l^a\big)+\frac{\mathcal{E}_a^*\mathcal{E}_b^* e^{-i2\omega t}}{1-i2\omega\tau_l}\partial_{\mathbf{k}}^b\big(I_l^a+\bar{I}_l^a\big)^* \\
& +\frac{\mathcal{E}_b\mathcal{E}_c e^{i2\omega t}}{1+i2\omega\tau_l}\big(J_2^{cb}+\bar{J}_2^{cb}\big)+\frac{\mathcal{E}_b^*\mathcal{E}_c^* e^{-i2\omega t}}{1-i2\omega\tau_l}\big(J_2^{cb}+\bar{J}_2^{cb}\big)^* \\
& +\frac{\mathcal{E}_b\mathcal{E}_c e^{i2\omega t}}{1+i2\omega\tau_l}\big(K_2^{cb}+\bar{K}_2^{cb}\big)+\frac{\mathcal{E}_b^*\mathcal{E}_c^* e^{-i2\omega t}}{1-i2\omega\tau_l}\big(K_2^{cb}+\bar{K}_2^{cb}\big)^*\Big]\Big\} \\
\equiv& \; \mathrm{Re}\big\{J_a^{sk}(0)+J_a^{sk}(\omega)e^{i\omega t}+J_a^{sk}(2\omega)e^{i2\omega t}\big\} \qquad (65)
\end{aligned}$$

with

$$J_a^{sk}(0)=\frac{e^3\mathcal{E}_b\mathcal{E}_c^*}{2\hbar^2}\sum_l\Big\{\varepsilon^{acd}\Omega_l^d\big(I_l^b+\bar{I}_l^b\big)+\tau_l v_l^a\Big[\partial_{\mathbf{k}}^c\big(I_l^b+\bar{I}_l^b\big)+J_1^{cb}+\bar{J}_1^{cb}+K_1^{cb}+\bar{K}_1^{cb}\Big]\Big\}, \qquad (66)$$

$$J_a^{sk}(\omega)=\frac{e^2\mathcal{E}_b}{\hbar}\sum_l v_l^a\big(I_l^b+\bar{I}_l^b\big), \qquad (67)$$

$$J_a^{sk}(2\omega)=\frac{e^3\mathcal{E}_b\mathcal{E}_c}{2\hbar^2}\sum_l\Big\{\varepsilon^{acd}\Omega_l^d\big(I_l^b+\bar{I}_l^b\big)+\frac{\tau_l v_l^a}{1+i2\omega\tau_l}\Big[\partial_{\mathbf{k}}^c\big(I_l^b+\bar{I}_l^b\big)+J_2^{cb}+\bar{J}_2^{cb}+K_2^{cb}+\bar{K}_2^{cb}\Big]\Big\}. \qquad (68)$$

SUPPLEMENTARY NOTE 3

Anomalous & nonlinear Hall effects: In the presence of a *ac* electric field, the anomalous Hall effect correspond to the response signal with linear frequency, and the nonlinear Hall effect refers to the response signal with zero and double frequency. As we have demonstrated in previous section, all of these response can be obtained within our theory, thus our framework can provide a unified description of the anomalous and nonlinear Hall effects. In this section, we summarize the general expression that we have obtained and discuss their approximate forms in different limits.

Anomalous Hall effect

Anomalous Hall effect can only be observed in systems with broken time reversal symmetry. According to our previous calculation, the anomalous Hall conductivity can be obtained by writing that

$$J_a(\omega) = \sigma^A_{ab}\mathcal{E}_b \cos(\omega t). \tag{69}$$

Thus we obtain that

$$\sigma^A_{ab}(\omega) = \sigma^{in}_{ab}(\omega) + \sigma^{sj,1}_{ab}(\omega) + \sigma^{sj,2}_{ab}(\omega) + \sigma^{sk}_{ab}(\omega) \tag{70}$$

with

$$\sigma^{in}_{ab}(\omega) = -\frac{e^2}{\hbar}\sum_l \varepsilon^{abc}\Omega^c_l f^{(0)}_l, \tag{71}$$

$$\sigma^{sj,1}_{ab}(\omega) = -\frac{e^2}{\hbar}\sum_l \frac{\tau_l v^{sj}_a}{1+i\omega\tau_l}\partial^b_{\mathbf{k}} f^{(0)}_l, \tag{72}$$

$$\sigma^{sj,2}_{ab}(\omega) = e^2\sum_{ll'} \frac{\tau_l v^a_l}{1+i\omega\tau_l} O^b_{ll'}[f^{(0)}_l - f^{(0)}_{l'}], \tag{73}$$

$$\sigma^{sk}_{ab}(\omega) = \frac{e^2}{\hbar}\sum_{ll'} \frac{\tau_l v^a_l \varpi^{as}_{l'l}}{1+i\omega\tau_l}\Big(\frac{\tau_l\partial^b_{\mathbf{k}} f^{(0)}_l}{1+i\omega\tau_l} + \frac{\tau_{l'}\partial^b_{\mathbf{k}'} f^{(0)}_{l'}}{1+i\omega\tau_{l'}}\Big), \tag{74}$$

Here we have neglected the second term in $J^{in}_a(\omega)$, which refers to the planar Hall effect instead of anomalous Hall effect. The above expression contains all the leading contribution to the anomalous Hall effect in the most general form.

Frequency-dependence in the isotropic limit

In the isotropic limit, the general relaxation time τ_l can be considered as a constant τ. Thus the general expressions can be further simplified as

$$\sigma^{in}_{ab}(\omega) = -\frac{e^2}{\hbar}\sum_l \varepsilon^{abc}\Omega^c_l f^{(0)}_l, \tag{75}$$

$$\sigma^{sj,1}_{ab}(\omega) = -\frac{\tau}{1+i\omega\tau}\frac{e^2}{\hbar}\sum_l v^{sj}_a \partial^b_{\mathbf{k}} f^{(0)}_l, \tag{76}$$

$$\sigma^{sj,2}_{ab}(\omega) = \frac{\tau}{1+i\omega\tau}\frac{e^2}{\hbar}\sum_{ll'} v^{sj}_b \partial^a_{\mathbf{k}} f^{(0)}_l, \tag{77}$$

$$\sigma^{sk}_{ab}(\omega) = -\frac{\tau^2}{(1+i\omega\tau)^2}\frac{e^2}{\hbar}\sum_{ll'} \varpi^{as}_{ll'}(v^a_l - v^a_{l'})\partial^b_{\mathbf{k}} f^{(0)}_l, \tag{78}$$

where we have performed an integration by parts in $\sigma^{sj,2}_{ab}(\omega)$ by noting that $v^a_l(\partial/\partial\varepsilon_l)\delta(\varepsilon_l - \varepsilon_{l'}) = (1/\hbar)\partial^a_{\mathbf{k}}\delta(\varepsilon_l - \varepsilon_{l'})$. It is interesting to note that the intrinsic, side-jump and skew-scattering contributions have different frequency dependence. This feature is most significant when considering the high frequency limit ($\omega\tau \gg 1$), in which all the contributions are irrelevant with τ and we have $\sigma^{in}_{ab}(\omega) \propto \omega^0$, $\sigma^{sj}_{ab}(\omega) \propto \omega^{-1}$ and $\sigma^{sk}_{ab}(\omega) \propto \omega^{-2}$.

Anomalous Hall conductivity in the low-frequency limit

The typical frequency of the *ac* electric field is about 10-1000 Hz, which is the key difference with the optical measurements. Consequently, the frequency dependence in the denominator can be neglected because the relaxation time is about 1-100 ps, which indicate that $\omega\tau \ll 1$. Thus, in this limit, the the general expressions become

$$\sigma_{ab}^{in} = -\frac{e^2}{\hbar}\sum_l \varepsilon^{abc}\Omega_l^c f_l^{(0)}, \tag{79}$$

$$\sigma_{ab}^{sj,1} = -\frac{e^2}{\hbar}\sum_l \tau_l v_a^{sj}\partial_{\mathbf{k}}^b f_l^{(0)}, \tag{80}$$

$$\sigma_{ab}^{sj.2} = \frac{e^2}{\hbar}\sum_l \tau_l v_b^{sj}\partial_{\mathbf{k}}^a f_l^{(0)}, \tag{81}$$

$$\sigma_{ab}^{sk} = -\frac{e^2}{\hbar}\sum_{ll'} \tau_l \varpi_{ll'}^{as}(\tau_l v_l^a - \tau_{l'} v_{l'}^a)\partial_{\mathbf{k}}^b f_l^{(0)}, \tag{82}$$

where all the contributions are frequency independent.

Nonlinear Hall effect in $\mathcal{T}$-broken systems

Different with the anomalous Hall effect, the nonlinear Hall effect can appear in both $\mathcal{T}$-broken and $\mathcal{T}$-symmetric cases. Although our main focus is the $\mathcal{T}$-symmetric case, here we list out the general expression of the $\mathcal{T}$-broken case for completeness. By writing

$$J_a(0) = \xi_{abc}\mathcal{E}_b\mathcal{E}_c^*, \tag{83}$$

$$J_a(2\omega) = \chi_{abc}\mathcal{E}_b\mathcal{E}_c\cos(2\omega t), \tag{84}$$

we can define the nonlinear Hall conductivity corresponding to the zero-frequency response as

$$\xi_{abc}(\omega) = \xi_{abc}^{in}(\omega) + \xi_{abc}^{sj,1}(\omega) + \xi_{abc}^{sj,2}(\omega) + \xi_{abc}^{sk}(\omega), \tag{85}$$

where

$$\xi_{abc}^{in}(\omega) = -\frac{e^3}{2\hbar^2}\sum_l \frac{\tau_l}{1+i\omega\tau_l}\Big(\varepsilon^{acd}\Omega_l^d\partial_{\mathbf{k}}^b + \tau_l v_l^a\partial_{\mathbf{k}}^c\partial_{\mathbf{k}}^b + \frac{v_l^a\partial_{\mathbf{k}}^c\tau_l}{1+i\omega\tau_l}\partial_{\mathbf{k}}^b\Big)f_l^{(0)}, \tag{86}$$

$$\xi_{abc}^{sj,1}(\omega) = -\frac{e^3}{2\hbar^2}\sum_l \frac{\tau_l v_a^{sj}}{1+i\omega\tau_l}\Big(\tau_l\partial_{\mathbf{k}}^c\partial_{\mathbf{k}}^b + \frac{\partial_{\mathbf{k}}^c\tau_l}{1+i\omega\tau_l}\partial_{\mathbf{k}}^b\Big)f_l^{(0)}, \tag{87}$$

$$\begin{aligned}\xi_{abc}^{sj,2}(\omega) = \frac{e^3}{2\hbar}\sum_{ll'}\Big\{&\frac{\tau_l}{1+i\omega\tau_l}\Big(\tau_l v_l^a\partial_{\mathbf{k}}^c + \frac{v_l^a\partial_{\mathbf{k}}^c\tau_l}{1+i\omega\tau_l}\Big)\Big[O_{ll'}^b(f_l^{(0)} - f_{l'}^{(0)})\Big]\\ &+\tau_l v_l^a O_{ll'}^c\Big[\frac{\tau_l\partial_{\mathbf{k}}^b f_l^{(0)}}{1+i\omega\tau_l} - \frac{\tau_{l'}\partial_{\mathbf{k}'}^b f_{l'}^{(0)}}{1+i\omega\tau_{l'}}\Big]\Big\},\end{aligned} \tag{88}$$

$$\begin{aligned}\xi_{abc}^{sk}(\omega) = -\frac{e^3}{2\hbar^2}\sum_{ll'}\tau_l\varpi_{l'l}^{as}\Big\{&\frac{\partial_{\mathbf{k}}^c(\tau_l v_l^a)}{1+i\omega\tau_l}\Big(\frac{\tau_l\partial_{\mathbf{k}}^b f_l^{(0)}}{1+i\omega\tau_l} + \frac{\tau_{l'}\partial_{\mathbf{k}'}^b f_{l'}^{(0)}}{1+i\omega\tau_{l'}}\Big)\\ &-v_l^a\Big[\frac{\tau_l^2\partial_{\mathbf{k}}^c\partial_{\mathbf{k}}^b f_l^{(0)}}{1+i\omega\tau_l} + \frac{\tau_{l'}^2\partial_{\mathbf{k}'}^c\partial_{\mathbf{k}'}^b f_{l'}^{(0)}}{1+i\omega\tau_{l'}} + \frac{\tau_l(\partial_{\mathbf{k}}^c\tau_l)\partial_{\mathbf{k}}^b f_l^{(0)}}{(1+i\omega\tau_l)^2} + \frac{\tau_{l'}(\partial_{\mathbf{k}'}^c\tau_{l'})\partial_{\mathbf{k}'}^b f_{l'}^{(0)}}{(1+i\omega\tau_{l'})^2}\Big]\Big\}.\end{aligned} \tag{89}$$

And the nonlinear Hall conductivity corresponding to the double-frequency response can be written as

$$\chi_{abc}(\omega) = \chi_{abc}^{in}(\omega) + \chi_{abc}^{sj,1}(\omega) + \chi_{abc}^{sj,2}(\omega) + \chi_{abc}^{sk}(\omega), \tag{90}$$

where

$$\chi^{in}_{abc}(\omega) = -\frac{e^3}{2\hbar^2}\sum_l \frac{\tau_l}{1+i\omega\tau_l}\Big[\varepsilon^{acd}\Omega^d_l\partial^b_{\mathbf{k}} + \frac{\tau_l v^a_l}{1+i2\omega\tau_l}\partial^c_{\mathbf{k}}\partial^b_{\mathbf{k}} + \frac{v^a_l\partial^c_{\mathbf{k}}\tau_l}{(1+i\omega\tau_l)(1+i2\omega\tau_l)}\partial^b_{\mathbf{k}}\Big] f^{(0)}_l, \tag{91}$$

$$\chi^{sj,1}_{abc}(\omega) = -\frac{e^3}{2\hbar^2}\sum_l \frac{\tau_l v^{sj}_a}{(1+i\omega\tau_l)(1+i2\omega\tau_l)}\Big(\tau_l\partial^c_{\mathbf{k}}\partial^b_{\mathbf{k}} + \frac{\partial^c_{\mathbf{k}}\tau_l}{1+i\omega\tau_l}\partial^b_{\mathbf{k}}\Big) f^{(0)}_l, \tag{92}$$

$$\chi^{sj,2}_{abc}(\omega) = \frac{e^3}{2\hbar}\sum_{ll'}\Big\{\frac{\tau_l}{(1+i\omega\tau_l)(1+i2\omega\tau_l)}\Big(\tau_l v^a_l\partial^c_{\mathbf{k}} + \frac{v^a_l\partial^c_{\mathbf{k}}\tau_l}{1+i\omega\tau_l}\Big)\Big[O^b_{ll'}(f^{(0)}_l - f^{(0)}_{l'})\Big] + \frac{\tau_l v^a_l}{1+i2\omega\tau_l}O^c_{ll'}\Big[\frac{\tau_l\partial^b_{\mathbf{k}}f^{(0)}_l}{1+i\omega\tau_l} - \frac{\tau_{l'}\partial^b_{\mathbf{k}'}f^{(0)}_{l'}}{1+i\omega\tau_{l'}}\Big]\Big\}, \tag{93}$$

$$\chi^{sk}_{abc}(\omega) = -\frac{e^3}{2\hbar^2}\sum_{ll'}\frac{\tau_l\varpi^{as}_{l'l}}{1+i2\omega\tau_l}\Big\{\Big(\tau_l\partial^c_{\mathbf{k}}v^a_l + \frac{v^a_l\partial^c_{\mathbf{k}}\tau_l}{1+i2\omega\tau_l}\Big)\Big(\frac{\tau_l\partial^b_{\mathbf{k}}f^{(0)}_l}{1+i\omega\tau_l} + \frac{\tau_{l'}\partial^b_{\mathbf{k}'}f^{(0)}_{l'}}{1+i\omega\tau_{l'}}\Big) - v^a_l\Big[\frac{\tau^2_l\partial^c_{\mathbf{k}}\partial^b_{\mathbf{k}}f^{(0)}_l}{(1+i\omega\tau_l)(1+i2\omega\tau_l)} + \frac{\tau^2_{l'}\partial^c_{\mathbf{k}'}\partial^b_{\mathbf{k}'}f^{(0)}_{l'}}{(1+i\omega\tau_{l'})(1+i2\omega\tau_{l'})} + \frac{\tau_l(\partial^c_{\mathbf{k}}\tau_l)\partial^b_{\mathbf{k}}f^{(0)}_l}{(1+i\omega\tau_l)^2(1+i2\omega\tau_l)} + \frac{\tau_{l'}(\partial^c_{\mathbf{k}'}\tau_{l'})\partial^b_{\mathbf{k}'}f^{(0)}_{l'}}{(1+i\omega\tau_{l'})^2(1+i2\omega\tau_{l'})}\Big]\Big\}. \tag{94}$$

Here we have neglected the terms $\Omega_l\delta^1 f^{sj}_l$ and $\Omega_l\delta^1 f^{sk}_l$, which are of order $(n_iV_0^2)^0$ (see Sec.). However, the the leading order terms of the nonlinear Hall conductivity in the $\mathcal{T}$-broken systems are the second and third terms of χ^{in}, which are of order $(n_iV_0^2)^{-2}$. These terms may be considered as a nonlinear generalization of the linear planar Hall effect, which can also describe the longitudinal nonlinear response if one take $a=b=c$.

Frequency-dependence in the isotropic limit

In the isotropic limit, the general relaxation time τ_l can be considered as a constant τ. Thus the general expressions of the zero-frequency nonlinear conductivity can be further simplified as

$$\xi^{in}_{abc}(\omega) = -\frac{\tau}{1+i\omega\tau}\frac{e^3}{2\hbar^2}\sum_l(\varepsilon^{acd}\Omega^d_l\partial^b_{\mathbf{k}} + \tau_l v^a_l\partial^c_{\mathbf{k}}\partial^b_{\mathbf{k}})f^{(0)}_l, \tag{95}$$

$$\xi^{sj,1}_{abc}(\omega) = \frac{\tau^2}{1+i\omega\tau}\frac{e^3}{2\hbar^2}\sum_l(\partial^c_{\mathbf{k}}v^{sj}_a)\partial^b_{\mathbf{k}}f^{(0)}_l, \tag{96}$$

$$\xi^{sj,2}_{abc}(\omega) = -\frac{\tau^2}{1+i\omega\tau}\frac{e^3}{2\hbar}\sum_l\Big[(\partial^a_{\mathbf{k}}v^{sj}_c + \mathcal{M}^{ac}_l)v^b_l + (\partial^c_{\mathbf{k}}v^a_l)v^{sj}_b\Big]\frac{\partial f^{(0)}_l}{\partial\varepsilon_l}, \tag{97}$$

$$\xi^{sk}_{abc}(\omega) = \frac{\tau^3}{1+i\omega\tau}\frac{e^3}{2\hbar^2}\sum_{ll'}\varpi^{as}_{ll'}\Big[\frac{\partial^c_l v^a_l - \partial^c_{\mathbf{k}'}v^a_{l'}}{1+i\omega\tau}\partial^b_{\mathbf{k}}f^{(0)}_l - (v^a_l - v^a_{l'})\partial^c_{\mathbf{k}}\partial^b_{\mathbf{k}}f^{(0)}_l\Big]. \tag{98}$$

The detailed derivation of $\xi^{sj,2}_{abc}(\omega)$ and the expression of $\mathcal{M}^{ab}_l$ can be found in Supplementary Methods. . And the general expressions of the double-frequency nonlinear conductivity can be further simplified as

$$\chi^{in}_{abc}(\omega) = -\frac{\tau}{1+i\omega\tau}\frac{e^3}{2\hbar^2}\sum_l\Big[\varepsilon^{acd}\Omega^d_l\partial^b_{\mathbf{k}} + \frac{\tau_l v^a_l}{1+i2\omega\tau_l}\partial^c_{\mathbf{k}}\partial^b_{\mathbf{k}}\Big]f^{(0)}_l, \tag{99}$$

$$\chi^{sj,1}_{abc}(\omega) = \frac{\tau^2}{(1+i\omega\tau)(1+i2\omega\tau)}\frac{e^3}{2\hbar^2}\sum_l(\partial^c_{\mathbf{k}}v^{sj}_a)\partial^b_{\mathbf{k}}f^{(0)}_l, \tag{100}$$

$$\chi^{sj,2}_{abc}(\omega) = -\frac{\tau^2}{(1+i\omega\tau)(1+i2\omega\tau)}\frac{e^3}{2\hbar}\sum_l\Big[(\partial^a_{\mathbf{k}}v^{sj}_c + \mathcal{M}^{ac}_l)v^b_l + (\partial^c_{\mathbf{k}}v^a_l)v^{sj}_b\Big]\frac{\partial f^{(0)}_l}{\partial\varepsilon_l}, \tag{101}$$

$$\chi^{sk}_{abc}(\omega) = \frac{\tau^3}{(1+i\omega\tau)(1+i2\omega\tau)}\frac{e^3}{2\hbar^2}\sum_{ll'}\varpi^{as}_{ll'}\Big[(\partial^c_{\mathbf{k}}v^a_l - \partial^c_{\mathbf{k}'}v^a_{l'})\partial^b_{\mathbf{k}}f^{(0)}_l - \frac{v^a_l - v^a_{l'}}{1+i2\omega\tau}\partial^c_{\mathbf{k}}\partial^b_{\mathbf{k}}f^{(0)}_l\Big]. \tag{102}$$

The zero- and double-frequency nonlinear Hall conductivity demonstrated quite different frequency dependence on the response magnitude. In the high frequency limit, we have $\xi^{in}_{abc}(\omega) \propto \omega^{-1}$ and $\chi^{in}_{abc}(\omega) \propto \omega^{-1}+\omega^{-2}$. And $\xi^{sj}_{abc}(\omega) \propto \omega^{-1}$, $\chi^{sj}_{abc}(\omega) \propto \omega^{-2}$, $\xi^{sk}_{abc}(\omega) \propto \omega^{-1}+\omega^{-2}$ and $\chi^{sk}_{abc}(\omega) \propto \omega^{-2}+\omega^{-3}$.

Nonlinear Hall conductivity in the low-frequency limit

In the low-frequency ($\omega\tau \ll 1$) limit, all the contributions to the zero- and double-frequency nonlinear Hall conductivity become identical, which read

$$\xi^{in}_{abc} = \chi^{in}_{abc} = -\frac{e^3}{2\hbar^2}\sum_l \tau_l[\varepsilon^{acd}\Omega^d_l\partial^b_{\mathbf{k}}f^{(0)}_l + v^a_l\partial^c_{\mathbf{k}}(\tau_l\partial^b_{\mathbf{k}}f^{(0)}_l)], \tag{103}$$

$$\xi^{sj,1}_{abc} = \chi^{sj,1}_{abc} = \frac{e^3}{2\hbar^2}\sum_l \tau_l\partial^c_{\mathbf{k}}(\tau_l v^{sj}_a)\partial^b_{\mathbf{k}}f^{(0)}_l, \tag{104}$$

$$\xi^{sj,2}_{abc} = \chi^{sj,2}_{abc} = -\frac{e^3}{2\hbar}\sum_l \tau_l\Big\{[\partial^a_{\mathbf{k}}(\tau_l v^{sj}_c) + \tilde{\mathcal{M}}^{ac}_l]v^b_l + \partial^c_{\mathbf{k}}(\tau_l v^a_l)v^{sj}_b\Big\}\frac{\partial f^{(0)}_l}{\partial\varepsilon_l}, \tag{105}$$

$$\xi^{sk}_{abc} = \chi^{sk}_{abc} = \frac{e^3}{2\hbar^2}\sum_{ll'} \tau_l\varpi^{as}_{ll'}\Big\{[\tau_l\partial^c_{\mathbf{k}}(\tau_l v^a_l) - \tau_{l'}\partial^c_{\mathbf{k}'}(\tau_{l'}v^a_{l'})]\partial^b_{\mathbf{k}}f^{(0)}_l - (\tau_l v^a_l - \tau_{l'}v^a_{l'})\partial^c_{\mathbf{k}}(\tau_l\partial^b_{\mathbf{k}}f^{(0)}_l)\Big\}, \tag{106}$$

where the expression of $\tilde{\mathcal{M}}^{ab}_l$ can be found in Supplementary Methods. .

Nonlinear Hall effect in $\mathcal{T}$-symmetric systems

Contrary to the anomalous Hall effect, we are more interested in the nonlinear Hall response in the presence of the time reversal symmetry. Note that the usual terms $\mathbf{v}_l$, $\partial^a_{\mathbf{k}}$ and $\mathbf{\Omega}_l$ are odd under time reversal while τ_l is time reversal even. Thus, for time reversal symmetric systems, the only surviving terms in the intrinsic nonlinear currents are those associated with the Berry curvature. For the side-jump contributions, note that $\delta\mathbf{r}_{ll'} \propto \mathbf{\Omega}\times(\mathbf{k}'-\mathbf{k})$, thus $\mathbf{v}^{sj}_l$ and $\mathbf{O}_{ll'}$ are even under time reversal. Also note that $\varpi^{as}_{ll'}$ is odd under time reversal, thus the zero-frequency nonlinear Hall conductivity in $\mathcal{T}$-symmetric systems become

$$\xi^{in}_{abc}(\omega) = -\frac{e^3}{2\hbar^2}\sum_l \frac{\tau_l}{1+i\omega\tau_l}\varepsilon^{acd}\Omega^d_l\partial^b_{\mathbf{k}}f^{(0)}_l, \tag{107}$$

$$\xi^{sj,1}_{abc}(\omega) = -\frac{e^3}{2\hbar^2}\sum_l \frac{\tau_l v^{sj}_a}{1+i\omega\tau_l}\Big(\tau_l\partial^c_{\mathbf{k}}\partial^b_{\mathbf{k}} + \frac{\partial^c_{\mathbf{k}}\tau_l}{1+i\omega\tau_l}\partial^b_{\mathbf{k}}\Big)f^{(0)}_l, \tag{108}$$

$$\begin{aligned}\xi^{sj,2}_{abc}(\omega) = \frac{e^3}{2\hbar}\sum_{ll'}\Big\{&\frac{\tau_l}{1+i\omega\tau_l}\Big(\tau_l v^a_l\partial^c_{\mathbf{k}} + \frac{v^a_l\partial^c_{\mathbf{k}}\tau_l}{1+i\omega\tau_l}\Big)\Big[O^b_{ll'}(f^{(0)}_l - f^{(0)}_{l'})\Big]\\ &+\tau_l v^a_l O^c_{ll'}\Big[\frac{\tau_l\partial^b_{\mathbf{k}}f^{(0)}_l}{1+i\omega\tau_l} - \frac{\tau_{l'}\partial^b_{\mathbf{k}'}f^{(0)}_{l'}}{1+i\omega\tau_{l'}}\Big]\Big\},\end{aligned} \tag{109}$$

$$\begin{aligned}\xi^{sk}_{abc}(\omega) = -\frac{e^3}{2\hbar^2}\sum_{ll'}\tau_l\varpi^{as}_{l'l}\Big\{&\frac{\partial^c_{\mathbf{k}}(\tau_l v^a_l)}{1+i\omega\tau_l}\Big(\frac{\tau_l\partial^b_{\mathbf{k}}f^{(0)}_l}{1+i\omega\tau_l} + \frac{\tau_{l'}\partial^b_{\mathbf{k}'}f^{(0)}_{l'}}{1+i\omega\tau_{l'}}\Big)\\ &-v^a_l\Big[\frac{\tau^2_l\partial^c_{\mathbf{k}}\partial^b_{\mathbf{k}}f^{(0)}_l}{1+i\omega\tau_l} + \frac{\tau^2_{l'}\partial^c_{\mathbf{k}'}\partial^b_{\mathbf{k}'}f^{(0)}_{l'}}{1+i\omega\tau_{l'}} + \frac{\tau_l(\partial^c_{\mathbf{k}}\tau_l)\partial^b_{\mathbf{k}}f^{(0)}_l}{(1+i\omega\tau_l)^2} + \frac{\tau_{l'}(\partial^c_{\mathbf{k}'}\tau_{l'})\partial^b_{\mathbf{k}'}f^{(0)}_{l'}}{(1+i\omega\tau_{l'})^2}\Big]\Big\}.\end{aligned} \tag{110}$$

And the double-frequency nonlinear Hall conductivity in $\mathcal{T}$-symmetric systems become

$$\chi^{in}_{abc}(\omega) = -\frac{e^3}{2\hbar^2}\sum_l \frac{\tau_l}{1+i\omega\tau_l}\varepsilon^{acd}\Omega^d_l \partial^b_{\mathbf{k}} f^{(0)}_l, \tag{111}$$

$$\chi^{sj,1}_{abc}(\omega) = -\frac{e^3}{2\hbar^2}\sum_l \frac{\tau_l v^{sj}_a}{(1+i\omega\tau_l)(1+i2\omega\tau_l)}\Big(\tau_l\partial^c_{\mathbf{k}}\partial^b_{\mathbf{k}} + \frac{\partial^c_{\mathbf{k}}\tau_l}{1+i\omega\tau_l}\partial^b_{\mathbf{k}}\Big) f^{(0)}_l, \tag{112}$$

$$\begin{aligned}\chi^{sj,2}_{abc}(\omega) = \frac{e^3}{2\hbar}\sum_{ll'}\Big\{&\frac{\tau_l}{1+i\omega\tau_l}\Big[\frac{\tau_l v^a_l}{1+i2\omega\tau_l}\partial^c_{\mathbf{k}} + \frac{v^a_l\partial^c_{\mathbf{k}}\tau_l}{(1+i\omega\tau_l)(1+i2\omega\tau_l)}\Big]\Big[O^b_{ll'}(f^{(0)}_l - f^{(0)}_{l'})\Big] \\ &+ \frac{\tau_l v^a_l}{1+i2\omega\tau_l}O^c_{ll'}\Big[\frac{\tau_l\partial^b_{\mathbf{k}} f^{(0)}_l}{1+i\omega\tau_l} - \frac{\tau_{l'}\partial^b_{\mathbf{k}'} f^{(0)}_{l'}}{1+i\omega\tau_{l'}}\Big]\Big\},\end{aligned} \tag{113}$$

$$\begin{aligned}\chi^{sk}_{abc}(\omega) = -\frac{e^3}{2\hbar^2}\sum_{ll'}\frac{\tau_l\varpi^{as}_{l'l}}{1+i2\omega\tau_l}\Big\{&\Big(\tau_l\partial^c_{\mathbf{k}}v^a_l + \frac{v^a_l\partial^c_{\mathbf{k}}\tau_l}{1+i2\omega\tau_l}\Big)\Big(\frac{\tau_l\partial^b_{\mathbf{k}} f^{(0)}_l}{1+i\omega\tau_l} + \frac{\tau_{l'}\partial^b_{\mathbf{k}'} f^{(0)}_{l'}}{1+i\omega\tau_{l'}}\Big) \\ &- v^a_l\Big[\frac{\tau^2_l\partial^c_{\mathbf{k}}\partial^b_{\mathbf{k}} f^{(0)}_l}{(1+i\omega\tau_l)(1+i2\omega\tau_l)} + \frac{\tau^2_{l'}\partial^c_{\mathbf{k}'}\partial^b_{\mathbf{k}'} f^{(0)}_{l'}}{(1+i\omega\tau_{l'})(1+i2\omega\tau_{l'})} \\ &+ \frac{\tau_l(\partial^c_{\mathbf{k}}\tau_l)\partial^b_{\mathbf{k}} f^{(0)}_l}{(1+i\omega\tau_l)^2(1+i2\omega\tau_l)} + \frac{\tau_{l'}(\partial^c_{\mathbf{k}'}\tau_{l'})\partial^b_{\mathbf{k}'} f^{(0)}_{l'}}{(1+i\omega\tau_{l'})^2(1+i2\omega\tau_{l'})}\Big]\Big\}.\end{aligned} \tag{114}$$

The above expression contains all the leading contribution to the nonlinear Hall effect in the most general form, where all the terms are of order $(n_i V_0^2)^{-1}$ (see Sec.). Compared with the $\mathcal{T}$-broken case, only the intrinsic contributions are affected, in which only the Berry dipole contribution can survives in $\mathcal{T}$-symmetric systems.

Frequency-dependence in the isotropic limit

In the isotropic limit, the general relaxation time τ_l can be considered as a constant τ. Thus the general expressions of the zero-frequency nonlinear conductivity can be further simplified as

$$\xi^{in}_{abc}(\omega) = -\frac{\tau}{1+i\omega\tau}\frac{e^3}{2\hbar^2}\sum_l \varepsilon^{acd}\Omega^d_l\partial^b_{\mathbf{k}} f^{(0)}_l, \tag{115}$$

$$\xi^{sj,1}_{abc}(\omega) = \frac{\tau^2}{1+i\omega\tau}\frac{e^3}{2\hbar^2}\sum_l (v^{sj}_a\partial^c_{\mathbf{k}})\partial^b_{\mathbf{k}} f^{(0)}_l, \tag{116}$$

$$\xi^{sj,2}_{abc}(\omega) = -\frac{\tau^2}{1+i\omega\tau}\frac{e^3}{2\hbar}\sum_l \Big[(\partial^a_{\mathbf{k}}v^{sj}_c + \mathcal{M}^{ac}_l)v^b_l + (\partial^c_{\mathbf{k}}v^a_l)v^{sj}_b\Big]\frac{\partial f^{(0)}_l}{\partial\varepsilon_l}, \tag{117}$$

$$\xi^{sk}_{abc}(\omega) = \frac{\tau^3}{1+i\omega\tau}\frac{e^3}{2\hbar^2}\sum_{ll'}\varpi^{as}_{ll'}\Big[\frac{\partial^c_{\mathbf{k}}v^a_l - \partial^c_{\mathbf{k}'}v^a_{l'}}{1+i\omega\tau}\partial^b_{\mathbf{k}} f^{(0)}_l - (v^a_l - v^a_{l'})\partial^c_{\mathbf{k}}\partial^b_{\mathbf{k}} f^{(0)}_l\Big]. \tag{118}$$

The general expressions of the double-frequency nonlinear conductivity can be further simplified as

$$\chi^{in}_{abc}(\omega) = -\frac{\tau}{1+i\omega\tau}\frac{e^3}{2\hbar^2}\sum_l \varepsilon^{acd}\Omega^d_l\partial^b_{\mathbf{k}} f^{(0)}_l, \tag{119}$$

$$\chi^{sj,1}_{abc}(\omega) = \frac{\tau^2}{(1+i\omega\tau)(1+i2\omega\tau)}\frac{e^3}{2\hbar^2}\sum_l (\partial^c_{\mathbf{k}}v^{sj}_a)\partial^b_{\mathbf{k}} f^{(0)}_l, \tag{120}$$

$$\chi^{sj,2}_{abc}(\omega) = -\frac{\tau^2}{(1+i\omega\tau)(1+i2\omega\tau)}\frac{e^3}{2\hbar}\sum_l \Big[(\partial^a_{\mathbf{k}}v^{sj}_c + \mathcal{M}^{ac}_l)v^b_l + (\partial^c_{\mathbf{k}}v^a_l)v^{sj}_b\Big]\frac{\partial f^{(0)}_l}{\partial\varepsilon_l}, \tag{121}$$

$$\chi^{sk}_{abc}(\omega) = \frac{\tau^3}{(1+i\omega\tau)(1+i2\omega\tau)}\frac{e^3}{2\hbar^2}\sum_{ll'}\varpi^{as}_{ll'}\Big[(\partial^c_{\mathbf{k}}v^a_l - \partial^c_{\mathbf{k}'}v^a_{l'})\partial^b_{\mathbf{k}} f^{(0)}_l - \frac{v^a_l - v^a_{l'}}{1+i2\omega\tau}\partial^c_{\mathbf{k}}\partial^b_{\mathbf{k}} f^{(0)}_l\Big]. \tag{122}$$

Compared with the $\mathcal{T}$-broken case, now we have $\xi^{in}_{abc}(\omega) = \chi^{in}_{abc}(\omega) \propto \omega^{-1}$.

Nonlinear Hall conductivity in the low-frequency limit

In the low-frequency ($\omega\tau \ll 1$) limit, the contributions to the zero- and double-frequency nonlinear Hall conductivity are identical, which read

$$\xi^{in}_{abc} = \chi^{in}_{abc} = -\frac{e^3}{2\hbar^2}\sum_l \tau_l \varepsilon^{acd}\Omega^d_l \partial^b_{\mathbf{k}} f^{(0)}_l, \tag{123}$$

$$\xi^{sj,1}_{abc} = \chi^{sj,1}_{abc} = \frac{e^3}{2\hbar^2}\sum_l \tau_l \partial^c_{\mathbf{k}}(\tau_l v^{sj}_a)\partial^b_{\mathbf{k}} f^{(0)}_l, \tag{124}$$

$$\xi^{sj,2}_{abc} = \chi^{sj,2}_{abc} = -\frac{e^3}{2\hbar}\sum_l \tau_l \Big\{[\partial^a_{\mathbf{k}}(\tau_l v^{sj}_c) + \tilde{\mathcal{M}}^{ac}_l]v^b_l + \partial^c_{\mathbf{k}}(\tau_l v^a_l)v^{sj}_b\Big\}\frac{\partial f^{(0)}_l}{\partial \varepsilon_l}, \tag{125}$$

$$\xi^{sk}_{abc} = \chi^{sk}_{abc} = \frac{e^3}{2\hbar^2}\sum_{ll'} \tau_l \varpi^{as}_{ll'}\Big\{[\tau_l\partial^c_{\mathbf{k}}(\tau_l v^a_l) - \tau_{l'}\partial^c_{\mathbf{k}'}(\tau_{l'}v^a_{l'})]\partial^b_{\mathbf{k}} f^{(0)}_l - (\tau_l v^a_l - \tau_{l'}v^a_{l'})\partial^c_{\mathbf{k}}(\tau_l \partial^b_{\mathbf{k}} f^{(0)}_l)\Big\}. \tag{126}$$

Disorder-dependence and mixed contributions

According to the expressions, one can deduce the dependence of the distribution corrections on the impurity concentration by noting that

$$\delta^1 f^{in}_l \sim (n_i V_0^2)^{-1}, \qquad \delta^1 f^{sj}_l \sim (n_i V_0^2)^0, \qquad \delta^1 f^{sk}_l \sim (n_i V_0^2)^0. \tag{127}$$

where we considered the Gaussian asymmetric scattering rate $\varpi^{(4a)}_{ll'} \sim (n_i V_0^2)^2$ as the leading contribution to $\varpi^{as}_{ll'}$. Also note that the side-jump velocity

$$v^{sj}_l \sim n_i V_0^2, \tag{128}$$

thus the leading anomalous Hall contributions

$$\sigma^{in} \propto \Omega_l f^{(0)}_l \sim (n_i V_0^2)^0, \qquad \sigma^{sj} \propto v^{sj}_l \delta^1 f^{in}_l \sim (n_i V_0^2)^0, \qquad \sigma^{sk} \propto v_l \delta^1 f^{sk}_l \sim (n_i V_0^2)^0 \tag{129}$$

are of the same order. A significant aspect of the anomalous Hall effect is that all the leading order contributions are disorder concentration independent.

For the nonlinear Hall effect, the Berry dipole, side-jump and skew-scattering contributions are all of order $(n_i V_0^2)^{-1}$, which can be estimated by noting that

$$\delta^2 f^{in}_l \sim (n_i V_0^2)^{-2}, \qquad \delta^2 f^{sj}_l \sim (n_i V_0^2)^{-1}, \qquad \delta^2 f^{sk}_l \sim (n_i V_0^2)^{-1}, \tag{130}$$

and

$$\chi^{bd} \propto v_l \Omega_l \delta^1 f^{in}_l \sim (n_i V_0^2)^{-1}, \qquad \chi^{sj} \propto v^{sj}_l \delta^2 f^{in}_l \sim (n_i V_0^2)^{-1}, \qquad \chi^{sk} \propto v_l \delta^2 f^{sk}_l \sim (n_i V_0^2)^{-1}. \tag{131}$$

Here we use ξ^{bd} and χ^{bd} to represent the Berry dipole contribution, which the first term of χ^{in} in the $\mathcal{T}$-broken case and equals to χ^{in} in the $\mathcal{T}$-symmetric case.

In the derivation of the anomalous and nonlinear Hall conductivities, we have neglected several mixed contributions such as the terms with mixed side-jump and skew-scattering contributions, the terms with mixed Berry curvature and side-jump contributions, and the terms with mixed Berry curvature and skew-scattering contributions. For the anomalous Hall conductivity, only the terms with mixed side-jump and skew-scattering are present, thus we have

$$\sigma^{sj,sk} \propto v^{sj}_l \delta^1 f^{sk}_l \sim (n_i V_0^2)^1. \tag{132}$$

For the nonlinear Hall conductivity, all three types of mixed terms are present, thus we have

$$\chi^{sj,sk} \propto v^{sj}_l \delta^2 f^{sk}_l \sim (n_i V_0^2)^0, \qquad \chi^{bd,sj} \propto \Omega_l \delta^1 f^{sj}_l \sim (n_i V_0^2)^0, \qquad \chi^{bd,sk} \propto \Omega_l \delta^1 f^{sk}_l \sim (n_i V_0^2)^0. \tag{133}$$

Therefore, the mixed terms are of higher orders in $n_i V_0^2$ than the leading contributions in both anomalous and nonlinear Hall response, thus can be neglected in the weak disorder limit.

SUPPLEMENTARY NOTE 4

Tilted Dirac fermion in 2D: As a minimal model, we first consider a tilt Dirac fermion in 2D space

$$\hat{\mathcal{H}}_t = tk_x + v(k_x\sigma_x + k_y\sigma_y) + m\sigma_z. \tag{134}$$

Without loss of generality, we assume the Dirac fermion is tilted along $\mathbf{x}$. The band structure reads

$$\varepsilon^{\pm}_{\mathbf{k}} = tk_x \pm \varepsilon^0_{\mathbf{k}}, \qquad \varepsilon^0_{\mathbf{k}} = \sqrt{v^2k^2 + m^2} \tag{135}$$

with $k^2 \equiv k_x^2 + k_y^2$. And the chiral basis vectors that diagonalize the Hamiltonian are

$$|\psi^{\pm}_{\mathbf{k}}\rangle = \frac{e^{i\mathbf{k}\cdot\mathbf{r}}}{\sqrt{\mathcal{V}}}|u^{\pm}_{\mathbf{k}}\rangle, \tag{136}$$

where $\mathcal{V}$ refers to the general volume and

$$|u^{+}_{\mathbf{k}}\rangle = \begin{pmatrix} \cos\frac{\theta}{2} \\ \sin\frac{\theta}{2}e^{i\phi} \end{pmatrix}, \qquad |u^{-}_{\mathbf{k}}\rangle = \begin{pmatrix} \sin\frac{\theta}{2} \\ -\cos\frac{\theta}{2}e^{i\phi} \end{pmatrix} \tag{137}$$

are the eigen states with

$$\cos\theta = \frac{m}{\sqrt{v^2k^2+m^2}}, \qquad \tan\phi = \frac{k_y}{k_x}. \tag{138}$$

Note that $\tan\phi = k_y/k_x$ indicating that $k_y = k\sin\phi$ and $k_x = k\cos\phi$. The velocities along two main axis are

$$v^x_{\pm} = \frac{1}{\hbar}\Big(t \pm \frac{v^2k_x}{\sqrt{v^2k^2+m^2}}\Big), \tag{139}$$

$$v^y_{\pm} = \pm\frac{1}{\hbar}\frac{v^2k_y}{\sqrt{v^2k^2+m^2}}. \tag{140}$$

And the Berry curvature for each band is

$$\Omega^{\pm}_{\mathbf{k}} = \mp\frac{mv^2}{2(v^2k^2+m^2)^{3/2}}. \tag{141}$$

Scattering rate

The scattering T-matrix is defined as

$$T_{ll'} = \langle\psi_l|\hat{V}_{imp}|\Psi_{l'}\rangle. \tag{142}$$

Here $|\Psi_l\rangle$ is the eigenstate of the full Hamiltonian $\hat{\mathcal{H}} = \hat{\mathcal{H}}_0 + \hat{V}_{imp}$ that satisfies the Lippman-Schwinger equation

$$|\Psi_l\rangle = |\psi_l\rangle + \frac{\hat{V}_{imp}}{\varepsilon_l - \hat{\mathcal{H}}_t + i\delta}|\Psi_l\rangle. \tag{143}$$

For weak disorder one can approximate the scattering state $|\Psi_l\rangle$ by a truncated series in powers of $V_{ll'} = \langle\psi_l|\hat{V}_{imp}|\psi_{l'}\rangle$ as

$$|\Psi_l\rangle \simeq |\psi_l\rangle + \sum_{l''}\frac{V_{l''l}}{\varepsilon_l - \varepsilon_{l''} + i\delta}|\psi_{l''}\rangle + \sum_{l''}\sum_{l'''}\frac{V_{l''l'''}V_{l'''l}}{(\varepsilon_l - \varepsilon_{l''} + i\delta)(\varepsilon_l - \varepsilon_{l'''} + i\delta)}|\psi_{l''}\rangle + \cdots. \tag{144}$$

Note that the scattering rate $\varpi_{ll'}$ between l and l' states is related to the T-matrix elements according to the so called golden rule, thus we have

$$\varpi_{ll'} = \frac{2\pi}{\hbar}|T_{ll'}|^2\delta(\varepsilon_l - \varepsilon_{l'}) \equiv \varpi^{(2)}_{ll'} + \varpi^{(3)}_{ll'} + \varpi^{(4)}_{ll'} + \cdots \tag{145}$$

with

$$\varpi_{ll'}^{(2)} = \frac{2\pi}{\hbar}\big\langle V_{ll'}^{*}V_{ll'}\big\rangle_{dis}\delta(\varepsilon_l - \varepsilon_{l'}), \tag{146}$$

$$\varpi_{ll'}^{(3)} = \frac{2\pi}{\hbar}\sum_{l''}\left(\frac{\big\langle V_{ll'}^{*}V_{ll''}V_{l''l'}\big\rangle_{dis}}{\varepsilon_{l'}-\varepsilon_{l''}+i\delta} + \frac{\big\langle V_{ll''}^{*}V_{l''l'}^{*}V_{ll'}\big\rangle_{dis}}{\varepsilon_{l'}-\varepsilon_{l''}-i\delta}\right)\delta(\varepsilon_l - \varepsilon_{l'}), \tag{147}$$

$$\begin{aligned}\varpi_{ll'}^{(4)} = \frac{2\pi}{\hbar}\sum_{l''}\sum_{l'''}\Bigg(&\frac{\big\langle V_{ll''}^{*}V_{l''l'}^{*}V_{ll'''}V_{l'''l'}\big\rangle_{dis}}{(\varepsilon_{l'}-\varepsilon_{l''}-i\delta)(\varepsilon_{l'}-\varepsilon_{l'''}+i\delta)} + \frac{\big\langle V_{ll'}^{*}V_{ll''}V_{l''l'''}V_{l'''l'}\big\rangle_{dis}}{(\varepsilon_{l'}-\varepsilon_{l''}+i\delta)(\varepsilon_{l'}-\varepsilon_{l'''}+i\delta)}\\ &+\frac{\big\langle V_{ll'}V_{ll''}^{*}V_{l''l'''}^{*}V_{l'''l'}^{*}\big\rangle_{dis}}{(\varepsilon_{l'}-\varepsilon_{l''}-i\delta)(\varepsilon_{l'}-\varepsilon_{l'''}-i\delta)}\Bigg)\delta(\varepsilon_l - \varepsilon_{l'}).\end{aligned} \tag{148}$$

For the tilted Dirac model, we consider the model of randomly located δ-function scatterers

$$\hat{V}_{imp}(\mathbf{r}) = \sum_i V_i\delta(\mathbf{r}-\mathbf{R}_i) \tag{149}$$

with the random distribution $\mathbf{R}_i$ and the disorder strength V_i satisfying $\langle V_i\rangle_{dis} = 0$, $\langle V_i^2\rangle_{dis} = V_0^2$, and $\langle V_i^3\rangle_{dis} = V_1^3$. In the eigenstate representation the operator elements of the disorder potential can be written in a general form as

$$\begin{aligned}V_{\mathbf{kk'}}^{\eta\eta'} &= V_{\mathbf{k},\mathbf{k'}}^{0}\frac{i^{(\eta+\eta')/2-1}}{4}\Big[\big(e^{i\theta/2}+\eta e^{-i\theta/2}\big)\big(e^{i\theta'/2}+\eta' e^{-i\theta'/2}\big) - \big(e^{i\theta/2}-\eta e^{-i\theta/2}\big)\big(e^{i\theta'/2}-\eta' e^{-i\theta'/2}\big)e^{i(\phi'-\phi)}\Big]\\ &= V_{\mathbf{k},\mathbf{k'}}^{0}\frac{i^{(\eta+\eta')/2-1}}{4}\Big\{\Big[e^{i(\theta+\theta')/2}+\eta\eta' e^{-i(\theta+\theta')/2}\Big]\Big[1-e^{i(\phi'-\phi)}\Big] + \Big[\eta e^{-i(\theta-\theta')/2}+\eta' e^{i(\theta-\theta')/2}\Big]\Big[1+e^{i(\phi'-\phi)}\Big]\Big\}\\ &\equiv \frac{V_{\mathbf{k},\mathbf{k'}}^{0}}{4}\Xi_{\varphi\varphi'}^{\eta\eta'},\end{aligned} \tag{150}$$

where $\eta,\eta' \in \pm$, $\varphi \in (\theta,\phi)$ represents the general angular index and $V_{\mathbf{k},\mathbf{k'}}^{0} \equiv \sum_i V_i e^{i(\mathbf{k'}-\mathbf{k})\mathbf{R}_i}$. For its complex conjugate, we have

$$\begin{aligned}(V_{\mathbf{kk'}}^{\eta\eta'})^{*} &= V_{\mathbf{k},\mathbf{k'}}^{0}\frac{i^{(\eta+\eta')/2-1}}{4}\Big[\big(e^{i\theta/2}+\eta e^{-i\theta/2}\big)\big(e^{i\theta'/2}+\eta' e^{-i\theta'/2}\big) - \big(e^{i\theta/2}-\eta e^{-i\theta/2}\big)\big(e^{i\theta'/2}-\eta' e^{-i\theta'/2}\big)e^{i(\phi-\phi')}\Big]\\ &= V_{\mathbf{k},\mathbf{k'}}^{0}\frac{i^{(\eta+\eta')/2-1}}{4}\Big\{\Big[e^{i(\theta+\theta')/2}+\eta\eta' e^{-i(\theta+\theta')/2}\Big]\Big[1-e^{i(\phi-\phi')}\Big] + \Big[\eta e^{-i(\theta-\theta')/2}+\eta' e^{i(\theta-\theta')/2}\Big]\Big[1+e^{i(\phi-\phi')}\Big]\Big\}\\ &\equiv \frac{V_{\mathbf{k},\mathbf{k'}}^{0}}{4}\tilde{\Xi}_{\varphi\varphi'}^{\eta\eta'}.\end{aligned} \tag{151}$$

It is obvious that we have $\tilde{\Xi}_{\varphi\varphi'}^{\eta\eta'} = (\Xi_{\varphi\varphi'}^{\eta\eta'})^{*}$.

Second-order scattering rate

Assume that the Fermi level lies in the upper bands, thus we have

$$\begin{aligned}\varpi_{\mathbf{kk'}}^{(2)} &= \frac{2\pi}{\hbar}\big\langle (V_{\mathbf{kk'}}^{++})^{*}V_{\mathbf{kk'}}^{++}\big\rangle_{dis}\delta(\varepsilon_{\mathbf{k}}^{+}-\varepsilon_{\mathbf{k'}}^{+})\\ &= \frac{\pi n_i V_0^2}{\hbar}\big[1+\cos\theta\cos\theta'+\sin\theta\sin\theta'\cos(\phi'-\phi)\big]\delta(\varepsilon_{\mathbf{k}}^{+}-\varepsilon_{\mathbf{k'}}^{+}),\end{aligned} \tag{152}$$

where we've used

$$\big\langle (V_{\mathbf{kk'}}^{0})^{*}V_{\mathbf{kk'}}^{0}\big\rangle_{dis} = \big\langle \sum_{ij} V_iV_j e^{-i(\mathbf{k'}-\mathbf{k})\mathbf{R}_i}e^{i(\mathbf{k'}-\mathbf{k})\mathbf{R}_j}\big\rangle_{dis} \simeq n_iV_0^2 \tag{153}$$

with n_i refers to the concentration of impurities.

Third-order scattering rate

The third-order scattering term for the upper band is

$$\varpi^{(3)}_{\mathbf{kk}'} = \frac{2\pi}{\hbar}\int [dk'']\left(\frac{\langle V^{++}_{\mathbf{kk}'}(V^{+\eta}_{\mathbf{kk}''})^*(V^{\eta+}_{\mathbf{k}''\mathbf{k}'})^*\rangle_{dis}}{\varepsilon^+_{\mathbf{k}'}-\varepsilon^{\eta}_{\mathbf{k}''}-i\delta}+c.c.\right)\delta(\varepsilon^+_{\mathbf{k}}-\varepsilon^+_{\mathbf{k}'}) \equiv \omega^{(3s)}_{\mathbf{kk}'}+\omega^{(3a)}_{\mathbf{kk}'}, \tag{154}$$

where $\omega^{(3s)}_{\mathbf{kk}'}$ and $\omega^{(3a)}_{\mathbf{kk}'}$ represent the symmetric and antisymmetric parts of the third-order scattering rate. The symmetric part is not essential since it only renormalizes the second-order result. The antisymmetric part can be written as

$$\varpi^{(3a)}_{\mathbf{kk}'} = \frac{\pi}{\hbar}\int [dk'']\frac{n_iV_1^3}{2^6}\left(\frac{\Xi^{++}_{\varphi\varphi'}\tilde{\Xi}^{+\eta}_{\varphi\varphi''}\tilde{\Xi}^{\eta+}_{\varphi''\varphi'}-\Xi^{+\eta}_{\varphi\varphi''}\tilde{\Xi}^{\eta+}_{\varphi''\varphi'}\tilde{\Xi}^{++}_{\varphi'\varphi}}{\varepsilon^+_{\mathbf{k}'}-\varepsilon^{\eta}_{\mathbf{k}''}-i\delta}+c.c.\right)\delta(\varepsilon^+_{\mathbf{k}}-\varepsilon^+_{\mathbf{k}'}), \tag{155}$$

where we've used

$$\langle V^0_{\mathbf{kk}'}(V^0_{\mathbf{kk}''})^*(V^0_{\mathbf{k}''\mathbf{k}'})^*\rangle_{dis} \simeq n_iV_1^3. \tag{156}$$

After some tedious calculation, the numerator can be simplified as

$$\begin{aligned}\mathrm{Nu} &\equiv \Xi^{++}_{\varphi\varphi'}\tilde{\Xi}^{+\eta}_{\varphi\varphi''}\tilde{\Xi}^{\eta+}_{\varphi''\varphi'}-\Xi^{+\eta}_{\varphi\varphi''}\tilde{\Xi}^{\eta+}_{\varphi''\varphi'}\tilde{\Xi}^{++}_{\varphi'\varphi}\\ &= -i^{\eta}2^5\left[\sin\theta\sin\theta'\cos\theta''\sin(\phi-\phi')+\sin\theta'\sin\theta''\cos\theta\sin(\phi'-\phi'')+\sin\theta\sin\theta''\cos\theta'\sin(\phi''-\phi)\right].\end{aligned} \tag{157}$$

Thus, for the type-I Dirac fermion ($t < v$) with Fermi level in the upper band, we have

$$\begin{aligned}\varpi^{(3a)}_{\mathbf{kk}'} = \frac{n_iV_1^3}{4\hbar}\int_0^{\infty}k''dk''\int_0^{2\pi}d\phi''\Big[&\sin\theta\sin\theta'\cos\theta''\sin(\phi-\phi')+\sin\theta'\sin\theta''\cos\theta\sin(\phi'-\phi'')\\ &+\sin\theta\sin\theta''\cos\theta'\sin(\phi''-\phi)\Big]\delta(\varepsilon^+_{\mathbf{k}'}-\varepsilon^+_{\mathbf{k}''})\delta(\varepsilon^+_{\mathbf{k}}-\varepsilon^+_{\mathbf{k}'}).\end{aligned} \tag{158}$$

Fourth-order scattering rate

The forth order scattering terms for the upper band are

$$\begin{aligned}\varpi^{(4)}_{\mathbf{kk}'} = \frac{2\pi}{\hbar}\int[dk'']\int[dk''']\Bigg[&\frac{\langle (V^{+\eta}_{\mathbf{kk}''})^*(V^{\eta+}_{\mathbf{k}''\mathbf{k}'})^*V^{+\eta'}_{\mathbf{kk}'''}V^{\eta'+}_{\mathbf{k}'''\mathbf{k}'}\rangle_{dis}}{(\varepsilon^+_{\mathbf{k}'}-\varepsilon^{\eta}_{\mathbf{k}''}-i\delta)(\varepsilon^+_{\mathbf{k}'}-\varepsilon^{\eta'}_{\mathbf{k}'''}+i\delta)}+\frac{\langle (V^{++}_{\mathbf{kk}'})^*V^{+\eta}_{\mathbf{kk}''}V^{\eta\eta'}_{\mathbf{k}''\mathbf{k}'''}V^{\eta'+}_{\mathbf{k}'''\mathbf{k}'}\rangle_{dis}}{(\varepsilon^+_{\mathbf{k}'}-\varepsilon^{\eta}_{\mathbf{k}''}+i\delta)(\varepsilon^+_{\mathbf{k}'}-\varepsilon^{\eta'}_{\mathbf{k}'''}+i\delta)}\\ &+\frac{\langle V^{++}_{\mathbf{kk}'}(V^{+\eta}_{\mathbf{kk}''})^*(V^{\eta\eta'}_{\mathbf{k}''\mathbf{k}'''})^*(V^{\eta'+}_{\mathbf{k}'''\mathbf{k}'})^*\rangle_{dis}}{(\varepsilon^+_{\mathbf{k}'}-\varepsilon^{\eta}_{\mathbf{k}''}-i\delta)(\varepsilon^+_{\mathbf{k}'}-\varepsilon^{\eta'}_{\mathbf{k}'''}-i\delta)}\Bigg]\delta(\varepsilon^+_{\mathbf{k}}-\varepsilon^+_{\mathbf{k}'}).\end{aligned} \tag{159}$$

Note that we did not take into account the fourth-order correlations, thus the disorder average in the above equality have to be decomposed into the products of the second-order correlations.

According to the Wick's theorem, we have

$$\begin{aligned}\langle (V^{+\eta}_{\mathbf{kk}''})^*(V^{\eta+}_{\mathbf{k}''\mathbf{k}'})^*V^{+\eta'}_{\mathbf{kk}'''}V^{\eta'+}_{\mathbf{k}'''\mathbf{k}'}\rangle_{dis} &= \langle (V^{+\eta}_{\mathbf{kk}''})^*(V^{\eta+}_{\mathbf{k}''\mathbf{k}'})^*\rangle_{dis}\langle V^{+\eta'}_{\mathbf{kk}'''}V^{\eta'+}_{\mathbf{k}'''\mathbf{k}'}\rangle_{dis}\\ &+\langle (V^{+\eta}_{\mathbf{kk}''})^*V^{+\eta'}_{\mathbf{kk}'''}\rangle_{dis}\langle V^{\eta'+}_{\mathbf{k}'''\mathbf{k}'}(V^{\eta+}_{\mathbf{k}''\mathbf{k}'})^*\rangle_{dis}\\ &+\langle (V^{+\eta}_{\mathbf{kk}''})^*V^{\eta'+}_{\mathbf{k}'''\mathbf{k}'}\rangle_{dis}\langle V^{+\eta'}_{\mathbf{kk}'''}(V^{\eta+}_{\mathbf{k}''\mathbf{k}'})^*\rangle_{dis},\end{aligned} \tag{160}$$

which correspond to the diagrams Fig. 1 (a)-(c).

$$\begin{aligned}\langle (V^{++}_{\mathbf{kk}'})^*V^{+\eta}_{\mathbf{kk}''}V^{\eta\eta'}_{\mathbf{k}''\mathbf{k}'''}V^{\eta'+}_{\mathbf{k}'''\mathbf{k}'}\rangle_{dis} &= \langle (V^{++}_{\mathbf{kk}'})^*V^{+\eta}_{\mathbf{kk}''}\rangle_{dis}\langle V^{\eta\eta'}_{\mathbf{k}''\mathbf{k}'''}V^{\eta'+}_{\mathbf{k}'''\mathbf{k}'}\rangle_{dis}\\ &+\langle (V^{++}_{\mathbf{kk}'})^*V^{\eta'+}_{\mathbf{k}'''\mathbf{k}'}\rangle_{dis}\langle V^{+\eta}_{\mathbf{kk}''}V^{\eta\eta'}_{\mathbf{k}''\mathbf{k}'''}\rangle_{dis}\\ &+\langle (V^{++}_{\mathbf{kk}'})^*V^{\eta\eta'}_{\mathbf{k}''\mathbf{k}'''}\rangle_{dis}\langle V^{+\eta}_{\mathbf{kk}''}V^{\eta'+}_{\mathbf{k}'''\mathbf{k}'}\rangle_{dis},\end{aligned} \tag{161}$$

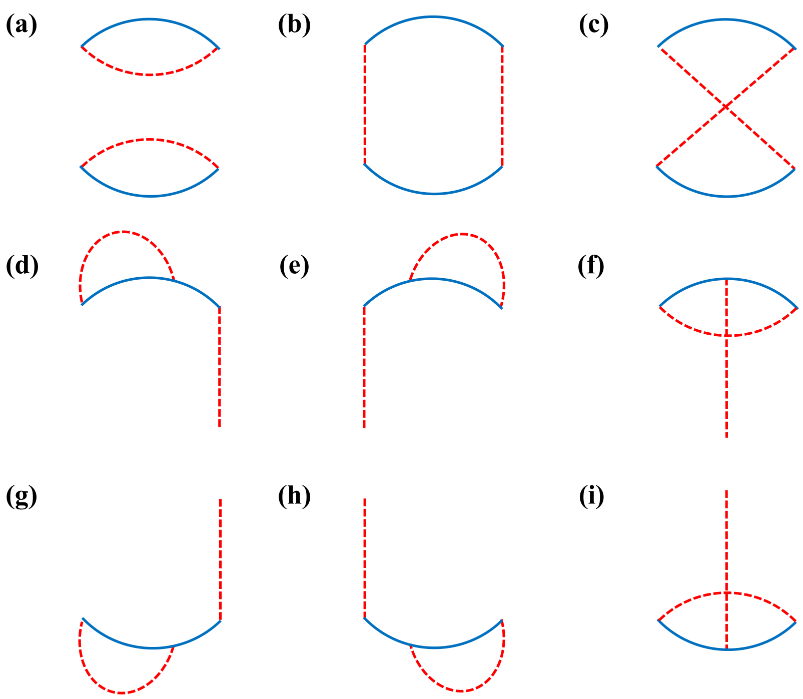

FIG. 1. Feynmann diagrams that correspond to the fourth-order scattering terms.

which correspond to the diagrams Fig. 1 (d)-(f). And

$$\begin{aligned}\big\langle V_{\mathbf{kk}'}^{++}(V_{\mathbf{kk}''}^{+\eta})^*(V_{\mathbf{k}''\mathbf{k}'''}^{\eta\eta'})^*(V_{\mathbf{k}'''\mathbf{k}'}^{\eta'+})^*\big\rangle_{dis} =& \big\langle V_{\mathbf{kk}'}^{++}(V_{\mathbf{kk}''}^{+\eta})^*\big\rangle_{dis}\big\langle(V_{\mathbf{k}''\mathbf{k}'''}^{\eta\eta'})^*(V_{\mathbf{k}'''\mathbf{k}'}^{\eta'+})^*\big\rangle_{dis}\\ &+\big\langle V_{\mathbf{kk}'}^{++}(V_{\mathbf{k}'''\mathbf{k}'}^{\eta'+})^*\big\rangle_{dis}\big\langle(V_{\mathbf{kk}''}^{+\eta})^*(V_{\mathbf{k}''\mathbf{k}'''}^{\eta\eta'})^*\big\rangle_{dis}\\ &+\big\langle V_{\mathbf{kk}'}^{++}(V_{\mathbf{k}''\mathbf{k}'''}^{\eta\eta'})^*\big\rangle_{dis}\big\langle(V_{\mathbf{kk}''}^{+\eta})^*(V_{\mathbf{k}'''\mathbf{k}'}^{\eta'+})^*\big\rangle_{dis},\end{aligned} \tag{162}$$

which correspond to the diagrams Fig. 1 (g)-(i). Here Fig. 1 (a) only corresponds to the quantitative correction to the second-order scattering rate, which can be neglect. And the contributions Fig. 1 (c), (f) and (i) are beyond the noncrossing approximation (NCA). As a result, within NCA, the only contributions to the fourth-order scattering rate are Fig. 1 (b), (d), (e), (g) and (h), which can be regrouped up into three independent terms as

$$\varpi_{\mathbf{kk}'}^{(4)} = \varpi_{\mathbf{kk}'}^{(4-1)} + \varpi_{\mathbf{kk}'}^{(4-2)} + \varpi_{\mathbf{kk}'}^{(4-3)} \tag{163}$$

with

$$\begin{aligned}\varpi_{\mathbf{kk}'}^{(4-1)} &= \frac{2\pi}{\hbar}\int[dk'']\int[dk''']\left[\frac{\big\langle(V_{\mathbf{kk}''}^{+\eta})^*V_{\mathbf{kk}'''}^{+\eta'}\big\rangle_{dis}\big\langle V_{\mathbf{k}'''\mathbf{k}'}^{\eta'+}(V_{\mathbf{k}''\mathbf{k}'}^{\eta+})^*\big\rangle_{dis}}{(\varepsilon_{\mathbf{k}'}^{+}-\varepsilon_{\mathbf{k}''}^{\eta}-i\delta)(\varepsilon_{\mathbf{k}'}^{+}-\varepsilon_{\mathbf{k}'''}^{\eta'}+i\delta)}\right]\delta(\varepsilon_{\mathbf{k}}^{+}-\varepsilon_{\mathbf{k}'}^{+})\\ &= \frac{n_i^2V_0^4}{2^9\pi\hbar}\int_0^\infty k''dk''\int_0^{2\pi}d\phi''\frac{\tilde{\Xi}_{\varphi\varphi''}^{+\eta}\Xi_{\varphi\varphi''}^{+\eta'}\Xi_{\varphi''\varphi'}^{\eta'+}\tilde{\Xi}_{\varphi''\varphi'}^{\eta+}}{(\varepsilon_{\mathbf{k}'}^{+}-\varepsilon_{\mathbf{k}''}^{\eta}-i\delta)(\varepsilon_{\mathbf{k}'}^{+}-\varepsilon_{\mathbf{k}''}^{\eta'}+i\delta)}\delta(\varepsilon_{\mathbf{k}}^{+}-\varepsilon_{\mathbf{k}'}^{+}),\end{aligned} \tag{164}$$

$$\begin{aligned}\varpi_{\mathbf{kk}'}^{(4-2)} &= \frac{2\pi}{\hbar}\int[dk'']\int[dk''']\left[\frac{\big\langle(V_{\mathbf{kk}'}^{++})^*V_{\mathbf{kk}''}^{+\eta}\big\rangle_{dis}\big\langle V_{\mathbf{k}''\mathbf{k}'''}^{\eta\eta'}V_{\mathbf{k}'''\mathbf{k}'}^{\eta'+}\big\rangle_{dis}}{(\varepsilon_{\mathbf{k}'}^{+}-\varepsilon_{\mathbf{k}''}^{\eta}+i\delta)(\varepsilon_{\mathbf{k}'}^{+}-\varepsilon_{\mathbf{k}'''}^{\eta'}+i\delta)}+c.c.\right]\delta(\varepsilon_{\mathbf{k}}^{+}-\varepsilon_{\mathbf{k}'}^{+})\\ &= \frac{n_i^2V_0^4}{2^9\pi\hbar}\int_0^\infty k''dk''\int_0^{2\pi}d\phi''\Big[\frac{\tilde{\Xi}_{\varphi\varphi'}^{++}\Xi_{\varphi\varphi'}^{+\eta}\Xi_{\varphi'\varphi''}^{\eta\eta'}\Xi_{\varphi''\varphi'}^{\eta'+}}{(\varepsilon_{\mathbf{k}'}^{+}-\varepsilon_{\mathbf{k}'}^{\eta}+i\delta)(\varepsilon_{\mathbf{k}'}^{+}-\varepsilon_{\mathbf{k}''}^{\eta'}+i\delta)}+c.c.\Big]\delta(\varepsilon_{\mathbf{k}}^{+}-\varepsilon_{\mathbf{k}'}^{+}),\end{aligned} \tag{165}$$

$$\begin{aligned}\varpi_{\mathbf{kk}'}^{(4-3)} &= \frac{2\pi}{\hbar}\int[dk'']\int[dk''']\left[\frac{\big\langle(V_{\mathbf{kk}'}^{++})^*V_{\mathbf{k}'''\mathbf{k}'}^{\eta'+}\big\rangle_{dis}\big\langle V_{\mathbf{kk}''}^{+\eta}V_{\mathbf{k}''\mathbf{k}'''}^{\eta\eta'}\big\rangle_{dis}}{(\varepsilon_{\mathbf{k}'}^{+}-\varepsilon_{\mathbf{k}''}^{\eta}+i\delta)(\varepsilon_{\mathbf{k}'}^{+}-\varepsilon_{\mathbf{k}'''}^{\eta'}+i\delta)}+c.c.\right]\delta(\varepsilon_{\mathbf{k}}^{+}-\varepsilon_{\mathbf{k}'}^{+})\\ &= \frac{n_i^2V_0^4}{2^9\pi\hbar}\int_0^\infty k''dk''\int_0^{2\pi}d\phi''\Big[\frac{\tilde{\Xi}_{\varphi\varphi'}^{++}\Xi_{\varphi\varphi'}^{\eta'+}\Xi_{\varphi\varphi''}^{+\eta}\Xi_{\varphi''\varphi}^{\eta\eta'}}{(\varepsilon_{\mathbf{k}'}^{+}-\varepsilon_{\mathbf{k}''}^{\eta}+i\delta)(\varepsilon_{\mathbf{k}'}^{+}-\varepsilon_{\mathbf{k}}^{\eta'}+i\delta)}+c.c.\Big]\delta(\varepsilon_{\mathbf{k}}^{+}-\varepsilon_{\mathbf{k}'}^{+})..\end{aligned} \tag{166}$$

The symmetric parts of these contributions are irrelevant with the skew-scattering, thus we only consider their antisymmetric parts. Note that $\varpi^{(4a-n)}_{\mathbf{kk'}} \equiv (\varpi^{(4-n)}_{\mathbf{kk'}} - \varpi^{(4-n)}_{\mathbf{k'k}})/2$, thus we have

$$\varpi^{(4a-1)}_{\mathbf{kk'}} = \frac{n_i^2 V_0^4}{2^{10}\pi\hbar}\int_0^\infty k''dk''\int_0^{2\pi} d\phi'' \frac{\tilde{\Xi}^{+\eta}_{\varphi\varphi''}\Xi^{+\eta'}_{\varphi\varphi''}\Xi^{\eta'+}_{\varphi''\varphi'}\tilde{\Xi}^{\eta+}_{\varphi''\varphi'} - \tilde{\Xi}^{+\eta}_{\varphi'\varphi''}\Xi^{+\eta'}_{\varphi'\varphi''}\Xi^{\eta'+}_{\varphi''\varphi}\tilde{\Xi}^{\eta+}_{\varphi''\varphi}}{(\varepsilon^+_{\mathbf{k'}} - \varepsilon^\eta_{\mathbf{k''}} - i\delta)(\varepsilon^+_{\mathbf{k'}} - \varepsilon^{\eta'}_{\mathbf{k''}} + i\delta)}\delta(\varepsilon^+_{\mathbf{k}} - \varepsilon^+_{\mathbf{k'}}), \tag{167}$$

$$\begin{aligned}\varpi^{(4a-2)}_{\mathbf{kk'}} = \frac{n_i^2 V_0^4}{2^{10}\pi\hbar}\int_0^\infty k''dk''\int_0^{2\pi} d\phi'' \Big\{\Big[\frac{\tilde{\Xi}^{++}_{\varphi\varphi'}\Xi^{+\eta}_{\varphi\varphi'}\Xi^{\eta\eta'}_{\varphi'\varphi''}\Xi^{\eta'+}_{\varphi''\varphi'}}{(\varepsilon^+_{\mathbf{k'}} - \varepsilon^\eta_{\mathbf{k'}} + i\delta)(\varepsilon^+_{\mathbf{k'}} - \varepsilon^{\eta'}_{\mathbf{k''}} + i\delta)} + c.c.\Big] \\ -\Big[\frac{\tilde{\Xi}^{++}_{\varphi'\varphi}\Xi^{+\eta}_{\varphi'\varphi}\Xi^{\eta\eta'}_{\varphi\varphi''}\Xi^{\eta'+}_{\varphi''\varphi}}{(\varepsilon^+_{\mathbf{k'}} - \varepsilon^\eta_{\mathbf{k}} + i\delta)(\varepsilon^+_{\mathbf{k'}} - \varepsilon^{\eta'}_{\mathbf{k''}} + i\delta)} + c.c.\Big]\Big\}\delta(\varepsilon^+_{\mathbf{k}} - \varepsilon^+_{\mathbf{k'}}),\end{aligned} \tag{168}$$

$$\begin{aligned}\varpi^{(4a-3)}_{\mathbf{kk'}} = \frac{n_i^2 V_0^4}{2^{10}\pi\hbar}\int_0^\infty k''dk''\int_0^{2\pi} d\phi'' \Big\{\Big[\frac{\tilde{\Xi}^{++}_{\varphi\varphi'}\Xi^{\eta'+}_{\varphi\varphi'}\Xi^{+\eta}_{\varphi\varphi''}\Xi^{\eta\eta'}_{\varphi''\varphi}}{(\varepsilon^+_{\mathbf{k'}} - \varepsilon^\eta_{\mathbf{k''}} + i\delta)(\varepsilon^+_{\mathbf{k'}} - \varepsilon^{\eta'}_{\mathbf{k}} + i\delta)} + c.c.\Big] \\ -\Big[\frac{\tilde{\Xi}^{++}_{\varphi'\varphi}\Xi^{\eta'+}_{\varphi'\varphi}\Xi^{+\eta}_{\varphi'\varphi''}\Xi^{\eta\eta'}_{\varphi''\varphi'}}{(\varepsilon^+_{\mathbf{k'}} - \varepsilon^\eta_{\mathbf{k''}} + i\delta)(\varepsilon^+_{\mathbf{k'}} - \varepsilon^{\eta'}_{\mathbf{k'}} + i\delta)} + c.c.\Big]\Big\}\delta(\varepsilon^+_{\mathbf{k}} - \varepsilon^+_{\mathbf{k'}}).\end{aligned} \tag{169}$$

Here the last two contributions can be regrouped up into a more compact form as

$$\varpi^{(4a-2')}_{\mathbf{kk'}} = \frac{n_i^2 V_0^4}{2^9\pi\hbar}\int_0^\infty k''dk''\int_0^{2\pi} d\phi'' \mathrm{Re}\Big[\frac{\tilde{\Xi}^{++}_{\varphi\varphi'}\Xi^{+\eta}_{\varphi\varphi'}\Xi^{\eta\eta'}_{\varphi'\varphi''}\Xi^{\eta'+}_{\varphi''\varphi'} - \tilde{\Xi}^{++}_{\varphi'\varphi}\Xi^{\eta+}_{\varphi'\varphi}\Xi^{+\eta'}_{\varphi'\varphi''}\Xi^{\eta'\eta}_{\varphi''\varphi'}}{(\varepsilon^+_{\mathbf{k'}} - \varepsilon^\eta_{\mathbf{k'}} + i\delta)(\varepsilon^+_{\mathbf{k'}} - \varepsilon^{\eta'}_{\mathbf{k''}} + i\delta)}\Big]\delta(\varepsilon^+_{\mathbf{k}} - \varepsilon^+_{\mathbf{k'}}), \tag{170}$$

$$\varpi^{(4a-3')}_{\mathbf{kk'}} = \frac{n_i^2 V_0^4}{2^9\pi\hbar}\int_0^\infty k''dk''\int_0^{2\pi} d\phi'' \mathrm{Re}\Big[\frac{\tilde{\Xi}^{++}_{\varphi\varphi'}\Xi^{\eta'+}_{\varphi\varphi'}\Xi^{+\eta}_{\varphi\varphi''}\Xi^{\eta\eta'}_{\varphi''\varphi} - \tilde{\Xi}^{++}_{\varphi'\varphi}\Xi^{+\eta'}_{\varphi'\varphi}\Xi^{\eta'\eta}_{\varphi\varphi''}\Xi^{\eta+}_{\varphi''\varphi}}{(\varepsilon^+_{\mathbf{k'}} - \varepsilon^\eta_{\mathbf{k''}} + i\delta)(\varepsilon^+_{\mathbf{k'}} - \varepsilon^{\eta'}_{\mathbf{k}} + i\delta)}\Big]\delta(\varepsilon^+_{\mathbf{k}} - \varepsilon^+_{\mathbf{k'}}). \tag{171}$$

It is obvious that $\varpi^{(4a-2)}_{\mathbf{kk'}} + \varpi^{(4a-3)}_{\mathbf{kk'}} = \varpi^{(4a-2')}_{\mathbf{kk'}} + \varpi^{(4a-3')}_{\mathbf{kk'}}$. Note that the nominators are all pure imaginary as

$$\begin{aligned}\mathrm{Nu1} &\equiv \tilde{\Xi}^{+\eta}_{\varphi\varphi''}\Xi^{+\eta'}_{\varphi\varphi''}\Xi^{\eta'+}_{\varphi''\varphi'}\tilde{\Xi}^{\eta+}_{\varphi''\varphi'} - \tilde{\Xi}^{+\eta}_{\varphi'\varphi''}\Xi^{+\eta'}_{\varphi'\varphi''}\Xi^{\eta'+}_{\varphi''\varphi}\tilde{\Xi}^{\eta+}_{\varphi''\varphi} \\ &= i2^6(-1)^{(\eta+\eta')/2}(\eta-\eta')\big[\sin\theta\sin\theta'\cos\theta''\sin(\phi'-\phi) + \sin\theta'\sin\theta''\cos\theta\sin(\phi''-\phi') \\ &\quad + \sin\theta\sin\theta''\cos\theta'\sin(\phi-\phi'')\big],\end{aligned} \tag{172}$$

$$\begin{aligned}\mathrm{Nu2} &\equiv \tilde{\Xi}^{++}_{\varphi\varphi'}\Xi^{+\eta}_{\varphi\varphi'}\Xi^{\eta\eta'}_{\varphi'\varphi''}\Xi^{\eta'+}_{\varphi''\varphi'} - \tilde{\Xi}^{++}_{\varphi'\varphi}\Xi^{\eta+}_{\varphi'\varphi}\Xi^{+\eta'}_{\varphi'\varphi''}\Xi^{\eta'\eta}_{\varphi''\varphi'} \\ &= i2^6(-1)^{(\eta+\eta')/2}(\eta-1)\big[\sin\theta\sin\theta'\cos\theta''\sin(\phi'-\phi) + \sin\theta'\sin\theta''\cos\theta\sin(\phi''-\phi') \\ &\quad + \sin\theta\sin\theta''\cos\theta'\sin(\phi-\phi'')\big],\end{aligned} \tag{173}$$

$$\begin{aligned}\mathrm{Nu3} &\equiv \tilde{\Xi}^{++}_{\varphi\varphi'}\Xi^{\eta'+}_{\varphi\varphi'}\Xi^{+\eta}_{\varphi\varphi''}\Xi^{\eta\eta'}_{\varphi''\varphi} - \tilde{\Xi}^{++}_{\varphi'\varphi}\Xi^{+\eta'}_{\varphi'\varphi}\Xi^{\eta'\eta}_{\varphi\varphi''}\Xi^{\eta+}_{\varphi''\varphi} \\ &= i2^6(-1)^{(\eta+\eta')/2}(\eta'-1)\big[\sin\theta\sin\theta'\cos\theta''\sin(\phi'-\phi) + \sin\theta'\sin\theta''\cos\theta\sin(\phi''-\phi') \\ &\quad + \sin\theta\sin\theta''\cos\theta'\sin(\phi-\phi'')\big].\end{aligned} \tag{174}$$

Thus, we obtain that

$$\begin{aligned}\varpi^{(4a-1)}_{\mathbf{kk'}} = \frac{n_i^2 V_0^4}{2^3\hbar m}\int_0^\infty k''dk''\int_0^{2\pi} d\phi''\cos\theta''\big[\sin\theta\sin\theta'\cos\theta''\sin(\phi-\phi') + \sin\theta'\sin\theta''\cos\theta\sin(\phi'-\phi'') \\ + \sin\theta\sin\theta''\cos\theta'\sin(\phi''-\phi)\big]\delta\big(\varepsilon^+_{\mathbf{k'}} - \varepsilon^+_{\mathbf{k''}}\big)\delta(\varepsilon^+_{\mathbf{k}} - \varepsilon^+_{\mathbf{k'}}),\end{aligned} \tag{175}$$

$$\begin{aligned}\varpi^{(4a-2')}_{\mathbf{kk'}} = \frac{n_i^2 V_0^4}{2^3\hbar m}\int_0^\infty k''dk''\int_0^{2\pi} d\phi''\cos\theta'\big[\sin\theta\sin\theta'\cos\theta''\sin(\phi-\phi') + \sin\theta'\sin\theta''\cos\theta\sin(\phi'-\phi'') \\ + \sin\theta\sin\theta''\cos\theta'\sin(\phi''-\phi)\big]\delta\big(\varepsilon^+_{\mathbf{k'}} - \varepsilon^+_{\mathbf{k''}}\big)\delta(\varepsilon^+_{\mathbf{k}} - \varepsilon^+_{\mathbf{k'}}),\end{aligned} \tag{176}$$

$$\begin{aligned}\varpi^{(4a-3')}_{\mathbf{kk'}} = \frac{n_i^2 V_0^4}{2^3\hbar m}\int_0^\infty k''dk''\int_0^{2\pi} d\phi''\cos\theta\big[\sin\theta\sin\theta'\cos\theta''\sin(\phi-\phi') + \sin\theta'\sin\theta''\cos\theta\sin(\phi'-\phi'') \\ + \sin\theta\sin\theta''\cos\theta'\sin(\phi''-\phi)\big]\delta\big(\varepsilon^+_{\mathbf{k'}} - \varepsilon^+_{\mathbf{k''}}\big)\delta(\varepsilon^+_{\mathbf{k}} - \varepsilon^+_{\mathbf{k'}}).\end{aligned} \tag{177}$$

Thus, the antisymmetric fourth-order scattering rate within NCA reads

$$\begin{aligned}\varpi^{(4a)}_{\mathbf{k}\mathbf{k}'} &= \varpi^{(4a-1)}_{\mathbf{k}\mathbf{k}'} + \varpi^{(4a-2')}_{\mathbf{k}\mathbf{k}'} + \varpi^{(4a-3')}_{\mathbf{k}\mathbf{k}'} \\ &= \frac{n_i^2 V_0^4}{8\hbar m}\int_0^\infty k''dk'' \int_0^{2\pi} d\phi''(\cos\theta+\cos\theta'+\cos\theta'')\big[\sin\theta\sin\theta'\cos\theta''\sin(\phi-\phi') \\ &\quad +\sin\theta'\sin\theta''\cos\theta\sin(\phi'-\phi'')+\sin\theta\sin\theta''\cos\theta'\sin(\phi''-\phi)\big]\delta\big(\varepsilon^+_{\mathbf{k}'}-\varepsilon^+_{\mathbf{k}''}\big)\delta(\varepsilon^+_{\mathbf{k}}-\varepsilon^+_{\mathbf{k}'}). \end{aligned} \tag{178}$$

Coordinate shift

Assume that the Fermi level lies in the upper band, the coordinate shift of the tilted 2D Dirac model with a δ-correlated spin independent random potential is given by

$$\delta\mathbf{r}_{\mathbf{k}\mathbf{k}'} = \Big\langle u^+_{\mathbf{k}}\Big|i\frac{\partial u^+_{\mathbf{k}}}{\partial\mathbf{k}}\Big\rangle - \Big\langle u^+_{\mathbf{k}'}\Big|i\frac{\partial u^+_{\mathbf{k}'}}{\partial\mathbf{k}'}\Big\rangle - \Big(\frac{\partial}{\partial\mathbf{k}}+\frac{\partial}{\partial\mathbf{k}'}\Big)\arg\big[\langle u^+_{\mathbf{k}}|u^+_{\mathbf{k}'}\rangle\big]. \tag{179}$$

In components, we have

$$\Big\langle u^+_{\mathbf{k}}\Big|\frac{\partial}{\partial k}\Big|u^+_{\mathbf{k}}\Big\rangle = \begin{pmatrix}\cos\frac{\theta}{2}, & \sin\frac{\theta}{2}e^{-i\phi}\end{pmatrix}\frac{\partial}{\partial k}\begin{pmatrix}\cos\frac{\theta}{2}\\ \sin\frac{\theta}{2}e^{i\phi}\end{pmatrix} = 0, \tag{180}$$

$$\Big\langle u^+_{\mathbf{k}}\Big|\frac{\partial}{\partial \phi}\Big|u^+_{\mathbf{k}}\Big\rangle = \begin{pmatrix}\cos\frac{\theta}{2}, & \sin\frac{\theta}{2}e^{-i\phi}\end{pmatrix}\frac{\partial}{\partial \phi}\begin{pmatrix}\cos\frac{\theta}{2}\\ \sin\frac{\theta}{2}e^{i\phi}\end{pmatrix} = i\frac{1-\cos\theta}{2}, \tag{181}$$

thus we obtain that

$$i\Big\langle u^+_{\mathbf{k}}\Big|\frac{\partial}{\partial k_x}\Big|u^+_{\mathbf{k}}\Big\rangle = i\cos\phi\Big\langle u^+_{\mathbf{k}}\Big|\frac{\partial}{\partial k}\Big|u^+_{\mathbf{k}}\Big\rangle - i\frac{\sin\phi}{k}\Big\langle u^+_{\mathbf{k}}\Big|\frac{\partial}{\partial \phi}\Big|u^+_{\mathbf{k}}\Big\rangle = \frac{1-\cos\theta}{2k}\sin\phi, \tag{182}$$

$$i\Big\langle u^+_{\mathbf{k}}\Big|\frac{\partial}{\partial k_y}\Big|u^+_{\mathbf{k}}\Big\rangle = i\sin\phi\Big\langle u^+_{\mathbf{k}}\Big|\frac{\partial}{\partial k}\Big|u^+_{\mathbf{k}}\Big\rangle + i\frac{\cos\phi}{k}\Big\langle u^+_{\mathbf{k}}\Big|\frac{\partial}{\partial \phi}\Big|u^+_{\mathbf{k}}\Big\rangle = \frac{\cos\theta-1}{2k}\cos\phi. \tag{183}$$

On the other hand, we have

$$\arg\big[\langle u^+_{\mathbf{k}}|u^+_{\mathbf{k}'}\rangle\big] = -i\ln\frac{\langle u^+_{\mathbf{k}}|u^+_{\mathbf{k}'}\rangle}{|\langle u^+_{\mathbf{k}}|u^+_{\mathbf{k}'}\rangle|} \tag{184}$$

with

$$\langle u^+_{\mathbf{k}}|u^+_{\mathbf{k}'}\rangle = \cos\frac{\theta}{2}\cos\frac{\theta'}{2}+\sin\frac{\theta}{2}\sin\frac{\theta'}{2}e^{i(\phi'-\phi)}, \tag{185}$$

$$\big|\langle u^+_{\mathbf{k}}|u^+_{\mathbf{k}'}\rangle\big|^2 = \frac{1}{2}\Big[1+\cos\theta\cos\theta'+\sin\theta\sin\theta'\cos(\phi'-\phi)\Big], \tag{186}$$

and the components of the partial derivative read

$$\frac{\partial}{\partial k_x}\arg(A) = \cos\phi\frac{\partial}{\partial k}\arg(A) - \frac{\sin\phi}{k}\frac{\partial}{\partial\phi}\arg(A), \tag{187}$$

$$\frac{\partial}{\partial k_y}\arg(A) = \sin\phi\frac{\partial}{\partial k}\arg(A) + \frac{\cos\phi}{k}\frac{\partial}{\partial\phi}\arg(A), \tag{188}$$

with $A \equiv \langle u^+_{\mathbf{k}}|u^+_{\mathbf{k}'}\rangle$. Note that for a general function $A(x)$, we have

$$\frac{\partial}{\partial x}\arg(A) = \frac{-i}{|A|^2}\Big(A^*\frac{\partial}{\partial x}A - \frac{1}{2}\frac{\partial}{\partial x}|A|^2\Big), \tag{189}$$

thus we obtain that

$$\frac{\partial}{\partial k}\arg(A) = \frac{1}{4|A|^2}\frac{\sin\theta\cos\theta}{k}\sin\theta'\sin(\phi'-\phi), \tag{190}$$

$$\frac{\partial}{\partial k'}\arg(A) = \frac{1}{4|A|^2}\frac{\sin\theta'\cos\theta'}{k'}\sin\theta\sin(\phi'-\phi), \tag{191}$$

$$\frac{\partial}{\partial \phi}\arg(A) = \frac{-1}{4|A|^2}\Big[\sin\theta\sin\theta'\cos(\phi'-\phi)+(1-\cos\theta)(1-\cos\theta')\Big], \tag{192}$$

$$\frac{\partial}{\partial \phi'}\arg(A) = \frac{1}{4|A|^2}\Big[\sin\theta\sin\theta'\cos(\phi'-\phi)+(1-\cos\theta)(1-\cos\theta')\Big], \tag{193}$$

and their combination gives

$$\frac{\partial}{\partial k_x}\arg(A)=\cos\phi\frac{\partial}{\partial k}\arg(A)-\frac{\sin\phi}{k}\frac{\partial}{\partial\phi}\arg(A)$$
$$=\frac{1}{4k|A|^2}\Big[\sin\theta\cos\theta\sin\theta'\sin(\phi'-\phi)\cos\phi+\sin\theta\sin\theta'\cos(\phi'-\phi)\sin\phi+(1-\cos\theta)(1-\cos\theta')\sin\phi\Big],\tag{194}$$

$$\frac{\partial}{\partial k'_x}\arg(A)=\cos\phi'\frac{\partial}{\partial k'}\arg(A)-\frac{\sin\phi'}{k'}\frac{\partial}{\partial\phi'}\arg(A)$$
$$=\frac{1}{4k'|A|^2}\Big[\sin\theta'\cos\theta'\sin\theta\sin(\phi'-\phi)\cos\phi'-\sin\theta\sin\theta'\cos(\phi'-\phi)\sin\phi'-(1-\cos\theta)(1-\cos\theta')\sin\phi'\Big],\tag{195}$$

$$\frac{\partial}{\partial k_y}\arg(A)=\sin\phi\frac{\partial}{\partial k}\arg(A)+\frac{\cos\phi}{k}\frac{\partial}{\partial\phi}\arg(A)$$
$$=\frac{1}{4k|A|^2}\Big[\sin\theta\cos\theta\sin\theta'\sin(\phi'-\phi)\sin\phi-\sin\theta\sin\theta'\cos(\phi'-\phi)\cos\phi-(1-\cos\theta)(1-\cos\theta')\cos\phi\Big],\tag{196}$$

$$\frac{\partial}{\partial k'_y}\arg(A)=\sin\phi'\frac{\partial}{\partial k'}\arg(A)+\frac{\cos\phi'}{k'}\frac{\partial}{\partial\phi'}\arg(A)$$
$$=\frac{1}{4k'|A|^2}\Big[\sin\theta'\cos\theta'\sin\theta\sin(\phi'-\phi)\sin\phi'+\sin\theta\sin\theta'\cos(\phi'-\phi)\cos\phi'+(1-\cos\theta)(1-\cos\theta')\cos\phi'\Big].\tag{197}$$

Then the contribution read

$$i\Big\langle u^+_{\mathbf{k}}\Big|\frac{\partial}{\partial k_x}\Big|u^+_{\mathbf{k}}\Big\rangle-\frac{\partial}{\partial k_x}\arg(A)=\frac{\sin\theta}{4k|A|^2}\Big(\sin\theta\cos\theta'\sin\phi-\cos\theta\sin\theta'\sin\phi'\Big),\tag{198}$$
$$-i\Big\langle u^+_{\mathbf{k}'}\Big|\frac{\partial}{\partial k'_x}\Big|u^+_{\mathbf{k}'}\Big\rangle-\frac{\partial}{\partial k'_x}\arg(A)=\frac{\sin\theta'}{4k'|A|^2}\Big(\sin\theta\cos\theta'\sin\phi-\cos\theta\sin\theta'\sin\phi'\Big),\tag{199}$$
$$i\Big\langle u^+_{\mathbf{k}}\Big|\frac{\partial}{\partial k_y}\Big|u^+_{\mathbf{k}}\Big\rangle-\frac{\partial}{\partial k_y}\arg(A)=\frac{\sin\theta}{4k|A|^2}\Big(\cos\theta\sin\theta'\cos\phi'-\sin\theta\cos\theta'\cos\phi\Big),\tag{200}$$
$$-i\Big\langle u^+_{\mathbf{k}'}\Big|\frac{\partial}{\partial k'_y}\Big|u^+_{\mathbf{k}'}\Big\rangle-\frac{\partial}{\partial k'_y}\arg(A)=\frac{\sin\theta'}{4k'|A|^2}\Big(\cos\theta\sin\theta'\cos\phi'-\sin\theta\cos\theta'\cos\phi\Big).\tag{201}$$

Consequently, the coordinate shift for a weak impurity potential $\hat{V}_{imp}$ can be written in components as

$$\delta r^x_{\mathbf{kk}'}=\frac{1}{4|A|^2}\Big(\frac{\sin\theta}{k}+\frac{\sin\theta'}{k'}\Big)\Big(\sin\theta\cos\theta'\sin\phi-\cos\theta\sin\theta'\sin\phi'\Big),\tag{202}$$
$$\delta r^y_{\mathbf{kk}'}=\frac{1}{4|A|^2}\Big(\frac{\sin\theta}{k}+\frac{\sin\theta'}{k'}\Big)\Big(\cos\theta\sin\theta'\cos\phi'-\sin\theta\cos\theta'\cos\phi\Big).\tag{203}$$

Thus we can formally write the coordinate shift for the upper band as

$$\delta\mathbf{r}_{\mathbf{kk}'}=\frac{1}{2}\Big(\frac{\varepsilon^0_{\mathbf{k}}}{\varepsilon^0_{\mathbf{k}'}}\Omega^+_{\mathbf{k}}+\frac{\varepsilon^0_{\mathbf{k}'}}{\varepsilon^0_{\mathbf{k}}}\Omega^+_{\mathbf{k}'}\Big)\cdot\frac{\hat{\mathbf{z}}\times(\mathbf{k}-\mathbf{k}')}{\big|\langle u^+_{\mathbf{k}}|u^+_{\mathbf{k}'}\rangle\big|^2}.\tag{204}$$

Anomalous Hall conductivity

In this section, we consider the anomalous Hall conductivity in the absence of time reversal symmetry to demonstrate that our theory can provide a identical description with previous works [3, 5]. For convenience and comparison, we

only consider the untilted Dirac fermion ($t = 0$). The system is then isotropic with the constant relaxation time

$$\begin{aligned}\frac{1}{\tau} &= \int \frac{d^2k'}{(2\pi)^2} \varpi^{(2)}_{\mathbf{k}\mathbf{k}'}[1-\cos(\phi-\phi')]\delta(\varepsilon_{\mathrm{F}}-\varepsilon^0_{\mathbf{k}'}) \\ &= \frac{n_i V_0^2}{4\pi\hbar}\int_0^\infty k'dk' \int_0^{2\pi} d\phi' \left[1+\cos^2\theta+\sin^2\theta\cos(\phi-\phi')\right]\left[1-\cos(\phi-\phi')\right]\delta(\varepsilon^0_{\mathbf{k}}-\varepsilon^0_{\mathbf{k}'})\delta(\varepsilon_{\mathrm{F}}-\varepsilon^0_{\mathbf{k}'}) \\ &= \frac{n_i V_0^2}{4\hbar}\frac{\varepsilon_{\mathrm{F}}^2+3m^2}{v^2\varepsilon_{\mathrm{F}}},\end{aligned} \tag{205}$$

and the anomalous Hall conductivity in x-y plane reads

$$\sigma^A_{xy} = \sigma^{in}_{xy} + \sigma^{sj}_{xy} + \sigma^{sk}_{xy} \tag{206}$$

with

$$\sigma^{in}_{xy} = -\frac{e^2}{\hbar}\sum_l \varepsilon^{xyz}\Omega^z_l f^{(0)}_l, \tag{207}$$

$$\sigma^{sj,1}_{xy} = -\frac{e^2\tau}{\hbar}\int [dk] v^{sj}_x \partial^y_{\mathbf{k}} f^{(0)}_{\mathbf{k}}, \tag{208}$$

$$\sigma^{sj.2}_{xy} = \frac{e^2\tau}{\hbar}\int [dk] v^{sj}_y \partial^x_{\mathbf{k}} f^{(0)}_{\mathbf{k}}, \tag{209}$$

$$\sigma^{sk}_{xy} = -\frac{e^2\tau^2}{\hbar}\int [dk]\int [dk'] \varpi^{as}_{\mathbf{k}\mathbf{k}'}(v^x_{\mathbf{k}} - v^x_{\mathbf{k}'})\partial^y_{\mathbf{k}} f^{(0)}_{\mathbf{k}}, \tag{210}$$

To obtain the expression of σ^{sk}_{xy}, we used that $\varpi^{as}_{ll'} = -\varpi^{as}_{l'l}$. The l index left in σ^{in}_{xy} indicate that the intrinsic contribution need the summation over all the filled bands. We assume that the Fermi level is in the upper band, thus the band index can be neglected in the side-jump and skew-scattering contributions.

Intrinsic contribution

Since the intrinsic contribution comes from the whole Fermi sea, both the totally filled negative energy and the partially filled positive energy bands contribute to it.

$$\begin{aligned}\sigma^{in}_{xy} &= -\frac{e^2}{\hbar}\sum_l \varepsilon^{xyz}\Omega^z_l f^{(0)}_l \\ &= -\frac{e^2}{\hbar}\Big(\int [dk]\Omega^-_{\mathbf{k}} + \int_{\varepsilon^+_{\mathbf{k}}<\varepsilon_{\mathrm{F}}} [dk]\Omega^+_{\mathbf{k}}\Big) \\ &= -\frac{e^2 m}{4\pi\hbar\sqrt{v^2k_{\mathrm{F}}^2+m^2}},\end{aligned} \tag{211}$$

which is consistent with the result Eq. (58) in [5].

Side-jump contribution

For $t < v$, the direct interband scattering is not energetically allowed in the weak disorder limit of our model. We consider only the coordinate shift effect of scattering in the upper band, which can be simplified in the $t = 0$ case as

$$\delta\mathbf{r}_{\mathbf{k}\mathbf{k}'} = \frac{\Omega^+_{\mathbf{k}}\cdot\hat{\mathbf{z}}\times(\mathbf{k}-\mathbf{k}')}{|\langle u^+_{\mathbf{k}}|u^+_{\mathbf{k}'}\rangle|^2}, \tag{212}$$

and the corresponding side-jump velocity read

$$\mathbf{v}^{sj}_{\mathbf{k}} = \int [dk']\varpi^{(2)}_{\mathbf{k}'\mathbf{k}}\delta\mathbf{r}_{\mathbf{k}'\mathbf{k}} = \frac{n_i V_0^2 m}{2\hbar(\varepsilon^0_{\mathbf{k}})^2}\hat{\mathbf{z}}\times\mathbf{k}, \tag{213}$$

by noting that

$$\varpi^{(2)}_{\mathbf{k}\mathbf{k}'}(t=0) = \frac{\pi n_i V_0^2}{\hbar}\big[1+\cos^2\theta + \sin^2\theta\cos(\phi'-\phi)\big]\delta(\varepsilon^0_{\mathbf{k}} - \varepsilon^0_{\mathbf{k}'}). \tag{214}$$

For comparison, we divide the side-jump contribution into two parts as

$$\sigma^{sj}_{xy} = \sigma^{sj,1}_{xy} + \sigma^{sj,2}_{xy} \tag{215}$$

with

$$\begin{aligned}
\sigma^{sj,1}_{xy} &= e^2\tau\int [dk] v^x_{sj} v^y_{\mathbf{k}}\delta(\varepsilon_{\mathrm{F}} - \varepsilon^0_{\mathbf{k}}) \\
&= -\frac{n_i V_0^2 e^2 m\tau}{8\pi^2\hbar^2}\int_0^\infty kdk\int_0^{2\pi} d\phi \frac{k\sin\phi}{(\varepsilon^0_{\mathbf{k}})^2}\frac{v^2 k\sin\phi}{\varepsilon^0_{\mathbf{k}}}\delta(\varepsilon_{\mathrm{F}} - \varepsilon^0_{\mathbf{k}}) \\
&= -\frac{n_i V_0^2 e^2 m\tau}{8\pi\hbar^2}\int_0^\infty dk \frac{v^2k^3}{(\varepsilon^0_{\mathbf{k}})^3}\frac{\partial k}{\partial \varepsilon^0_{\mathbf{k}}}\delta(k_{\mathrm{F}} - k) \\
&= -\frac{n_i V_0^2 e^2 m}{8\pi\hbar^2}\Big(\frac{4\hbar}{n_i V_0^2}\frac{v^2\varepsilon_{\mathrm{F}}}{\varepsilon_{\mathrm{F}}^2 + 3m^2}\Big)\frac{k_{\mathrm{F}}^2}{\varepsilon_{\mathrm{F}}^2} \\
&= -\frac{e^2 m v^2 k_{\mathrm{F}}^2}{2\pi\hbar\varepsilon_{\mathrm{F}}(\varepsilon_{\mathrm{F}}^2 + 3m^2)},
\end{aligned} \tag{216}$$

and

$$\begin{aligned}
\sigma^{sj,2}_{xy} &= -e^2\tau\int [dk] v^y_{sj} v^x_{\mathbf{k}}\delta(\varepsilon_{\mathrm{F}} - \varepsilon^0_{\mathbf{k}}) \\
&= -\frac{n_i V_0^2 e^2 m\tau}{8\pi^2\hbar^2}\int_0^\infty kdk\int_0^{2\pi} d\phi \frac{k\cos\phi}{(\varepsilon^0_{\mathbf{k}})^2}\frac{v^2 k\cos\phi}{\varepsilon^0_{\mathbf{k}}}\delta(\varepsilon_{\mathrm{F}} - \varepsilon^0_{\mathbf{k}}) \\
&= -\frac{n_i V_0^2 e^2 m\tau}{8\pi\hbar^2}\int_0^\infty dk \frac{v^2k^3}{(\varepsilon^0_{\mathbf{k}})^3}\frac{\partial k}{\partial \varepsilon^0_{\mathbf{k}}}\delta(k_{\mathrm{F}} - k) \\
&= -\frac{n_i V_0^2 e^2 m}{8\pi\hbar^2}\Big(\frac{4\hbar}{n_i V_0^2}\frac{v^2\varepsilon_{\mathrm{F}}}{\varepsilon_{\mathrm{F}}^2 + 3m^2}\Big)\frac{k_{\mathrm{F}}^2}{\varepsilon_{\mathrm{F}}^2} \\
&= -\frac{e^2 m v^2 k_{\mathrm{F}}^2}{2\pi\hbar\varepsilon_{\mathrm{F}}(\varepsilon_{\mathrm{F}}^2 + 3m^2)},
\end{aligned} \tag{217}$$

which are consistent with the results Eq. (65) and (67) in [5], respectively. Consequently, the total side-jump scattering contribution reads

$$\sigma^{sj}_{xy} = -\frac{e^2 m v^2 k_{\mathrm{F}}^2}{\pi\hbar\varepsilon_{\mathrm{F}}(\varepsilon_{\mathrm{F}}^2 + 3m^2)}, \tag{218}$$

which is consistent with the result Eq. (68) in [5].

Skew-scattering contribution

The third and fourth-order antisymmetric scattering rates can be simplified in the $t = 0$ case as

$$\varpi^{(3a)}_{\mathbf{k}\mathbf{k}'}(t=0) = \frac{\pi n_i V_1^3}{2\hbar}\frac{mk^2}{(\varepsilon^0_{\mathbf{k}})^2}\sin(\phi-\phi')\delta(\varepsilon^0_{\mathbf{k}} - \varepsilon^0_{\mathbf{k}'}) \tag{219}$$

$$\varpi^{(4a)}_{\mathbf{k}\mathbf{k}'}(t=0) = \frac{3\pi n_i^2 V_0^4}{4\hbar}\frac{mk^2}{(\varepsilon^0_{\mathbf{k}})^3}\sin(\phi-\phi')\delta(\varepsilon^0_{\mathbf{k}} - \varepsilon^0_{\mathbf{k}'}), \tag{220}$$

thus the skew-scattering contribution can be divided into two parts as

$$\sigma^{sk}_{xy} = \sigma^{sk,1}_{xy} + \sigma^{sk,2}_{xy} \tag{221}$$

with

$$\begin{aligned}
\sigma_{xy}^{sk,1} &= e^2\tau^2 \int [dk] \int [dk'] v_{\mathbf{k}}^{y} \varpi_{\mathbf{k}\mathbf{k}'}^{4a} (v_{\mathbf{k}}^{x} - v_{\mathbf{k}'}^{x}) \delta(\varepsilon_{\mathrm{F}} - \varepsilon_{\mathbf{k}}^{0}) \\
&= \frac{3n_i^2 V_0^4 e^2\tau^2}{64\pi^3\hbar} \int_0^\infty dk \int_0^{2\pi} d\phi \int_0^\infty dk' \int_0^{2\pi} d\phi' \frac{v^4 k^2 k'}{(\varepsilon_{\mathbf{k}}^{0})^2} \frac{mk^2}{(\varepsilon_{\mathbf{k}}^{0})^3} \sin\phi \sin(\phi-\phi')(k\cos\phi \\
&\quad -k'\cos\phi') \delta(\varepsilon_{\mathbf{k}}^{0} - \varepsilon_{\mathbf{k}'}^{0}) \delta(\varepsilon_{\mathrm{F}} - \varepsilon_{\mathbf{k}}^{0}) \\
&= -\frac{3n_i^2 V_0^4 e^2\tau^2}{64\pi\hbar} \int_0^\infty dk \frac{v^4 k_\perp^4}{(\varepsilon_{\mathbf{k}}^{0})^2} \frac{mk^2}{(\varepsilon_{\mathbf{k}}^{0})^3} \Big(\frac{\partial k}{\partial \varepsilon_{\mathbf{k}}^{0}}\Big)^2 \delta(k_{\mathrm{F}} - k) \\
&= -\frac{3n_i^2 V_0^4 e^2 m}{64\pi\hbar} \Big(\frac{4\hbar}{n_i V_0^2} \frac{v^2 \varepsilon_{\mathrm{F}}}{\varepsilon_{\mathrm{F}}^2 + 3m^2}\Big)^2 \frac{k_{\mathrm{F}}^4}{\varepsilon_{\mathrm{F}}^3} \\
&= -\frac{3e^2 m v^4 k_{\mathrm{F}}^4}{4\pi\hbar\varepsilon_{\mathrm{F}}(\varepsilon_{\mathrm{F}}^2 + 3m^2)^2}.
\end{aligned} \tag{222}$$

and

$$\begin{aligned}
\sigma_{xy}^{sk,2} &= e^2\tau^2 \int [dk] \int [dk'] v_{\mathbf{k}}^{y} \varpi_{\mathbf{k}\mathbf{k}'}^{3a} (v_{\mathbf{k}}^{x} - v_{\mathbf{k}'}^{x}) \delta(\varepsilon_{\mathrm{F}} - \varepsilon_{\mathbf{k}}^{0}) \\
&= \frac{n_i V_1^3 e^2\tau^2}{32\pi^3\hbar} \int_0^\infty dk \int_0^{2\pi} d\phi \int_0^\infty dk' \int_0^{2\pi} d\phi' \frac{v^4 k^2 k'}{(\varepsilon_{\mathbf{k}}^{0})^2} \frac{mk^2}{(\varepsilon_{\mathbf{k}}^{0})^2} \sin\phi \sin(\phi-\phi')(k\cos\phi \\
&\quad -k'\cos\phi') \delta(\varepsilon_{\mathbf{k}}^{0} - \varepsilon_{\mathbf{k}'}^{0}) \delta(\varepsilon_{\mathrm{F}} - \varepsilon_{\mathbf{k}}^{0}) \\
&= -\frac{n_i V_1^3 e^2\tau^2}{32\pi\hbar} \int_0^\infty dk \frac{v^4 k_\perp^4}{(\varepsilon_{\mathbf{k}}^{0})^2} \frac{mk^2}{(\varepsilon_{\mathbf{k}}^{0})^2} \Big(\frac{\partial k}{\partial \varepsilon_{\mathbf{k}}^{0}}\Big)^2 \delta(k_{\mathrm{F}} - k) \\
&= -\frac{n_i V_1^3 e^2 m}{32\pi\hbar} \Big(\frac{4\hbar}{n_i V_0^2} \frac{v^2 \varepsilon_{\mathrm{F}}}{\varepsilon_{\mathrm{F}}^2 + 3m^2}\Big)^2 \frac{k_{\mathrm{F}}^4}{\varepsilon_{\mathrm{F}}^2} \\
&= -\frac{e^2 V_1^3}{2\pi\hbar n_i V_0^4} \frac{m v^4 k_{\mathrm{F}}^4}{(\varepsilon_{\mathrm{F}}^2 + 3m^2)^2},
\end{aligned} \tag{223}$$

Consequently, the total skew-scattering contribution reads

$$\sigma_{xy}^{sk} = -\frac{3e^2 m v^4 k_{\mathrm{F}}^4}{4\pi\hbar\varepsilon_{\mathrm{F}}(\varepsilon_{\mathrm{F}}^2 + 3m^2)^2} - \frac{e^2 V_1^3}{2\pi\hbar n_i V_0^4} \frac{m v^4 k_{\mathrm{F}}^4}{(\varepsilon_{\mathrm{F}}^2 + 3m^2)^2}, \tag{224}$$

which is consistent with the result Eq. (72) in [5]. In summary, the anomalous Hall conductivity obtained within our scheme at $t = 0$ is consistent with precious results [5].

Nonlinear Hall conductivity

For $t < v$, the direct interband scattering is not energetically allowed in the weak disorder limit of our model, thus we can drop the band index and rewritten the nonlinear Hall conductivity with double-frequency. To obtain the analytical results, we consider the $t \ll v$ limit, in which we can neglected the angular dependence of τ_l [6, 7]. For simplicity, we adopt the constant relaxation time Eq. (205) as an approximation in the $t \ll v$ limit and expand the results up to the first order in t, the nonlinear Hall conductivity can be simplified as

$$\chi_{abc}^{in} = -\frac{e^3\tau}{2\hbar^2} \int [dk] \varepsilon^{acd} \Omega_{\mathbf{k}}^{d} \partial_{\mathbf{k}}^{b} f_{\mathbf{k}}^{(0)}, \tag{225}$$

$$\chi_{abc}^{sj,1} = \frac{e^3\tau^2}{2\hbar^2} \int [dk] (\partial_{\mathbf{k}}^{c} v_a^{sj}) \partial_{\mathbf{k}}^{b} f_{\mathbf{k}}^{(0)}, \tag{226}$$

$$\chi_{abc}^{sj,2} = -\frac{e^3\tau^2}{2\hbar} \int [dk] \Big[(\partial_{\mathbf{k}}^{a} v_c^{sj} + \mathcal{M}_{\mathbf{k}}^{ac}) v_{\mathbf{k}}^{b} + (\partial_{\mathbf{k}}^{c} v_{\mathbf{k}}^{a}) v_b^{sj} \Big] \frac{\partial f_{\mathbf{k}}^{(0)}}{\partial \varepsilon_{\mathbf{k}}}, \tag{227}$$

$$\chi_{abc}^{sk} = \frac{e^3\tau^3}{2\hbar^2} \int [dk] \int [dk'] \varpi_{\mathbf{k}\mathbf{k}'}^{as} \Big[(\partial_{\mathbf{k}}^{c} v_{\mathbf{k}}^{a} - \partial_{\mathbf{k}'}^{c} v_{\mathbf{k}'}^{a}) \partial_{\mathbf{k}}^{b} f_{\mathbf{k}}^{(0)} - (v_{\mathbf{k}}^{a} - v_{\mathbf{k}'}^{a}) \partial_{\mathbf{k}}^{c} \partial_{\mathbf{k}}^{b} f_{\mathbf{k}}^{(0)} \Big], \tag{228}$$

where we only considered χ_{abc} because $\chi_{abc} = \xi_{abc}$ in the low frequency limit. And the explicit expression of $\mathcal{M}_{\mathbf{k}}^{ab}$ for the tilted 2D Dirac model can be found in Supplementary Methods. .

Intrinsic contribution

Here we denote the contribution related to the Berry dipole as the intrinsic contribution, although it actually depend on the relaxation time. Within a **k**-independent relaxation time approximation, the intrinsic contribution read

$$\begin{aligned}\chi_{yxx}^{in} &= \frac{e^3\tau}{2\hbar^2}\int\frac{d^2k}{(2\pi)^2}\Omega_{\mathbf{k}}^{+}\frac{\partial\varepsilon_{\mathbf{k}}^{+}}{\partial k_x}\frac{\partial f_{\mathbf{k}}^{(0)}}{\partial\varepsilon_{\mathbf{k}}^{+}}\\ &\simeq\frac{e^3}{h}\frac{tm}{n_iV_0^2}\frac{3v^2(\varepsilon_{\mathrm{F}}^2-m^2)}{2\varepsilon_{\mathrm{F}}^3(\varepsilon_{\mathrm{F}}^2+3m^2)}.\end{aligned}\tag{229}$$

For the other component, we have

$$\begin{aligned}\chi_{xyy}^{in} &= \frac{e^3\tau}{2\hbar^2}\int\frac{d^2k}{(2\pi)^2}\Omega_{\mathbf{k}}^{+}\frac{\partial\varepsilon_{\mathbf{k}}^{+}}{\partial k_y}\frac{\partial f_{\mathbf{k}}^{(0)}}{\partial\varepsilon_{\mathbf{k}}^{+}}\\ &=0,\end{aligned}\tag{230}$$

which is consistent with the symmetry arguments [8].

Side-jump contribution

Armed with the side-jump related quantities that we have obtained (see Supplementary Methods.), we consider the $t \ll v$ expansion for the side-jump contribution to the nonlinear Hall conductivity. The first part of the side-jump contribution is induced the side-jump velocity, which reads

$$\begin{aligned}\chi_{yxx}^{sj,1} &= \frac{e^3\tau^2}{2\hbar^2}\int\frac{d^2k}{(2\pi)^2}(\partial_{\mathbf{k}}^{x}v_y^{sj})\frac{\partial\varepsilon_{\mathbf{k}}^{+}}{\partial k_x}\frac{\partial f_{\mathbf{k}}^{(0)}}{\partial\varepsilon_{\mathbf{k}}^{+}}\\ &\simeq\frac{e^3}{h}\frac{tm}{n_iV_0^2}\frac{v^2(\varepsilon_{\mathrm{F}}^2-m^2)(5\varepsilon_{\mathrm{F}}^2-33m^2)}{4\varepsilon_{\mathrm{F}}^3(\varepsilon_{\mathrm{F}}^2+3m^2)^2}.\end{aligned}\tag{231}$$

For the other component, we have

$$\begin{aligned}\chi_{xyy}^{sj,1} &= \frac{e^3\tau^2}{2\hbar^2}\int\frac{d^2k}{(2\pi)^2}(\partial_{\mathbf{k}}^{y}v_x^{sj})\frac{\partial\varepsilon_{\mathbf{k}}^{+}}{\partial k_y}\frac{\partial f_{\mathbf{k}}^{(0)}}{\partial\varepsilon_{\mathbf{k}}^{+}}\\ &=0.\end{aligned}\tag{232}$$

The second part of the side-jump contribution comes from the coordinate shift induced modification to the distribution function, which can be obtained as

$$\begin{aligned}\chi_{yxx}^{sj,2} &= -\frac{e^3\tau^2}{2\hbar}\int\frac{d^2k}{(2\pi)^2}\left[(\partial_{\mathbf{k}}^{y}v_x^{sj}+\mathcal{M}_{\mathbf{k}}^{yx})v_{\mathbf{k}}^{x}+(\partial_{\mathbf{k}}^{x}v_{\mathbf{k}}^{y})v_x^{sj}\right]\frac{\partial f_{\mathbf{k}}^{(0)}}{\partial\varepsilon_{\mathbf{k}}^{+}}\\ &\simeq-\frac{e^3}{h}\frac{tm}{n_iV_0^2}\frac{v^2(\varepsilon_{\mathrm{F}}^2-m^2)(3\varepsilon_{\mathrm{F}}^2+17m^2)}{4\varepsilon_{\mathrm{F}}^3(\varepsilon_{\mathrm{F}}^2+3m^2)^2}.\end{aligned}\tag{233}$$

For the other component, we have

$$\begin{aligned}\chi_{xyy}^{sj,2} &= -\frac{e^3\tau^2}{2\hbar}\int\frac{d^2k}{(2\pi)^2}\left[(\partial_{\mathbf{k}}^{x}v_y^{sj}+\mathcal{M}_{\mathbf{k}}^{xy})v_{\mathbf{k}}^{y}+(\partial_{\mathbf{k}}^{y}v_{\mathbf{k}}^{x})v_y^{sj}\right]\frac{\partial f_{\mathbf{k}}^{(0)}}{\partial\varepsilon_{\mathbf{k}}^{+}}\\ &=0.\end{aligned}\tag{234}$$

Consequently, the total side-jump contribution reads

$$\chi_{yxx}^{sj}=\frac{e^3}{h}\frac{tm}{n_iV_0^2}\frac{v^2(\varepsilon_{\mathrm{F}}^2-m^2)(\varepsilon_{\mathrm{F}}^2-25m^2)}{2\varepsilon_{\mathrm{F}}^3(\varepsilon_{\mathrm{F}}^2+3m^2)^2},\tag{235}$$

$$\chi_{xyy}^{sj}=0.\tag{236}$$

Thus, the side-jump contribution to the nonlinear Hall conductivity is also consistent with the previous symmetry arguments [8].

Skew-scattering contribution

Armed with the skew-scattering related quantities that we have obtained (see Supplementary Methods.), we consider the $t \ll v$ expansion for the skew-scattering contribution to the nonlinear Hall conductivity. For contribution from the antisymmetric fourth-order scattering, we have

$$\begin{aligned}\chi^{sk,1}_{yxx} &= \frac{e^3\tau^3}{2\hbar^2}\int\frac{d^2k}{(2\pi)^2}\int\frac{d^2k'}{(2\pi)^2}\Big\{\varpi^{(4a)}_{\mathbf{k}\mathbf{k}'}(\partial^x_{\mathbf{k}}v^y_{\mathbf{k}} - \partial^x_{\mathbf{k}'}v^y_{\mathbf{k}'}) + \partial^x_{\mathbf{k}}\big[\varpi^{(4a)}_{\mathbf{k}\mathbf{k}'}(v^y_{\mathbf{k}} - v^y_{\mathbf{k}'})\big]\Big\}\frac{\partial\varepsilon^+_{\mathbf{k}}}{\partial k_x}\frac{\partial f^{(0)}_{\mathbf{k}}}{\partial\varepsilon^+_{\mathbf{k}}} \\ &\simeq -\frac{e^3}{h}\frac{tm}{n_iV_0^2}\frac{v^2(\varepsilon_{\mathrm{F}}^2 - m^2)^2(13\varepsilon_{\mathrm{F}}^2 + 77m^2)}{4\varepsilon_{\mathrm{F}}^3(\varepsilon_{\mathrm{F}}^2+3m^2)^3},\end{aligned} \tag{237}$$

and the other component is

$$\begin{aligned}\chi^{sk,1}_{xyy} &= \frac{e^3\tau^3}{2\hbar^2}\int\frac{d^2k}{(2\pi)^2}\int\frac{d^2k'}{(2\pi)^2}\Big\{\varpi^{(4a)}_{\mathbf{k}\mathbf{k}'}(\partial^y_{\mathbf{k}}v^x_{\mathbf{k}} - \partial^y_{\mathbf{k}'}v^x_{\mathbf{k}'}) + \partial^y_{\mathbf{k}}\big[\varpi^{(4a)}_{\mathbf{k}\mathbf{k}'}(v^x_{\mathbf{k}} - v^x_{\mathbf{k}'})\big]\Big\}\frac{\partial\varepsilon^+_{\mathbf{k}}}{\partial k_y}\frac{\partial f^{(0)}_{\mathbf{k}}}{\partial\varepsilon^+_{\mathbf{k}}} \\ &= 0.\end{aligned} \tag{238}$$

For contribution from the antisymmetric third-order scattering, we have

$$\begin{aligned}\chi^{sk,2}_{yxx} &= \frac{e^3\tau^3}{2\hbar^2}\int\frac{d^2k}{(2\pi)^2}\int\frac{d^2k'}{(2\pi)^2}\Big\{\varpi^{(3a)}_{\mathbf{k}\mathbf{k}'}(\partial^x_{\mathbf{k}}v^y_{\mathbf{k}} - \partial^x_{\mathbf{k}'}v^y_{\mathbf{k}'}) + \partial^x_{\mathbf{k}}\big[\varpi^{(3a)}_{\mathbf{k}\mathbf{k}'}(v^y_{\mathbf{k}} - v^y_{\mathbf{k}'})\big]\Big\}\frac{\partial\varepsilon^+_{\mathbf{k}}}{\partial k_x}\frac{\partial f^{(0)}_{\mathbf{k}}}{\partial\varepsilon^+_{\mathbf{k}}} \\ &\simeq -\frac{e^3}{h}\frac{tm}{n_i^2V_0^6/V_1^3}\frac{v^2(\varepsilon_{\mathrm{F}}^2 - m^2)^2(5\varepsilon_{\mathrm{F}}^2 + 9m^2)}{\varepsilon_{\mathrm{F}}^2(\varepsilon_{\mathrm{F}}^2+3m^2)^3},\end{aligned} \tag{239}$$

and the other component is

$$\begin{aligned}\chi^{sk,2}_{xyy} &= \frac{e^3\tau^3}{2\hbar^2}\int\frac{d^2k}{(2\pi)^2}\int\frac{d^2k'}{(2\pi)^2}\Big\{\varpi^{(3a)}_{\mathbf{k}\mathbf{k}'}(\partial^y_{\mathbf{k}}v^x_{\mathbf{k}} - \partial^y_{\mathbf{k}'}v^x_{\mathbf{k}'}) + \partial^y_{\mathbf{k}}\big[\varpi^{(3a)}_{\mathbf{k}\mathbf{k}'}(v^x_{\mathbf{k}} - v^x_{\mathbf{k}'})\big]\Big\}\frac{\partial\varepsilon^+_{\mathbf{k}}}{\partial k_y}\frac{\partial f^{(0)}_{\mathbf{k}}}{\partial\varepsilon^+_{\mathbf{k}}} \\ &= 0.\end{aligned} \tag{240}$$

Consequently, the total skew-scattering contribution reads

$$\chi^{sk}_{yxx} = -\frac{e^3}{h}\frac{tm}{n_iV_0^2}\frac{v^2(\varepsilon_{\mathrm{F}}^2 - m^2)^2(13\varepsilon_{\mathrm{F}}^2 + 77m^2)}{4\varepsilon_{\mathrm{F}}^3(\varepsilon_{\mathrm{F}}^2+3m^2)^3} - \frac{e^3}{h}\frac{tm}{n_i^2V_0^6/V_1^3}\frac{v^2(\varepsilon_{\mathrm{F}}^2 - m^2)^2(5\varepsilon_{\mathrm{F}}^2 + 9m^2)}{\varepsilon_{\mathrm{F}}^2(\varepsilon_{\mathrm{F}}^2+3m^2)^3}, \tag{241}$$

$$\chi^{sk}_{xyy} = 0 \tag{242}$$

Thus, the skew-scattering contribution to the nonlinear Hall conductivity is also consistent with the previous symmetry arguments [8].

SUPPLEMENTARY NOTE 5

Scaling law: The scaling law of the anomalous and nonlinear Hall effects describe the scaling behaviour of the Hall resistivity as a function of the longitudinal resistivity.

Scaling law of anomalous Hall effect

Within the constant relaxation time approximation, the anomalous Hall conductivity can be obtained as

$$\sigma^A_{xy} = \sigma^{in}_{xy} + \sigma^{sj}_{xy} + \sigma^{sk,1}_{xy} + \sigma^{sk,2}_{xy}, \tag{243}$$

$$\sigma^{in}_{xy} = -\frac{e^2m}{4\pi\hbar\sqrt{v^2k_{\mathrm{F}}^2+m^2}} \sim (n_iV_0^2)^0, \tag{244}$$

$$\sigma^{sj}_{xy} = -\frac{e^2mv^2k_{\mathrm{F}}^2}{\pi\hbar\varepsilon_{\mathrm{F}}(\varepsilon_{\mathrm{F}}^2+3m^2)} \sim (n_iV_0^2)^0, \tag{245}$$

$$\sigma^{sk,1}_{xy} = -\frac{3e^2mv^4k_{\mathrm{F}}^4}{4\pi\hbar\varepsilon_{\mathrm{F}}(\varepsilon_{\mathrm{F}}^2+3m^2)^2} \sim (n_iV_0^2)^0, \tag{246}$$

$$\sigma^{sk,2}_{xy} = -\frac{e^2V_1^3}{2\pi\hbar n_iV_0^4}\frac{mv^4k_{\mathrm{F}}^4}{(\varepsilon_{\mathrm{F}}^2+3m^2)^2} \sim \frac{n_iV_1^3}{(n_iV_0^2)^2}, \tag{247}$$

which indicate that the intrinsic, side-jump and intrinsic skew-scattering contributions are irrelevant with the disorder concentration n_i and scattering strength V_0. It is worth noting that these results are obtained based on a weak scattering expansion where only one scattering mechanism dominates. When multiple sources of scattering are present, $\tau_{\mathbf{k}}$ would correspond to the total transport scattering time. While the other scattering contributions, such as those to the side-jump and skew-scattering, would not necessarily have the same form, which invalidate the results with cancellation of $n_i V_0^2$. Thus, in general, it is more appropriate to write that

$$\sigma_{xy}^{in} \sim const, \qquad \sigma_{xy}^{sj} \sim \frac{\mathcal{S}_{sj}}{\mathcal{S}_{tot}}, \qquad \sigma_{xy}^{sk,1} \sim \frac{\mathcal{S}_{sk,g}^2}{\mathcal{S}_{tot}^2}, \qquad \sigma_{xy}^{sk,2} \sim \frac{\mathcal{S}_{sk,ng}}{\mathcal{S}_{tot}^2} \tag{248}$$

with

$$\mathcal{S}_{tot} = \sum_i \mathcal{S}_i, \qquad \mathcal{S}_{sj} = \sum_{i \in sj} \mathcal{S}_i, \qquad \mathcal{S}_{sk,g} = \sum_{i \in sk,g} \mathcal{S}_i, \qquad \mathcal{S}_{sk,ng} = \sum_{i \in sk,ng} \mathcal{S}_i. \tag{249}$$

Here $\mathcal{S}_i$ is the scattering contribution from mechanism i and $i \in sj$, $i \in sk, g$ and $i \in sk, ng$ represent the summation within the subgroup of the total mechanisms that contribute to the side-jump, intrinsic- and extrinsic-skew-scattering. For simplicity, we assume that no correlation between different scattering sources, thus each scattering contributes to total resistivity independently as dictated by Matthiessens rule

$$\mathcal{S}_{tot} = \sum_i \mathcal{S}_i \qquad \Leftrightarrow \qquad \rho_{xx} = \sum_i \rho_i. \tag{250}$$

To obtain the scaling law, we need to transform the scattering dependence into the resistivity dependence, thus we can estimate $\mathcal{S}_{tot}$ with ρ_{xx} and $\mathcal{S}_i$ with ρ_i. Noting that

1. All the scattering mechanism can have side-jump and intrinsic skew-scattering contributions while only the static scattering can induce the extrinsic skew-scattering (non-Gaussian) contribution.

2. The summation of the scattering contributions to side-jump and skew-scattering conductivity have certain form factor related to the coordinate shift and antisymmetric scattering rate.

Then we can write that

$$-\sigma_{xy}^A = \mathcal{C}^{in} + \sum_i \mathcal{C}_i^{sj} \frac{\rho_i}{\rho_{xx}} + \sum_{ij} \mathcal{C}_{ij}^{sk,1} \frac{\rho_i \rho_j}{\rho_{xx}^2} + \sum_{i \in S} \mathcal{C}_i^{sk,2} \frac{\rho_i}{\rho_{xx}^2} \tag{251}$$

with $\mathcal{C}^{in}$, $\mathcal{C}_i^{sj}$, $\mathcal{C}_{ij}^{sk,1}$ and $\mathcal{C}_i^{sk,2}$ are the coefficients that related but not equal to the intrinsic, side-jump, intrinsic and extrinsic skew-scattering contributions. And $i \in S$ indicates that only static disorder scatterings are included in the summation. This expression reproduce the result Eq. (2) in [9]. Note that $\rho_{xy}^A \simeq -\sigma_{xy}^A \rho_{xx}^2$, thus we obtain that

$$\rho_{xy}^A = \mathcal{C}^{in} \rho_{xx}^2 + \sum_i \mathcal{C}_i^{sj} \rho_i \rho_{xx} + \sum_{ij} \mathcal{C}_{ij}^{sk,1} \rho_i \rho_j + \sum_{i \in S} \mathcal{C}_i^{sk,2} \rho_i, \tag{252}$$

which is the general scaling law Eq. (1) proposed in [9].

Scaling law of nonlinear Hall effect

According to the calculation of the nonlinear Hall conductivity contributions, we have

$$\chi_{yxx}^{in} \sim \frac{1}{\mathcal{S}_{tot}}, \qquad \chi_{yxx}^{sj} \sim \frac{\mathcal{S}_{sj}}{\mathcal{S}_{tot}^2}, \qquad \chi_{yxx}^{sk,1} \sim \frac{\mathcal{S}_{sk,g}^2}{\mathcal{S}_{tot}^3}, \qquad \chi_{yxx}^{sk,2} \sim \frac{\mathcal{S}_{sk,ng}}{\mathcal{S}_{tot}^3}. \tag{253}$$

Assume a experimental setup with the driving electric current along x direction and the nonlinear Hall voltage is measured along y, we have that $V_y^{2\omega}/(V_x^\omega)^2 = \chi_{yxx}\rho_{xx}$, where $V_y^{2\omega}$ and V_x^ω refer to the nonlinear Hall and linear longitudinal voltage, respectively. Thus the scaling law of the nonlinear Hall effect is

$$\frac{V_y^{2\omega}}{(V_x^\omega)^2} = \mathcal{C}^{in} + \sum_i \mathcal{C}_i^{sj} \frac{\rho_i}{\rho_{xx}} + \sum_{ij} \mathcal{C}_{ij}^{sk,1} \frac{\rho_i \rho_j}{\rho_{xx}^2} + \sum_{i \in S} \mathcal{C}_i^{sk,2} \frac{\rho_i}{\rho_{xx}^2}. \tag{254}$$

Here $\mathcal{C}^{in}$, $\mathcal{C}_i^{sj}$, $\mathcal{C}_{ij}^{sk,1}$ and $\mathcal{C}_i^{sk,2}$ are the coefficients that related but not equal to the intrinsic, side-jump, intrinsic and extrinsic skew-scattering contributions. It is interesting to note that the nonlinear Hall response $V_y^{2\omega}/(V_x^\omega)^2$ has a

similar scaling behaviour with the anomalous Hall conductivity σ_{xy}^A. Then it is natural to expecting that a similar experimental setups with that in the anomalous Hall effect [9, 10] can help to separate these contributions.

Following [9], we specialize Eq. (254) to two major competing scattering sources: one static and one dynamic, thus we have

$$\frac{V_y^{2\omega}}{(V_x^{\omega})^2} = \mathcal{C}^{in} + \mathcal{C}_0^{sj}\frac{\rho_{xx0}}{\rho_{xx}} + \mathcal{C}_1^{sj}\frac{\rho_{xxT}}{\rho_{xx}} + \mathcal{C}_{00}^{sk,1}\frac{\rho_{xx0}\rho_{xx0}}{\rho_{xx}^2} + \mathcal{C}_{01}^{sk,1}\frac{\rho_{xx0}\rho_{xxT}}{\rho_{xx}^2} + \mathcal{C}_{11}^{sk,1}\frac{\rho_{xxT}\rho_{xxT}}{\rho_{xx}^2} + \mathcal{C}^{sk,2}\frac{\rho_{xx0}}{\rho_{xx}^2}, \tag{255}$$

where ρ_{xx0} is the residual resistivity due to static impurities at low temperatures and $\rho_{xxT} \equiv \rho_{xx} - \rho_{xx0}$ is due to dynamic disorders (mainly phonons in time-reversal symmetric systems at high temperature). Rewrite ρ_{xx} in terms of the two partial conductivities ρ_{xxT} and ρ_{xx0}, we obtain that

$$\begin{aligned} \frac{V_y^{2\omega}}{(V_x^{\omega})^2} &= \frac{1}{\rho_{xx}^2}\Big(\mathcal{C}^{in}\rho_{xx}^2 + \mathcal{C}_0^{sj}\rho_{xx0}\rho_{xx} + \mathcal{C}_1^{sj}\rho_{xxT}\rho_{xx} + \mathcal{C}_{00}^{sk,1}\rho_{xx0}\rho_{xx0} + \mathcal{C}_{01}^{sk,1}\rho_{xx0}\rho_{xxT} + \mathcal{C}_{11}^{sk,1}\rho_{xxT}\rho_{xxT} + \mathcal{C}^{sk,2}\rho_{xx0}\Big) \\ &= \frac{1}{\rho_{xx}^2}\Big(\mathcal{C}_1\rho_{xx0} + \mathcal{C}_2\rho_{xx0}^2 + \mathcal{C}_3\rho_{xx0}\rho_{xxT} + \mathcal{C}_4\rho_{xxT}^2\Big) \end{aligned} \tag{256}$$

with four scaling parameters that can be extracted from experiment

$$\mathcal{C}_1 = \mathcal{C}^{sk,2}, \tag{257}$$
$$\mathcal{C}_2 = \mathcal{C}^{in} + \mathcal{C}_0^{sj} + \mathcal{C}_{00}^{sk,1}, \tag{258}$$
$$\mathcal{C}_3 = 2\mathcal{C}^{in} + \mathcal{C}_0^{sj} + \mathcal{C}_1^{sj} + \mathcal{C}_{01}^{sk,1}, \tag{259}$$
$$\mathcal{C}_4 = \mathcal{C}^{in} + \mathcal{C}_1^{sj} + \mathcal{C}_{11}^{sk,1}. \tag{260}$$

SUPPLEMENTARY METHODS

Calculation of the side-jump related quantities

In this section, we perform the $t \ll v$ expansion for the side-jump related quantities. For the side-jump velocity $\mathbf{v}^{sj}$, we have

$$\begin{aligned} v_x^{sj} &= \int \frac{d^2k'}{(2\pi)^2}\varpi_{\mathbf{k}\mathbf{k}'}^{(2)}\delta r_{\mathbf{k}'\mathbf{k}}^x \\ &\simeq -\frac{n_i V_0^2}{8\pi\hbar}\int_0^\infty k'dk'\int_0^{2\pi} d\phi'\Big(\frac{\sin\theta}{k} + \frac{\sin\theta'}{k'}\Big)\Big(\sin\theta\cos\theta'\sin\phi - \cos\theta\sin\theta'\sin\phi'\Big)\Big[\delta(\varepsilon_{\mathbf{k}}^0 - \varepsilon_{\mathbf{k}'}^0) \\ &\quad + t(k'\cos\phi' - k\cos\phi)\frac{\partial}{\partial\varepsilon_{\mathbf{k}'}^0}\delta(\varepsilon_{\mathbf{k}}^0 - \varepsilon_{\mathbf{k}'}^0)\Big] \\ &= -\frac{n_i V_0^2}{4\hbar}\int_0^\infty dk'\frac{m\sin\theta'}{v\cos\theta'}\Big(\cos\theta + \cos\theta'\Big)\Big[\frac{v}{m}\sin\theta\cos\theta'\sin\phi\delta(\varepsilon_{\mathbf{k}}^0 - \varepsilon_{\mathbf{k}'}^0) \\ &\quad + t\frac{\sin^2\theta\cos\theta'}{\cos\theta}\sin\phi\cos\phi\frac{\partial}{\partial\varepsilon_{\mathbf{k}'}^0}\delta(\varepsilon_{\mathbf{k}}^0 - \varepsilon_{\mathbf{k}'}^0)\Big\} \\ &= -\frac{n_i V_0^2}{4\hbar}\frac{\cos\theta\sin\phi}{v^2}\Big[2v\sin\theta - t\sin^2\theta\cos\phi\Big], \end{aligned} \tag{261}$$

and

$$\begin{aligned}
v_y^{sj} &= \int \frac{d^2k'}{(2\pi)^2}\varpi^{(2)}_{\mathbf{k}\mathbf{k}'}\delta r^y_{\mathbf{k}'\mathbf{k}} \\
&\simeq -\frac{n_i V_0^2}{8\pi\hbar}\int_0^\infty k'dk'\int_0^{2\pi} d\phi'\Big(\frac{\sin\theta}{k}+\frac{\sin\theta'}{k'}\Big)\Big(\cos\theta\sin\theta'\cos\phi'-\sin\theta\cos\theta'\cos\phi\Big)\Big[\delta(\varepsilon^0_{\mathbf{k}}-\varepsilon^0_{\mathbf{k}'}) \\
&\quad +t(k'\cos\phi'-k\cos\phi)\frac{\partial}{\partial\varepsilon^0_{\mathbf{k}'}}\delta(\varepsilon^0_{\mathbf{k}}-\varepsilon^0_{\mathbf{k}'})\Big] \\
&= -\frac{n_i V_0^2}{8\hbar}\int_0^\infty dk'\frac{m\sin\theta'}{v\cos\theta'}\Big(\cos\theta+\cos\theta'\Big)\Big[-\frac{2v}{m}\sin\theta\cos\theta'\cos\phi\delta(\varepsilon^0_{\mathbf{k}}-\varepsilon^0_{\mathbf{k}'}) \\
&\quad +\frac{t}{\cos\theta\cos\theta'}\Big(\cos^2\theta\sin^2\theta'+2\sin^2\theta\cos^2\theta'\cos^2\phi\Big)\frac{\partial}{\partial\varepsilon^0_{\mathbf{k}'}}\delta(\varepsilon^0_{\mathbf{k}}-\varepsilon^0_{\mathbf{k}'})\Big\} \\
&= \frac{n_i V_0^2}{8\hbar}\frac{\cos\theta}{v^2}\Big[4v\sin\theta\cos\phi+t\big(3+\cos^2\theta-2\sin^2\theta\cos^2\phi\big)\Big],
\end{aligned} \tag{262}$$

where we only considered the leading Gaussian contribution to the symmetric scattering rate (see Sec.). One can verify that these equalities reproduce the side-jump velocities in the isotropic case (Eq. 213) at $t=0$.

Now we turn to the simplification of the $\mathbf{O}_{ll'}$ dependent term in the second side-jump contribution to the nonlinear Hall conductivity, which comes from the side-jump induced modification of the distribution function. In the general case, this term can be written as

$$\begin{aligned}
&\frac{e^3}{2\hbar}\sum_{ll'}\tau_l v_l^a O^c_{ll'}[\tau_l\partial^b_{\mathbf{k}}f^{(0)}_l-\tau_{l'}\partial^b_{\mathbf{k}'}f^{(0)}_{l'}] \\
&= \frac{\pi e^3}{\hbar^3}\sum_{ll'}\tau_l\big[|T_{ll'}|^2\delta r^c_{ll'}\partial^a_{\mathbf{k}}\delta(\varepsilon_l-\varepsilon_{l'})\big][\tau_l\partial^b_{\mathbf{k}}f^{(0)}_l-\tau_{l'}\partial^b_{\mathbf{k}'}f^{(0)}_{l'}] \\
&= \frac{\pi e^3}{\hbar^3}\sum_{ll'}\Big\{\partial^a_{\mathbf{k}}\big[\tau_l|T_{ll'}|^2\delta r^c_{ll'}\delta(\varepsilon_l-\varepsilon_{l'})\big]\tau_l\partial^b_{\mathbf{k}}f^{(0)}_l \\
&\quad -\partial^a_{\mathbf{k}}(\tau_l|T_{ll'}|^2\delta r^c_{ll'})\big[\tau_l\partial^b_{\mathbf{k}}f^{(0)}_l-\tau_{l'}\partial^b_{\mathbf{k}'}f^{(0)}_{l'}\big]\delta(\varepsilon_l-\varepsilon_{l'})\Big\} \\
&= -\frac{e^3}{2\hbar^2}\sum_l\tau_l[\partial^a_{\mathbf{k}}(\tau_l v^{sj}_c)+\tilde{\mathcal{M}}^{ac}_l]\partial^b_{\mathbf{k}}f^{(0)}_l,
\end{aligned} \tag{263}$$

where we defined that

$$\tilde{\mathcal{M}}^{ab}_l=\sum_{l'}\big(\tilde{M}^{ab}_{ll'}-\tilde{M}^{ab}_{l'l}\big)\delta(\varepsilon_l-\varepsilon_{l'}) \tag{264}$$

with

$$\tilde{M}^{ab}_{ll'}=\frac{2\pi}{\hbar}\partial^a_{\mathbf{k}}(\tau_l|T_{ll'}|^2\delta r^b_{ll'}). \tag{265}$$

For a constant relaxation time $\tau_l=\tau$, we denote that

$$\tilde{\mathcal{M}}^{ab}_l\Rightarrow\tau\mathcal{M}^{ab}_l\equiv\tau\sum_{l'}\big(M^{ab}_{ll'}-M^{ab}_{l'l}\big)\delta(\varepsilon_l-\varepsilon_{l'}) \tag{266}$$

with

$$M^{ab}_{ll'}=\frac{2\pi}{\hbar}\partial^a_{\mathbf{k}}(|T_{ll'}|^2\delta r^b_{ll'}). \tag{267}$$

For the tilted 2D Dirac model with $t<v$, we can drop the general index and the relevant elements of $M^{ab}_{\mathbf{k}\mathbf{k}'}$ can be obtained as

$$M^{xy}_{\mathbf{k}\mathbf{k}'}=\frac{2\pi}{\hbar}\partial^x_{\mathbf{k}}(|T_{\mathbf{k}\mathbf{k}'}|^2\delta r^y_{\mathbf{k}\mathbf{k}'}), \tag{268}$$

$$M^{yx}_{\mathbf{k}\mathbf{k}'}=\frac{2\pi}{\hbar}\partial^y_{\mathbf{k}}(|T_{\mathbf{k}\mathbf{k}'}|^2\delta r^x_{\mathbf{k}\mathbf{k}'}). \tag{269}$$

Note that

$$\frac{\partial}{\partial k_x} \equiv \frac{v\cos\theta}{m}\Big(\cos\theta\cos\phi\frac{\partial}{\partial\theta} - \frac{\sin\phi}{\sin\theta}\frac{\partial}{\partial\phi}\Big), \tag{270}$$

$$\frac{\partial}{\partial k_y} \equiv \frac{v\cos\theta}{m}\Big(\cos\theta\sin\phi\frac{\partial}{\partial\theta} + \frac{\cos\phi}{\sin\theta}\frac{\partial}{\partial\phi}\Big), \tag{271}$$

thus we have

$$\begin{aligned} M^{xy}_{\mathbf{k}\mathbf{k}'} &= \frac{\pi n_i V_0^2}{2\hbar}\frac{v^2\cos\theta}{m^2}\Big(\cos\theta\cos\phi\frac{\partial}{\partial\theta} - \frac{\sin\phi}{\sin\theta}\frac{\partial}{\partial\phi}\Big)\Big[(\cos\theta+\cos\theta')(\cos\theta\sin\theta'\cos\phi' - \sin\theta\cos\theta'\cos\phi)\Big] \\ &= -\frac{\pi n_i V_0^2 v^2}{2\hbar m^2}\cos\theta\Big(\cos\theta\cos\theta' + \cos^2\theta'\sin^2\phi + \cos^2\theta\cos^2\theta'\cos^2\phi - 2\cos\theta\sin^2\theta\cos\theta'\cos^2\phi \\ &\quad +2\cos^2\theta\sin\theta\sin\theta'\cos\phi\cos\phi' + \cos\theta\sin\theta\cos\theta'\sin\theta'\cos\phi\cos\phi'\Big), \end{aligned} \tag{272}$$

and

$$\begin{aligned} M^{yx}_{\mathbf{k}\mathbf{k}'} &= \frac{\pi n_i V_0^2}{2\hbar}\frac{v^2\cos\theta}{m^2}\Big(\cos\theta\sin\phi\frac{\partial}{\partial\theta} + \frac{\cos\phi}{\sin\theta}\frac{\partial}{\partial\phi}\Big)\Big[(\cos\theta+\cos\theta')(\sin\theta\cos\theta'\sin\phi - \cos\theta\sin\theta'\sin\phi')\Big] \\ &= \frac{\pi n_i V_0^2 v^2}{2\hbar m^2}\cos\theta\Big(\cos\theta\cos\theta' + \cos^2\theta'\cos^2\phi + \cos^2\theta\cos^2\theta'\sin^2\phi - 2\cos\theta\sin^2\theta\cos\theta'\sin^2\phi \\ &\quad +2\cos^2\theta\sin\theta\sin\theta'\sin\phi\sin\phi' + \cos\theta\sin\theta\cos\theta'\sin\theta'\sin\phi\sin\phi'\Big). \end{aligned} \tag{273}$$

Then, the relevant elements of $\mathcal{M}^{ab}_{\mathbf{k}}$ can be obtained as

$$\begin{aligned} \mathcal{M}^{xy}_{\mathbf{k}} &= \int [dk']\big(M^{xy}_{\mathbf{k}\mathbf{k}'} - M^{xy}_{\mathbf{k}'\mathbf{k}}\big)\delta(\varepsilon_{\mathbf{k}} - \varepsilon_{\mathbf{k}'}) \\ &\simeq \int [dk']\big(M^{xy}_{\mathbf{k}\mathbf{k}'} - M^{xy}_{\mathbf{k}'\mathbf{k}}\big)\Big[\delta(\varepsilon^0_{\mathbf{k}} - \varepsilon^0_{\mathbf{k}'}) + t(k'\cos\phi' - k\cos\phi)\frac{\partial}{\partial\varepsilon^0_{\mathbf{k}'}}\delta(\varepsilon^0_{\mathbf{k}} - \varepsilon^0_{\mathbf{k}'})\Big] \\ &= -\frac{n_i V_0^2}{8\hbar m v}\sin\theta\cos^2\theta\Big[3v\sin\theta(1-2\cos^2\phi) + t\cos\phi(6 - 15\sin^2\theta + 2\sin^2\theta\cos^2\phi)\Big], \end{aligned} \tag{274}$$

and

$$\begin{aligned} \mathcal{M}^{yx}_{\mathbf{k}} &= \int [dk']\big(M^{yx}_{\mathbf{k}\mathbf{k}'} - M^{yx}_{\mathbf{k}'\mathbf{k}}\big)\delta(\varepsilon_{\mathbf{k}} - \varepsilon_{\mathbf{k}'}) \\ &\simeq \int [dk']\big(M^{yx}_{\mathbf{k}\mathbf{k}'} - M^{yx}_{\mathbf{k}'\mathbf{k}}\big)\Big[\delta(\varepsilon^0_{\mathbf{k}} - \varepsilon^0_{\mathbf{k}'}) + t(k'\cos\phi' - k\cos\phi)\frac{\partial}{\partial\varepsilon^0_{\mathbf{k}'}}\delta(\varepsilon^0_{\mathbf{k}} - \varepsilon^0_{\mathbf{k}'})\Big] \\ &= -\frac{n_i V_0^2}{8\hbar m v}\sin\theta\cos^2\theta\Big[3v\sin\theta(1-2\cos^2\phi) - 2t\cos\phi(3\cos^2\theta - \sin^2\theta\cos^2\phi)\Big]. \end{aligned} \tag{275}$$

Calculation of the skew-scattering related quantities

In this section, we perform the $t \ll v$ expansion for the skew-scattering related quantities. For the antisymmetric third-order scattering rate, we have that

$$\begin{aligned} \omega^{(3a)}_{\mathbf{k}\mathbf{k}'} &\simeq \frac{n_i V_1^3}{4\hbar}\int_0^\infty k''dk''\int_0^{2\pi} d\phi''\big[\sin\theta\sin\theta'\cos\theta''\sin(\phi-\phi') + \sin\theta'\sin\theta''\cos\theta\sin(\phi'-\phi'') \\ &\quad + \sin\theta\sin\theta''\cos\theta'\sin(\phi''-\phi)\big]\Big[\delta(\varepsilon^0_{\mathbf{k}'} - \varepsilon^0_{\mathbf{k}''}) + t(k''\cos\phi'' - k'\cos\phi')\frac{\partial}{\partial\varepsilon^0_{\mathbf{k}''}}\delta(\varepsilon^0_{\mathbf{k}'} - \varepsilon^0_{\mathbf{k}''})\Big]\delta(\varepsilon^+_{\mathbf{k}} - \varepsilon^+_{\mathbf{k}'}) \\ &= \frac{\pi n_i V_1^3}{2\hbar}\frac{m}{v^3\cos\theta'}\Big[v\sin\theta\sin\theta'\cos\theta'\sin(\phi-\phi') + t(\sin\theta\cos\theta'\sin\phi - \cos\theta\sin\theta'\sin\phi')\Big]\delta(\varepsilon^+_{\mathbf{k}} - \varepsilon^+_{\mathbf{k}'}). \end{aligned} \tag{B1}$$

One can verify that this result reproduces the antisymmetric third-order scattering rate in the isotropic case (Eq. 219) at $t=0$. And for the antisymmetric fourth-order scattering rate, we have that

$$\begin{aligned}\varpi_{\mathbf{kk}'}^{(4a)} \simeq& \frac{n_i^2V_0^4}{8\hbar m}\int_0^\infty k''dk''\int_0^{2\pi}d\phi''(\cos\theta+\cos\theta'+\cos\theta'')\big[\sin\theta\sin\theta'\cos\theta''\sin(\phi-\phi')+\sin\theta'\sin\theta''\cos\theta\sin(\phi'-\phi'')\\&+\sin\theta\sin\theta''\cos\theta'\sin(\phi''-\phi)\big]\Big[\delta(\varepsilon^0_{\mathbf{k}'}-\varepsilon^0_{\mathbf{k}''})+t(k''\cos\phi''-k'\cos\phi')\frac{\partial}{\partial\varepsilon^0_{\mathbf{k}''}}\delta(\varepsilon^0_{\mathbf{k}'}-\varepsilon^0_{\mathbf{k}''})\Big]\delta(\varepsilon^+_{\mathbf{k}}-\varepsilon^+_{\mathbf{k}'})\\=&\frac{\pi n_i^2V_0^4}{8\hbar}\frac{1}{v^3\cos\theta'}\Big\{2v\sin\theta\sin\theta'\cos\theta'(\cos\theta+2\cos\theta')\sin(\phi-\phi')+t\big[(\sin\theta\cos\theta'\sin\phi-\cos\theta\sin\theta'\sin\phi')\\&\cdot(2\cos\theta+3\cos\theta'+\cos^3\theta')-2\sin\theta\sin^2\theta'\cos^2\theta'\cos\phi'\sin(\phi-\phi')\big]\Big\}\delta(\varepsilon^+_{\mathbf{k}}-\varepsilon^+_{\mathbf{k}'}).\end{aligned} \tag{B2}$$

One can verify that this result reproduces the antisymmetric fourth-order scattering rate in the isotropic case (Eq. 220) at $t=0$.

There are several integrals that are relevant with our calculation, such as

$$\begin{aligned}I_x^{(3a)}\equiv&\int[dk']\omega^{(3a)}_{\mathbf{kk}'}(v^x_{\mathbf{k}}-v^x_{\mathbf{k}'})\\\simeq&\frac{n_iV_1^3m}{8\pi\hbar^2v^2}\int_0^\infty k'dk'\int_0^{2\pi}d\phi'\frac{\sin\theta\cos\phi-\sin\theta'\cos\phi'}{\cos\theta'}\Big[v\sin\theta\sin\theta'\cos\theta'\sin(\phi-\phi')\\&+t(\sin\theta\cos\theta'\sin\phi-\cos\theta\sin\theta'\sin\phi')\Big]\Big[\delta(\varepsilon^0_{\mathbf{k}}-\varepsilon^0_{\mathbf{k}'})+t(k'\cos\phi'-k\cos\phi)\frac{\partial}{\partial\varepsilon^0_{\mathbf{k}'}}\delta(\varepsilon^0_{\mathbf{k}}-\varepsilon^0_{\mathbf{k}'})\Big]\\=&-\frac{n_iV_1^3m^2}{8\hbar^2v^4}\frac{\sin^2\theta\sin\phi}{\cos\theta}\Big[v\sin\theta+t\cos\phi(1+\cos^2\theta)\Big],\end{aligned} \tag{B3}$$

and

$$\begin{aligned}I_y^{(3a)}\equiv&\int[dk']\omega^{(3a)}_{\mathbf{kk}'}(v^y_{\mathbf{k}}-v^y_{\mathbf{k}'})\\\simeq&\frac{n_iV_1^3m}{8\pi\hbar^2v^2}\int_0^\infty k'dk'\int_0^{2\pi}d\phi'\frac{\sin\theta\sin\phi-\sin\theta'\sin\phi'}{\cos\theta'}\Big[v\sin\theta\sin\theta'\cos\theta'\sin(\phi-\phi')\\&+t(\sin\theta\cos\theta'\sin\phi-\cos\theta\sin\theta'\sin\phi')\Big]\Big[\delta(\varepsilon^0_{\mathbf{k}}-\varepsilon^0_{\mathbf{k}'})+t(k'\cos\phi'-k\cos\phi)\frac{\partial}{\partial\varepsilon^0_{\mathbf{k}'}}\delta(\varepsilon^0_{\mathbf{k}}-\varepsilon^0_{\mathbf{k}'})\Big]\\=&\frac{n_iV_1^3m^2}{8\hbar^2v^4}\frac{\sin^2\theta}{\cos\theta}\Big[v\sin\theta\cos\phi+t(1+\cos^2\phi+\cos^2\theta\cos^2\phi)\Big],\end{aligned} \tag{B4}$$

in the calculation of the extrinsic skew-scattering contributions. And the integrals

$$\begin{aligned}I_x^{(4a)}\equiv&\int[dk']\omega^{(4a)}_{\mathbf{kk}'}(v^x_{\mathbf{k}}-v^x_{\mathbf{k}'})\\\simeq&\frac{n_i^2V_0^4}{32\pi\hbar^2v^2}\int_0^\infty k'dk'\int_0^{2\pi}d\phi'\frac{\sin\theta\cos\phi-\sin\theta'\cos\phi'}{\cos\theta'}\Big\{2v\sin\theta\sin\theta'\cos\theta'(\cos\theta+2\cos\theta')\sin(\phi-\phi')\\&+t\big[(\sin\theta\cos\theta'\sin\phi-\cos\theta\sin\theta'\sin\phi')(2\cos\theta+3\cos\theta'+\cos^3\theta')-2\sin\theta\sin^2\theta'\cos^2\theta'\cos\phi'\sin(\phi-\phi')\big]\Big\}\\&\cdot\Big[\delta(\varepsilon^0_{\mathbf{k}}-\varepsilon^0_{\mathbf{k}'})+t(k'\cos\phi'-k\cos\phi)\frac{\partial}{\partial\varepsilon^0_{\mathbf{k}'}}\delta(\varepsilon^0_{\mathbf{k}}-\varepsilon^0_{\mathbf{k}'})\Big]\\=&-\frac{n_i^2V_0^4m}{16\hbar^2v^4}\sin^2\theta\sin\phi\Big[3v\sin\theta+t\cos\phi(1+5\cos^2\theta)\Big],\end{aligned} \tag{B5}$$

$$
\begin{aligned}
I_y^{(4a)} &\equiv \int [dk'] \omega_{\mathbf{k}\mathbf{k}'}^{(4a)} (v_{\mathbf{k}}^y - v_{\mathbf{k}'}^y) \\
&\simeq \frac{n_i^2 V_0^4}{32\pi\hbar^2 v^2} \int_0^\infty k' dk' \int_0^{2\pi} d\phi' \frac{\sin\theta\sin\phi - \sin\theta'\sin\phi'}{\cos\theta'} \Big\{ 2v \sin\theta \sin\theta' \cos\theta' (\cos\theta + 2\cos\theta') \sin(\phi - \phi') \\
&\quad + t\big[(\sin\theta\cos\theta'\sin\phi - \cos\theta\sin\theta'\sin\phi')(2\cos\theta + 3\cos\theta' + \cos^3\theta') - 2\sin\theta\sin^2\theta'\cos^2\theta'\cos\phi'\sin(\phi-\phi') \big] \Big\} \\
&\quad \cdot \Big[\delta(\varepsilon_{\mathbf{k}}^0 - \varepsilon_{\mathbf{k}'}^0) + t(k'\cos\phi' - k\cos\phi) \frac{\partial}{\partial \varepsilon_{\mathbf{k}'}^0} \delta(\varepsilon_{\mathbf{k}}^0 - \varepsilon_{\mathbf{k}'}^0) \Big] \\
&= \frac{n_i^2 V_0^4 m}{32\hbar^2 v^4} \sin^2\theta \Big[6v \sin\theta\cos\phi + t(5 + \cos^2\theta + 2\cos^2\phi + 10\cos^2\theta\cos^2\phi) \Big],
\end{aligned}
\tag{B6}
$$

in the calculation of the intrinsic skew-scattering contributions.

SUPPLEMENTARY REFERENCES

* Corresponding author: luhz@sustech.edu.cn, luhaizhou@gmail.com

[1] G. D. Mahan, *Many-Particle Physics* (Plenum Press, 1990).

[2] N. Nagaosa, J. Sinova, S. Onoda, A. H. MacDonald, and N. P. Ong, "Anomalous Hall effect," Rev. Mod. Phys. **82**, 1539 (2010).

[3] N. Sinitsyn, "Semiclassical theories of the anomalous Hall effect," J. Phys.: Condens. Matter **20**, 023201 (2008).

[4] D. Xiao, M. C. Chang, and Q. Niu, "Berry phase effects on electronic properties," Rev. Mod. Phys. **82**, 1959 (2010).

[5] N. Sinitsyn, A. MacDonald, T. Jungwirth, V. Dugaev, and J. Sinova, "Anomalous Hall effect in a two-dimensional Dirac band: The link between the Kubo-Streda formula and the semiclassical Boltzmann equation approach," Phys. Rev. B **75**, 045315 (2007).

[6] J. Schliemann and D. Loss, "Anisotropic transport in a two-dimensional electron gas in the presence of spin-orbit coupling," Phys. Rev. B **68**, 165311 (2003).

[7] C. Xiao, D. Li, and Z. Ma, "Role of band-index-dependent transport relaxation times in anomalous Hall effect," Phys. Rev. B **95**, 035426 (2017).

[8] I. Sodemann and L. Fu, "Quantum nonlinear Hall effect induced by Berry curvature dipole in time-reversal invariant materials," Phys. Rev. Lett. **115**, 216806 (2015).

[9] D. Hou, G. Su, Y. Tian, X. Jin, S. A. Yang, and Q. Niu, "Multivariable scaling for the anomalous Hall effect," Phys. Rev. Lett. **114**, 217203 (2015).

[10] Y. Tian, L. Ye, and X. Jin, "Proper scaling of the anomalous Hall effect," Phys. Rev. Lett. **103**, 087206 (2009).
